# In-depth mass-spectrometry reveals phospho-RAB12 as a blood biomarker of G2019S LRRK2-driven Parkinson’s disease

**DOI:** 10.1093/brain/awae404

**Published:** 2024-12-20

**Authors:** Adriana Cortés, Toan K Phung, Lorena de Mena, Alicia Garrido, Jon Infante, Javier Ruíz-Martínez, Miquel À Galmés-Ordinas, Sophie Glendinning, Jesica Pérez, Ana Roig, Marta Soto, Marina Cosgaya, Valeria Ravasi, Manel Fernández, Alejandro Rubiano-Castro, Ramón Díaz, Haizea Hernández-Eguiazu, Coro Sánchez-Quintana, Ana Vinagre-Aragón, Elisabet Mondragón, Ioana Croitoru, María Rivera-Sánchez, Andrea Corrales-Pardo, María Sierra, Eduardo Tolosa, Cristina Malagelada, Raja S Nirujogi, Joaquín Fernández-Irigoyen, Enrique Santamaría, Dario R Alessi, María J Martí, Mario Ezquerra, Rubén Fernández-Santiago

**Affiliations:** Proteored-ISCIII, Proteomics Platform, Clinical Neuroproteomics Unit, Navarrabiomed, Departamento de Salud, UPNA, IdiSNA, Pamplona ES 31008, Spain; Medical Research Council Protein Phosphorylation and Ubiquitylation Unit, University of Dundee, Dundee DD1 5EH, UK; Lab for Parkinson’s & Other Movement Disorders, Institut d’Investigacions Biomèdiques August Pi i Sunyer (IDIBAPS), Parkinson’s Disease and Movement Disorders Unit, Neurology Service, Hospital Clínic de Barcelona, Institut de Neurociències, Universitat de Barcelona, Centro de Investigación Biomédica en Red sobre Enfermedades Neurodegenerativas (CIBERNED) CB06/05/0018-ISCIII, Barcelona ES 08036, Spain; Lab for Parkinson’s & Other Movement Disorders, Institut d’Investigacions Biomèdiques August Pi i Sunyer (IDIBAPS), Parkinson’s Disease and Movement Disorders Unit, Neurology Service, Hospital Clínic de Barcelona, Institut de Neurociències, Universitat de Barcelona, Centro de Investigación Biomédica en Red sobre Enfermedades Neurodegenerativas (CIBERNED) CB06/05/0018-ISCIII, Barcelona ES 08036, Spain; Neurology Service, University Hospital Marqués de Valdecilla-IDIVAL, Centro de Investigación Biomédica en Red sobre Enfermedades Neurodegenerativas (CIBERNED), Santander ES 39008, Spain; Department of Neurology, Donostia University Hospital, Biogipuzkoa Health Research Institute, Centro de Investigación Biomédica en Red sobre Enfermedades Neurodegenerativas (CIBERNED), San Sebastián ES 20014, Spain; In Silico Medicinal Chemistry, Division of Cancer Therapeutics, The Institute of Cancer Research, London SW7 3RP, UK; Medical Research Council Protein Phosphorylation and Ubiquitylation Unit, University of Dundee, Dundee DD1 5EH, UK; Lab for Parkinson’s & Other Movement Disorders, Institut d’Investigacions Biomèdiques August Pi i Sunyer (IDIBAPS), Parkinson’s Disease and Movement Disorders Unit, Neurology Service, Hospital Clínic de Barcelona, Institut de Neurociències, Universitat de Barcelona, Centro de Investigación Biomédica en Red sobre Enfermedades Neurodegenerativas (CIBERNED) CB06/05/0018-ISCIII, Barcelona ES 08036, Spain; Lab for Parkinson’s & Other Movement Disorders, Institut d’Investigacions Biomèdiques August Pi i Sunyer (IDIBAPS), Parkinson’s Disease and Movement Disorders Unit, Neurology Service, Hospital Clínic de Barcelona, Institut de Neurociències, Universitat de Barcelona, Centro de Investigación Biomédica en Red sobre Enfermedades Neurodegenerativas (CIBERNED) CB06/05/0018-ISCIII, Barcelona ES 08036, Spain; Lab for Parkinson’s & Other Movement Disorders, Institut d’Investigacions Biomèdiques August Pi i Sunyer (IDIBAPS), Parkinson’s Disease and Movement Disorders Unit, Neurology Service, Hospital Clínic de Barcelona, Institut de Neurociències, Universitat de Barcelona, Centro de Investigación Biomédica en Red sobre Enfermedades Neurodegenerativas (CIBERNED) CB06/05/0018-ISCIII, Barcelona ES 08036, Spain; Lab for Parkinson’s & Other Movement Disorders, Institut d’Investigacions Biomèdiques August Pi i Sunyer (IDIBAPS), Parkinson’s Disease and Movement Disorders Unit, Neurology Service, Hospital Clínic de Barcelona, Institut de Neurociències, Universitat de Barcelona, Centro de Investigación Biomédica en Red sobre Enfermedades Neurodegenerativas (CIBERNED) CB06/05/0018-ISCIII, Barcelona ES 08036, Spain; Lab for Parkinson’s & Other Movement Disorders, Institut d’Investigacions Biomèdiques August Pi i Sunyer (IDIBAPS), Parkinson’s Disease and Movement Disorders Unit, Neurology Service, Hospital Clínic de Barcelona, Institut de Neurociències, Universitat de Barcelona, Centro de Investigación Biomédica en Red sobre Enfermedades Neurodegenerativas (CIBERNED) CB06/05/0018-ISCIII, Barcelona ES 08036, Spain; Lab for Parkinson’s & Other Movement Disorders, Institut d’Investigacions Biomèdiques August Pi i Sunyer (IDIBAPS), Parkinson’s Disease and Movement Disorders Unit, Neurology Service, Hospital Clínic de Barcelona, Institut de Neurociències, Universitat de Barcelona, Centro de Investigación Biomédica en Red sobre Enfermedades Neurodegenerativas (CIBERNED) CB06/05/0018-ISCIII, Barcelona ES 08036, Spain; Lab for Parkinson’s & Other Movement Disorders, Institut d’Investigacions Biomèdiques August Pi i Sunyer (IDIBAPS), Parkinson’s Disease and Movement Disorders Unit, Neurology Service, Hospital Clínic de Barcelona, Institut de Neurociències, Universitat de Barcelona, Centro de Investigación Biomédica en Red sobre Enfermedades Neurodegenerativas (CIBERNED) CB06/05/0018-ISCIII, Barcelona ES 08036, Spain; Proteored-ISCIII, Proteomics Platform, Clinical Neuroproteomics Unit, Navarrabiomed, Departamento de Salud, UPNA, IdiSNA, Pamplona ES 31008, Spain; Department of Neurology, Donostia University Hospital, Biogipuzkoa Health Research Institute, Centro de Investigación Biomédica en Red sobre Enfermedades Neurodegenerativas (CIBERNED), San Sebastián ES 20014, Spain; Neurology Service, University Hospital Marqués de Valdecilla-IDIVAL, Centro de Investigación Biomédica en Red sobre Enfermedades Neurodegenerativas (CIBERNED), Santander ES 39008, Spain; Department of Neurology, Donostia University Hospital, Biogipuzkoa Health Research Institute, Centro de Investigación Biomédica en Red sobre Enfermedades Neurodegenerativas (CIBERNED), San Sebastián ES 20014, Spain; Department of Neurology, Donostia University Hospital, Biogipuzkoa Health Research Institute, Centro de Investigación Biomédica en Red sobre Enfermedades Neurodegenerativas (CIBERNED), San Sebastián ES 20014, Spain; Department of Neurology, Donostia University Hospital, Biogipuzkoa Health Research Institute, Centro de Investigación Biomédica en Red sobre Enfermedades Neurodegenerativas (CIBERNED), San Sebastián ES 20014, Spain; Neurology Service, University Hospital Marqués de Valdecilla-IDIVAL, Centro de Investigación Biomédica en Red sobre Enfermedades Neurodegenerativas (CIBERNED), Santander ES 39008, Spain; Neurology Service, University Hospital Marqués de Valdecilla-IDIVAL, Centro de Investigación Biomédica en Red sobre Enfermedades Neurodegenerativas (CIBERNED), Santander ES 39008, Spain; Neurology Service, University Hospital Marqués de Valdecilla-IDIVAL, Centro de Investigación Biomédica en Red sobre Enfermedades Neurodegenerativas (CIBERNED), Santander ES 39008, Spain; Lab for Parkinson’s & Other Movement Disorders, Institut d’Investigacions Biomèdiques August Pi i Sunyer (IDIBAPS), Parkinson’s Disease and Movement Disorders Unit, Neurology Service, Hospital Clínic de Barcelona, Institut de Neurociències, Universitat de Barcelona, Centro de Investigación Biomédica en Red sobre Enfermedades Neurodegenerativas (CIBERNED) CB06/05/0018-ISCIII, Barcelona ES 08036, Spain; Department of Biomedicine, Faculty of Medicine, Universitat de Barcelona, Institut de Neurociències, Universitat de Barcelona, Centro de Investigación Biomédica en Red sobre Enfermedades Neurodegenerativas (CIBERNED), Barcelona ES 08036, Spain; Medical Research Council Protein Phosphorylation and Ubiquitylation Unit, University of Dundee, Dundee DD1 5EH, UK; Proteored-ISCIII, Proteomics Platform, Clinical Neuroproteomics Unit, Navarrabiomed, Departamento de Salud, UPNA, IdiSNA, Pamplona ES 31008, Spain; Proteored-ISCIII, Proteomics Platform, Clinical Neuroproteomics Unit, Navarrabiomed, Departamento de Salud, UPNA, IdiSNA, Pamplona ES 31008, Spain; Medical Research Council Protein Phosphorylation and Ubiquitylation Unit, University of Dundee, Dundee DD1 5EH, UK; Lab for Parkinson’s & Other Movement Disorders, Institut d’Investigacions Biomèdiques August Pi i Sunyer (IDIBAPS), Parkinson’s Disease and Movement Disorders Unit, Neurology Service, Hospital Clínic de Barcelona, Institut de Neurociències, Universitat de Barcelona, Centro de Investigación Biomédica en Red sobre Enfermedades Neurodegenerativas (CIBERNED) CB06/05/0018-ISCIII, Barcelona ES 08036, Spain; Lab for Parkinson’s & Other Movement Disorders, Institut d’Investigacions Biomèdiques August Pi i Sunyer (IDIBAPS), Parkinson’s Disease and Movement Disorders Unit, Neurology Service, Hospital Clínic de Barcelona, Institut de Neurociències, Universitat de Barcelona, Centro de Investigación Biomédica en Red sobre Enfermedades Neurodegenerativas (CIBERNED) CB06/05/0018-ISCIII, Barcelona ES 08036, Spain; Lab for Parkinson’s & Other Movement Disorders, Institut d’Investigacions Biomèdiques August Pi i Sunyer (IDIBAPS), Parkinson’s Disease and Movement Disorders Unit, Neurology Service, Hospital Clínic de Barcelona, Institut de Neurociències, Universitat de Barcelona, Centro de Investigación Biomédica en Red sobre Enfermedades Neurodegenerativas (CIBERNED) CB06/05/0018-ISCIII, Barcelona ES 08036, Spain

**Keywords:** Parkinson’s disease, leucine-rich repeat kinase 2, peripheral blood mononuclear cells, phospho-/proteomics, non-manifesting carriers, biomarker

## Abstract

Leucine-rich repeat kinase 2 (*LRRK2*) inhibition is a promising disease-modifying therapy for LRRK2-associated Parkinson’s disease (L2PD) and idiopathic PD. However, pharmacodynamic readouts and progression biomarkers for clinical trials aiming for disease modification are insufficient, given that no endogenous marker reflecting enhanced kinase activity of the most common LRRK2 G2019S mutation has yet been reported in L2PD patients.

Using phospho-/proteomic analyses, we assessed the impact of LRRK2-activating mutations in peripheral blood mononuclear cells from an LRRK2 clinical cohort from Spain (*n* = 174). The study groups encompassed G2019S L2PD patients (*n* = 37), non-manifesting LRRK2 mutation carriers of G2019S (here termed G2019S L2NMCs) (*n* = 27), R1441G L2PD patients (*n* = 14), R1441G L2NMCs (*n* = 11), idiopathic PD patients (*n* = 40) and healthy controls (*n* = 45).

We identified 207 differentially regulated proteins in G2019S L2PD compared with controls (39 upregulated and 168 downregulated) and 67 in G2019S L2NMCs (10 upregulated and 57 downregulated). G2019S downregulated proteins affected the endolysosomal pathway, proteostasis and mitochondria, e.g. ATIC, RAB9A or LAMP1. At the phospho-proteome level, we observed increases in endogenous phosphorylation levels of pSer106 RAB12 in G2019S carriers, which were validated by immunoblotting after 1 year of follow-up (*n* = 48). Freshly collected peripheral blood mononuclear cells from three G2019S L2PD, one R1441G L2PD, one idiopathic PD and five controls (*n* = 10) showed strong diminishment of pSer106 RAB12 phosphorylation levels after *in vitro* administration of the MLi-2 LRRK2 inhibitor. Using machine learning, we identified an 18-feature G2019S phospho-/protein signature discriminating G2019S L2PD, L2NMCs and controls with 96% accuracy that was correlated with disease severity, i.e. UPDRS-III motor scoring.

Using easily accessible peripheral blood mononuclear cells from a LRRK2 clinical cohort, we identified elevated levels of pSer106 RAB12 as an endogenous biomarker of G2019S carriers. Our data suggest that monitoring pSer106 RAB12 phosphorylation could be a relevant biomarker for tracking LRRK2 activation, particularly in G2019S carriers. Future work might determine whether pSer106 RAB12 could help with patient enrichment and monitoring drug efficacy in LRRK2 clinical trials.

## Teaser

The LRRK2 activating mutation G2019S is the most frequent genetic cause of Parkinson’s disease. Through phospho-proteome analysis of blood, Cortés *et al.* identify elevated phospho-RAB12 levels as an endogenous biomarker of G2019S mutation carriers, with potential utility in clinical trials.

## Introduction

Activating mutations in the leucine-rich repeat kinase 2 (*LRRK2*), e.g. G2019S at the kinase or R1441G at the GTPase domains, increase LRRK2 kinase activity^1-4^ causing autosomal-dominant LRRK2 Parkinson’s disease (L2PD).^5,6^ By converging pathways, LRRK2 kinase activity appears also to be enhanced in patients with idiopathic PD (iPD),^7-9^ which is clinically indistinguishable from L2PD.^10,11^ Thus, ongoing clinical trials of small-molecule type-I inhibitors targeting active LRRK2 protein conformation represent a promising disease-modifying strategy for a broad spectrum of patients.^12,13^ LRRK2 non-manifesting carriers (L2NMCs) are at high risk of PD in an age-dependent progressive manner,^14-16^ encompassing a candidate population for the continued clinical follow-up and disease course modification by early neuroprotective interventions.^13^

A subset of G-proteins from the Ras-related small GTPase superfamily^17^ was reported as phosphorylation substrates of the LRRK2 Ser/Thr kinase.^2,3^ Among these, pThr73 RAB10 was validated as an LRRK2 substrate^18^ showing elevated endogenous phosphorylation levels in a large set of R1441G carriers, PD-manifesting and non-manifesting, but not in G2019S subjects.^19^ Moreover, pThr73 RAB10 represents a readout for LRRK2 pharmacological inhibition using Mli-2 or DNL201.^20,21^ In addition, RAB29^22,23^ and, more recently, RAB12^24,25^ and RAB32,^26^ have been described as critical upstream LRRK2 activators. Despite significant progress, there is an urgent need for clinical biomarkers of progression and robust pharmacodynamic readouts useful for disease modification clinical trials.

By data-independent acquisition (DIA) mass spectrometry (MS), we have screened the LRRK2 phospho-/proteome using peripheral blood mononuclear cells (PBMCs) from an extensive LRRK2 clinical cohort (*n* = 174) including G2019S L2PD (*n* = 37), G2019S L2NMCs (*n* = 27), R1441G L2PD (*n* = 14), R1441G L2NMCs (*n* = 11), iPD (*n* = 40) and controls (*n* = 45). We identified differential phospho-/proteins in G2019S and R1441G carriers, PD-manifesting and non-manifesting. More specifically we detected elevated pSer106 RAB12 phosphorylation levels in G2019S carriers. Our results suggest that pSer106 RAB12 is an endogenous biomarker of G2019S, which can also be elevated in G2019S L2PD and L2NMCs. Consistent with RAB12 being phosphorylated by LRRK2, we found that pSer106 RAB12 levels strongly diminished after MLi-2 LRRK2 inhibition in all kinds of subjects, regardless of disease or mutation status. We propose that pSer106 RAB12 could be exploited as a marker of LRRK2 activity in clinical trials.^13^ Following the FAIR principles of data findability, accessibility, interoperability, and reusability^27^ and through interactive Curtain weblinks for non-MS experts,^28^ we provide full open access to all data generated in this study.

## Materials and methods

### Subjects

Probands participated in the study after ethics approval and signed informed consent. Subjects included symptomatic and asymptomatic *LRRK2* mutation carriers, iPD patients and controls, who were healthy spouses and companions of Spanish descent. Patient inclusion criteria were a clinical diagnosis of PD by a movement disorders specialist based on the Movement Disorder Society (MDS) criteria for Parkinson’s disease.^29^ Exclusion criteria were chronic inflammatory and autoimmune diseases, e.g. Crohn’s disease, inflammatory bowel disease, rheumatoid arthritis, systemic lupus erythematosus, chronic neurological diseases such as myasthenia gravis, chronic use of non-steroidal anti-inflammatory drugs or corticosteroid anti-inflammatory medication, and viral or bacterial infection during the week before to blood donation. Subjects were recruited at three centres in Spain: Hospital Clínic de Barcelona (*n* = 76; ‘B’),^30^ Hospital Marqués de Valdecilla in Santander (*n* = 55; ‘S’)^31^ and Hospital de Donostia in San Sebastian (*n* = 43; ‘D’)^32^ (Table 1). By cohort and subject type, the sample included G2019S L2PD (*n* = 37; 16 from centre B, 20 from S and 1 from D), G2019S L2NMCs (*n* = 27; 11 from B, 15 S and 1 D), R1441G L2PD (*n* = 14; 1 from B and 13 D), R1441G L2NMCs (*n* = 11; 3 from B and 8 D), iPD (*n* = 40; 20 from B, 10 S and 10 D) and controls (*n* = 45; 25 from B, 10 S and 10 D). We also collected sex, age at sampling, age at onset, LRRK2 mutation status, kinship to index cases, MDS-Unified Parkinson's Disease Rating Scale part III (UPDRS-III),^33^ Montreal Cognitive Assessment (MoCA),^34^ autoimmune and environmental questionnaires, and coronavirus disease 2019 (COVID-19) history. PD patients had a mean age at sampling of 63.5 years for G2019S L2PD, 67.1 years for R1441G L2PD and 67.3 years for iPD. Asymptomatic blood relatives of L2PD patients, i.e. L2NMCs, were younger than PD patients, at an average of 56.7 years for G2019S L2NMCs and 61.1 years for R1441G L2NMCs. The age at onset was similar for G2019S and R1441G L2PD, at 55.1 and 55.8 years, respectively, whereas for iPD it was 62.1 years. Mean disease duration was 8.4 years for G2019S L2PD, 12.3 years for R1441G L2PD and 5.2 years for IPD. Average disease severity, UPDRS-III motor scoring, was similar (mild) in all three patient groups, i.e. 16.0 in G2019S L2PD, 19.8 R1441G L2PD and 19.7 for iPD. Mean MoCA scores were also mild and similar in all patients: 24.3 for G2019S L2PD, 23.2 for R1441G L2PD and 25.6 for IPD. Average L-DOPA equivalent daily dose was 635.8 mg for G2019S L2PD, 711.5 mg for R1441G L2PD and 584.7 mg for iPD.

**Table 1:** Participant clinical features and demographics

Cohort	*n* (males/females)	Age at sampling (years)	PD AAO (years)	Disease duration (years)	UPDRS-III	H&Y	MoCA	LEDD (mg)	Past COVID-19 (yes/no)
**Entire cohort**	**174**								
G2019S L2PD	37 (20/17)	63.5 ± 9.1 (37/37)	55.1 ± 10.2 (33/37)	8.4 ± 6.3 (33/37)	16.0 ± 9.7 (34/37)	2.0 ± 0.6 (21/37)	24.3 ± 4.5 (35/37)	635.8 ± 438.8 (29/39)	4/30 (34/37)
G2019S L2NMC	27 (18/9)	56.7 ± 14.1 (26/27)	–	–	1.0 ± 1.6 (22/27)	–	25.4 ± 6.6 (25/27)	–	6/19 (25/27)
R1441G L2PD	14 (7/7)	67.1 ± 9.5 (14/14)	55.8 ± 11.4 (14/14)	12.3 ± 5.5 (14/14)	19.8 ± 12.0 (14/14)	2.2 ± 0.9 (13/14)	23.2 ± 5.5 (10/14)	711.5 ± 355.7 (14/14)	4/10 (14/14)
R1441G L2NMC	11 (4/7)	61.1 ± 5.5 (11/11)	–	–	1.2 ± 2.1 (11/11)	–	28.6 ± 2.0 (11/11)	–	1/10 (11/11)
iPD	40 (30/10)	67.3 ± 7.7 (40/40)	62.1 ± 8.5 (40/40)	5.2 ± 4.3 (40/40)	19.7 ± 13.2 (40/40)	2.2 ± 0.6 (31/40)	25.6 ± 3.7 (33/40)	584.7 ± 373.6 (37/40)	1/37 (38/40)
C	45 (18/27)	60.0 ± 10.9 (45/45)	–	–	1.2 ± 2.2 (17/27)	–	27.5 ± 3.2 (27/27)	–	14/30 (44/45)
**B—Barcelona**	**76**								
G2019S L2PD	16 (7/9)	65.5 ± 8.3 (16/16)	53.5 ± 11.3 (14/16)	11.4 ± 7.1 (14/16)	13.0 ± 7.2 (13/16)	2.0 ± 0.5 (11/16)	25.0 ± 4.3 (14/16)	596.5 ± 269.7 (13/18)	2/12 (14/16)
G2019S L2NMC	11 (7/4)	47.8 ± 15.5 (10/11)	–	–	0.3 ± 0.7 (10/11)	–	28.2 ± 2.0 (10/11)	–	4/5 (9/11)
R1441G L2PD	1 (0/1)	44.0 (1/1)	32.0 (1/1)	12.0 (1/1)	16.0 (1/1)	NA (0/1)	30.0 (1/1)	400 (1/1)	0/1 (1/1)
R1441G L2NMC	3 (2/1)	65.3 ± 1.5 (3/3)	–	–	3.0 ± 3.6 (3/3)	–	29.3 ± 0.6 (3/3)	–	1/2 (3/3)
iPD	20 (16/4)	68.3 ± 7.9 (20/20)	64.3 ± 7.8 (20/20)	4.0 ± 3.0 (20/20)	14.7 ± 4.2 (20/20)	1.9 ± 0.2 (20/20)	27.2 ± 3.2 (20/20)	453.8 ± 279.8 (19/20)	1/19 (20/20)
C	25 (9/16)	63.9 ± 10.6 (25/25)	–	–	1.7 ± 2.4 (16/25)	–	27.9 ± 2.1 (25/25)	–	8/17 (25/25)
**S—Santander**	**55**								
G2019S L2PD	20 (13/0)	61.3 ± 9.0 (20/20)	55.6 ± 9.1 (18/20)	6.1 ± 4.7 (18/20)	18.1 ± 11.0 (20/20)	1.9 ± 0.6 (9/20)	24.2 ± 4.6 (20/20)	652.7 ± 562.3 (15/20)	2/17 (19/20)
G2019S L2NMC	15 (11/4)	62.6 ± 10.2 (15/15)	–	–	1.8 ± 1.9 (11/15)	–	23.3 ± 8.1 (14/15)	–	2/13 (15/15)
iPD	10 (6/4)	67.2 ± 7.6 (10/10)	62.0 ± 6.3 (10/10)	5.2 ± 4.0 (10/10)	17.9 ± 8.3 (10/10)	2.5 (1/10)	23 ± 3.5 (10/10)	479.9 ± 220.2 (8/10)	0/8 (8/10)
C	10 (4/6)	56.9 ± 11.1 (10/10)	–	–	NA	–	25.0 ± 3.6 (6/6)	–	1/8 (9/10)
**D—Donostia**	**43**								
G2019S L2PD	1 (0/1)	78.0 (1/1)	68.0 (1/1)	10.0 (1/1)	15.0 (1/1)	3.0 (1/1)	19.0 (1/1)	893 (1/1)	0/1 (1/1)
G2019S L2NMC	1 (0/1)	58.0 (1/1)	–	–	NA	–	29 (1/1)	–	0/1 (1/1)
R1441G L2PD	13 (7/6)	68.9 ± 7.1 (13/13)	56.5 ± 9.7 (13/13)	12.4 ± 5.7 (13/13)	20.1 ± 12.5 (13/13)	2.2 ± 0.9 (13/13)	22.4 ± 5.2 (9/13)	735.5 ± 358.3 (13/13)	4/9 (13/13)
R1441G L2NMC	8 (2/6)	59.5 ± 5.6 (8/8)	–	–	0.5 ± 0.8 (6/6)	–	28.3 ± 2.3 (6/6)	–	0/8 (8/8)
iPD	10 (8/2)	65.4 ± 7.8 (10/10)	57.9 ± 10.6 (10/10)	7.5 ± 5.9 (10/10)	31.7 ± 20.7 (10/10)	2.7 ± 0.7 (10/10)	23.7 ± 2.9 (10/10)	917.3 ± 441.8 (10/10)	0/10 (10/10)
C	10 (5/5)	53.5 ± 8.1 (10/10)	–	–	NA	–	29.1 ± 3.9 (5/5)	–	5/5 (10/10)

Data are expressed as the mean ± standard deviation (SD), with the number of available subjects/totals in parentheses. AAO = age at onset; C = controls; H&Y = Hoehn & Yahr scale for severity; iPD = idiopathic PD; LEDD = levodopa equivalent daily dose; L2NMC = *LRRK2* non-manifesting carriers; L2PD = LRRK2-associated PD patients; MoCA = Montreal cognitive assessment; PD = Parkinson's disease; UPDRS-III = Unified Parkinson’s Disease Rating Scale; – = not applicable; NA = not available.

### Genotyping

We genotyped the most common *LRRK2* mutations in our population using Taqman SNP assays-on-demand for *LRRK2* G2019S (Thermo Fisher Scientific Catalogue No. C-63498123-10) and a commercial TaqMan assay for *LRRK2* R1441G^35^ on a Step-One Plus Real-time PCR System (Life Technologies Inc.).

### Peripheral blood mononuclear cell isolation

Forty millilitres of peripheral blood was drawn early in the morning in the fasting state, and PBMCs were isolated by density gradient using sodium citrate tubes (BD Vacutainer CPT, EAN30382903627821) following the manufacturer’s instructions. As is usual in large-scale proteomic studies, dry peripheral blood mononuclear cell (PBMC) pellets were isolated at every patient visit, immediately snap-frozen into liquid N_2_, stored at −80°C overnight, and cryopreserved at −196°C in liquid N_2_ for long storage (half a year on average) until data-independent acquisition (DIA)-MS.

### Peripheral blood mononuclear cell preparation

PBMC samples from the three cohorts were processed in parallel. Blind experimental groups to the operator were balanced and randomized in runs to avoid manipulation bias. Briefly, PBMCs were homogenized in lysis buffer (7 M urea, 2 M thiourea and 50 mM dithiothreitol) supplemented with complete mini protease (Roche Cat. No. 11836153001) and PhosSTOP phosphatase (Roche Cat. No. 4906845001) inhibitors. Lysates were centrifuged at 20 000*g*, 15°C for 1 h, and the resulting supernatant was quantified by the Bradford assay (Bio-Rad Cat. No. 5000201). At least 400 µg of protein was separated for protein digestion to obtain phosphorylated fractions. Proteins were reduced with dithiothreitol (final concentration of 20 mM; 30 min at room temperature), alkylated with iodoacetamide (final concentration of 30 mM; 30 min in the dark at room temperature), diluted to 0.9 M with ammonium bicarbonate (ABC) buffer, and digested with trypsin (Promega Cat. No. V5280; 1:20 w/w enzyme protein ratio, 37°C for 18 h). Protein digestion was interrupted by acidification (acetic acid, pH < 6), and the resulting peptides were cleaned up using Pierce Peptide Desalting Spin Columns (Thermo Fisher Scientific Cat. No. 89851). Phospho-peptide enrichment was performed using the High-Select TiO_2_ Phospho-peptide enrichment Kit (Thermo Fisher Scientific Cat. No. A32993) according to the manufacturer’s instructions. Finally, the phospho-enriched fractions were cleaned up as described above and dried down in a speed vacuum system. Aliquots of 10 µg cleaned-up peptides from protein digestions were set aside for total protein analyses.

### Data-independent acquisition mass spectrometry

Dried-down peptide samples were reconstituted with 2% acetonitrile and 0.1% formic acid, spiked with internal retention time peptide standards (Biognosys) and quantified by NanoDropTM spectrophometer (ThermoFisher Scientific) before liquid chromatography–tandem mass spectrometry (MS/MS) in an EASY-1000 nanoLC system coupled to an EZ-Exploris 480 mass spectrometer (Thermo Fisher Scientific). Peptides were resolved using a C18 Aurora column (75 µm × 25 cm, 1.6 µm particles; IonOpticks) at a flow rate of 300 nl/min using a 60-min gradient (50°C): from 2% to 5% B in 1 min, from 5% to 20% B in 48 min, from 20% to 32% B in 12 min, and from 32% to 95% B in 1 min (where A = 0.1% formic acid and B = 100% acetonitrile:0.1% formic acid). Peptides were ionized using 1.6 kV spray voltage at a capillary temperature of 275°C. We used DIA with full MS scans (scan range: 400 to 900 *m/z*; resolution: 60 000; maximum injection time: 22 ms; normalized automatic gain control (AGC) target: 300%) and 24 periodical MS/MS segments applying 20 Th isolation windows (0.5 Th overlap; resolution: 15 000; maximum injection time: 22 ms; normalized AGC target: 100%). Peptides were fragmented using a normalized HCD (Higher-energy Collisional Dissociation) collision energy of 30%. MS data files were analysed using Spectronaut (Biognosys) by direct DIA analysis. MS/MS spectra were searched against the Uniprot proteome reference from the *Homo sapiens* database UP000005640 using standard settings. The enzyme was set to trypsin in a specific mode. On the one hand, carbamidomethyl (C) was set as a fixed modification, and oxidation (M), acetyl (protein N-term), deamidation (N) and Gln to pyroGlu as variable modifications for total protein analysis. On the other hand, carbamidomethyl (C) was set as a fixed modification, and oxidation (M), acetyl (protein N-term) and Phospho (STY) were used as variable modifications for phospho-proteome analysis. Identifications were filtered by a 1% *Q*-value. After MS, samples that did not pass quality control were omitted from the study, resulting in a sample of G2019S L2PD (*n* = 32; 15 from B and 17 S), G2019S L2NMCs (*n* = 22; 9 from B and 13 S), R1441G L2PD (*n* = 13; 1 from B and 12 D), R1441G L2NMCs (*n* = 7; 2 from B and 5 D), iPD patients (*n* = 39; 19 from B, 10 S and 10 D) and healthy controls (*n* = 42; 23 from B, 10 S and 9 D). Lastly, to disambiguate peptide identities into gene names we used the Uniprot online database (https://uniprotparser.proteo.info/). The resulting number of proteins (3815) and phospho-peptides (10 288) identified by DIA-MS in human PBMCs attest to the optimal experimental quality of the LRRK2 clinical samples.

### Proteome differential analysis

Proteome MS output data were exported from .SNE files from Spectronaut in a pivot table text format. For the differential analyses between groups, MS data were processed using QFeatures (doi:10.18129/B9.bioc.QFeatures) in R (QFeatures v.1.13.1). We applied the following R workflow. First, data were filtered to remove proteins identified by only one peptide sequence. Second, data selection was done based on condition and sub-group labels, with overall analysis containing all samples, G2019S analyses containing Barcelona and Santander samples labelled with the prefix ‘B’ or ‘S’, and R1441G analyses containing samples labelled with the prefix ‘D’ from Donostia-San Sebastian and ‘B’ from Barcelona if carrying R1441G. For each analysis, we provided a separate Rscript file with a customized group selection, in addition to a single collapsing file with all scripts available in the Supplementary material and as a cloud weblink (doi:10.5281/zenodo.13774022). Third, a protein identity (ID) column was assigned as an identification column for the analysis at QFeatures. Fourth, we filtered out any row with ≥70% missing data. Here, with a 70% missing data cut-off, a meta-analysis would have 3815 rows, whereas a more common 30% missing data cut-off would result in 3789 rows. Given that there was only ∼0.71% difference between the cut-off thresholds, we chose the 70% cut-off to keep entries potentially found in only one group without affecting the statistical power of the entire analysis. Fifth, the imputation of missing data was done using the K-Nearest Neighbors (KNN) method (QFeatures v.1.13.1). Subsequently, (sixth) we performed a log_2_ transformation of the imputed data matrix, and (seventh) designed a contrast matrix for differential analysis using limma.^36^ Eighth, for each contrast matrix, we performed a limma analysis using the Benjamini–Hochberg false discovery rate (FDR) multiple-testing adjustment under a statistical significance of an FDR adjusted *P* < 0.05 (1.12 log_10_) and a log_2_ fold-change (FC) > |0.6| (|1.5| in lineal values). Scripts for proteome raw data download and re-analysis are available online (Supplementary material). For ANOVA, we used the normalized data from above as a starting point. The data from each row were grouped depending on the criteria used for grouping. Then, for each comparison, we applied a Python script using one-way ANOVA on the grouped data within the comparison and returned the *P*-value output as a new column.^37^ Lastly, we performed the same FDR correction as above to obtain the multiple-testing adjusted *P*-values using the Statsmodels Python package^38^ with Python scripts also available online (Supplementary material).

### Phospho-proteome differential analysis

Phospho-proteome MS data were exported from .SNE files from Spectronaut in a long-form table format using a Spectronaut param export file available online (Supplementary material). Data were imputed using a modified version of a collapsing R script (Perseus Plugin Peptide Collapse),^39^ with phosphorylation as target modification at a confidence cut-off of >0.75. Modified collapsing.R and Perseus parameter.xml files are available online (Supplementary material). We applied the following R workflow. First, columns with >70% blank cells were removed to meet the KNN requirement of <80% blank columns. Second, data selection for QFeatures input was based on condition and sub-group labels using all samples for overall analysis or specific group combination for location-specific and mutation-specific group combination, with overall analysis containing all samples, G2019S analyses containing Barcelona and Santander samples labelled with the prefix ‘B’ or ‘S’, and R1441G analyses containing samples labelled with the prefix ‘D’ from Donostia-San Sebastian and ‘B’ from Barcelona when carrying R1441G. For each phospho-analysis group, we provide a separate Rscript file with customization to the selection group (Supplementary material). Subsequently, (third) we performed imputation by removing any row with ≥30% empty data, similar to the proteome analysis using the KNN method, and (fourth) performed log_2_ transformation normalization of the data using the quantile normalization method. Fifth, the statistical significance criteria were set at an FDR multiple-testing adjusted *P* < 0.05 (1.12 in log_10_) and a log_2_FC > |0.6| (|1.5| in lineal values). Seventh, in each differential analysis, we matched the protein and its original sequence using protein UniProt ID and extracted the post-translational modification (PTM) position in protein and peptide, the peptide sequence and the sequence window for visualization at the Curtain tool.^28^ Scripts for phospho-proteome data re-analysis are available in the Supplementary material and as a cloud weblink (doi:10.5281/zenodo.13774022). For phospho-proteome ANOVA, we followed the same methodology as the proteome analysis, using the normalized phospho-proteome datasets from above. Data belonging to each group were identified from their column name. One-way ANOVA was applied on each row of cell groups from their respective comparison. The final statistically significant output values were adjusted using the Statsmodels package under the same FDR multiple-testing adjusted *P* < 0.05. Python scripts for ANOVA phospho-proteome analyses are available in the Supplementary material.

### Data visualization

Aligning to FAIR principles^27^ of data findability, accessibility, interoperability and reusability, we used Curtain and Curtain PTM,^28^ as free open-source tools for MS phospho-/proteomics data mining and exploitation by MS non-experts. Visualization of each of the differential analysis results from limma was done in volcano plot representation using the default cut-off settings of a FC > |1.5| (|0.6| log_2_) and an FDR multiple-testing adjusted *P* < 0.05 (1.12 in log_10_). The Curtain tools enable interactive perusal of volcano plots, deconvoluting primary experimental data to individual replicates that can be visualized in bar charts or violin plots, allowing statistical analysis and export of plots in .SVG format (Curtain tutorials). For each analysis, we also provide web links in the figure legends. From each link, users can view the data associated with each data point on the volcano plot as bar charts and violin plots. The magnitude of the data within these plots represents the relative intensity of the protein (total proteome) or phospho-site (phospho-proteome) before normalization. Beyond simple visualization of the numerical data, Curtain tools aggregate data for different knowledge bases, including UniProt, AlphaFold, PhosphoSitePlus, ProteomicsDB and StringDB.

### Machine learning modelling of G2019S differential phospho-/proteins

The normalized and imputed datasets comprising differentially expressed peptides and phospho-peptides were used to train a multi-class classifier to distinguish between controls, G2019S L2PD and G2019S L2NMCs. Three distinct candidate models were considered, including support vector machine (SVM), random forest and gradient boosting classifiers, as described in other studies.^40^ Parameter optimization of the models was done through a grid search with a 5-fold cross-validation. To mitigate potential performance degradation attributable to unbalanced group sizes, we applied the synthetic minority over-sampling technique (SMOTE)^41^ to the training split. We used the balanced accuracy score,^42^ defined as the average recall across each class, to evaluate model performances. Implementation of the models was done using the Scikit-learn^43^ v.1.3.1 library within Python^44^ programming language v.3.9.18.

### Classifier selection by a comparative performance of machine learning models

In the G2019S proteome dataset, we included 32 G2019S L2PD, 22 G2019S L2NMCs and 42 controls that passed the quality control criteria described above. Likewise, the phospho-proteome dataset comprised 29 G2019S L2PD, 19 G2019S L2NMCs and 35 controls. First, we assessed comparative model performances for each dataset considering an initial number of features, 3816 peptides and 10 180 phospho-peptides, respectively (Supplementary Table 1). Notably, in the proteome dataset, the SVM classifier demonstrated a substantial enhancement in balanced accuracy score following redundant feature elimination, achieving 0.91. This outcome indicates that the selective elimination of features contributed to obtaining a more discriminative model. On the contrary, the random forest classifier showed limited improvement, implying that feature elimination methods were less effective for this specific model. Consistently, we obtained similar results for the phospho-proteome dataset where, after feature elimination, SVM achieved a balanced accuracy of 0.95, again highlighting the efficacy of feature selection in enhancing model performance. Furthermore, gradient boosting demonstrated significant improvement with only 43 features. This result indicates that the model performance can be enhanced with only a small subset of features. After comparative evaluation and parameter optimization, we identified SVM as the optimal model to derive informative LRKK2 signatures using the minimum subset of relevant features that maximize the discrimination between classes.

### Identification of a differential G2019S phospho-/protein signature

After the SVM model selection, an initial set of relevant features was determined by incorporating only statistically significant features (*P* < 0.05) identified by the ANOVA test. Subsequently, we applied backwards recursive feature elimination with cross-validation,^45^ i.e. we eliminated features with relatively lower importance to reduce the number of features iteratively while maximizing the balanced accuracy score. To obtain the LRKK2 signature, we used the Monte Carlo tree search (MCTS)^46^ method. The MCTS strategy involved selecting the minimum combination of features that maximize the score in an additive manner. Considering that the combinatorial features scale rapidly, the depth of the tree was fixed to five to manage computational complexity. The reward at each tree node was computed as the balanced accuracy score obtained through model training with cross-validation, using the selected subset of features. At each iteration, the number of trees evaluated was set to 10 times the number of features. After evaluating all the trees, the MCTS identified the best feature to add, maximizing the reward. A stop node was introduced to halt the algorithm when no further improvement could be achieved. In summary, the procedure comprised: (i) selection of the first feature; (ii) MTCS evaluation of all possible trees and reward calculation; (iii) selection of the best feature to be added; (iv) iteration from the second step until the model stops; and (v) repetition from the first step until all features were screened. After the screening of all features, we selected the combinations of features with a balanced accuracy score of >0.90. The most prominently represented features were used as initial features for refinement by MCTS. Discriminant LRKK2 signatures were defined as the subset with the highest score after the refinement. Feature selection and refinement were implemented in Python v.3.9.18 using Scikit-learn v.1.3.1 and MCTS v.2.0.4 libraries (https://pypi.org/project/monte-carlo-tree-search).

### Phospho-/protein gene ontology enrichment

Differential phospho-/proteins gene ontology (GO) was assessed using Metascape^47^ cell component term using default settings (minimum overlap: 3; minimum enrichment: |1.5|; *P* < 0.05) and a Benjamini–Hochberg FDR multiple-testing adjusted *P* < 0.05. Specifically, for signature phospho-/proteins, we used a combination of cell component and biological processes, KEEGs, Reactome and wiki pathways under the same statistical significance cut-off.

### pSer106 RAB12 immunoblotting of 1-year follow-up PBMCs and MLi-2 LRRK2 inhibition assessment

Further details on pSer106 RAB12 validation by immunoblot in 1-year follow-up PBMC samples, in addition to the pSer106 RAB12 response to the LRRK2 MLi-2 inhibitor in freshly collected PBMCs, can be found in the Supplementary material.

### Clinical correlation of LRRK2 differential phospho-/proteins and disease severity

We performed a Spearman’s association analysis between the differential proteins and phospho-proteins across different comparisons (log_2_FC > |0.6|, adjusted *P* < 0.05) and UPDRS-III motor scores from PD patients and healthy controls. To this end, we used the ‘cor.test’ function from R (stats v.4.3.1) to calculate ρ coefficients and the EnhancedVolcano package (v.1.20.0) to represent correlation outputs. Statistical significance was set a Spearman’s correlation coefficient ρ > |0.5| and an FDR multiple-testing adjusted *P* < 0.05.

## Results

### G2019S proteome analyses show endolysosomal pathway deregulation

**Figure 1:**
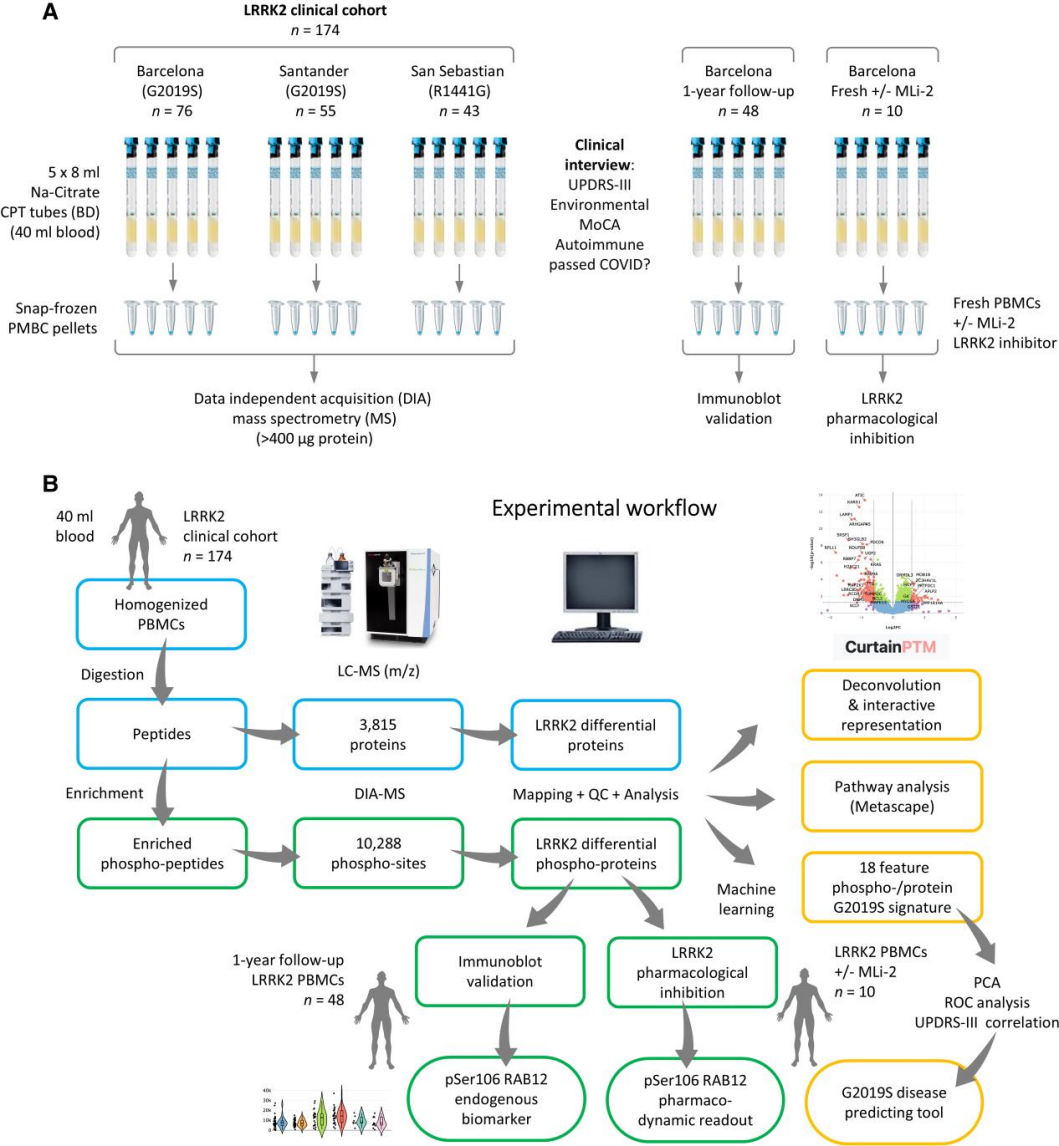
**Experimental workflow using peripheral blood mononuclear cells from a Spanish LRRK2 clinical cohort.** (**A**) Processing of peripheral blood mononuclear cells (PBMCs) for different applications. Blood samples (40 ml) were taken from individuals in an LRRK2 clinical cohort from Spain (*n* = 174), encompassing G2019S LRRK2-associated Parkinson’s disease patients (L2PD, *n* = 37), non-manifesting LRRK2 mutation carriers of G2019S (G2019S L2NMCs, *n* = 27), R1441G L2PD patients (*n* = 14), R1441G L2NMCs (*n* = 11), idiopathic PD (iPD, *n* = 40) and controls (*n* = 45). (**B**) After PBMC isolation, homogenization and protein digestion, data-independent acquisition-MS (DIA-MS) identified a total of 3815 proteins and 10 288 phospho-sites after phospho-enrichment. For the group differential analysis, we considered only proteins and phospho-sites mapped by at least two different peptides (Spetronaut) and with <30% imputation, with a significance cut-off of log_2_ fold-change > |0.6| and a false discovery rate multiple-testing adjusted *P* < 0.05. Data deconvolution and interactive representation of findings were done using the Curtain/Curtain PTM Tool, and gene ontology was assessed by Metascape. Using machine learning, we identified an 18-feature G2019S phospho-/protein signature able to discriminate G2019S L2PD, G2019S L2NMCs and controls. By immunoblot, we assessed pSer106 RAB12/total RAB12 levels in PBMCs from a subset of subjects (*n* = 48) after 1 year of follow-up, including G2019S L2PD (*n* = 12), G2019S L2NMCs (*n* = 6), iPD (*n* = 15) and controls (*n* = 15). Lastly, in freshly isolated PBMCs from a second subset of subjects (*n* = 10) encompassing G2019S L2PD (*n* = 3), R1441G L2PD (*n* = 1), iPD (*n* = 1) and healthy controls (*n* = 5) treated with dimethyl sulphoxide or the MLi-2 LRRK2 inhibitor, we performed an LRRK2 kinase assay measuring pSer106 RAB12/total RAB12 levels. COVID = coronavirus disease 2019; LC-MS = liquid chromatography mass spectrometry; MoCA = Montreal Cognitive Assessment; PCA = principal component analysis; QC = quality control; ROC = receiver operating characteristic curve; UPDRS III = Unified Parkinson's Disease Rating Scale III.

**Figure 2:**
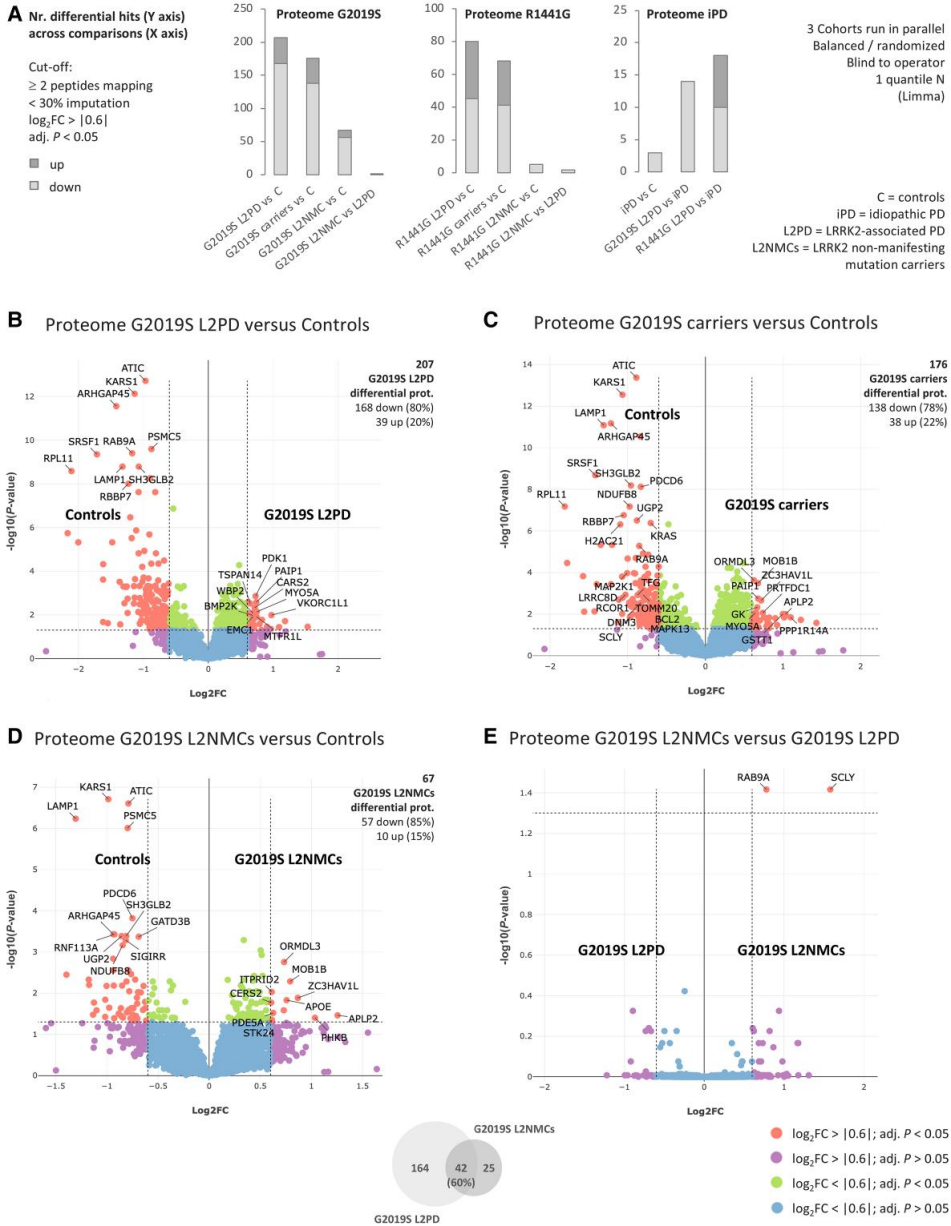
**Proteome overview and differential analyses in G2019S carriers.** (**A**) Bar plots showing the numbers of differential proteins in different pairwise comparisons involving G2019S carriers, R1441G carriers, idiopathic Parkinson's disease (iPD) and controls, with upregulated proteins in dark grey and downregulated proteins in light grey. All cohorts were run in parallel, with balanced study groups per run, blind to the operator, and using one quantile normalization (Limma). The significance cut-off was set at a log_2_ fold-change (FC) > |0.6| and a false discovery rate multiple-testing adjusted *P* < 0.05. (**B**) Volcano plot of the proteome differential analysis in G2019S LRRK2-associated PD (L2PD) patients versus healthy controls, with Curtain weblinks to access raw and differential analysis data, showing proteins upregulated in G2019S L2PD as red dots on the *right* and proteins upregulated in controls (i.e. downregulated in G2019S L2PD) as red dots on the *left* (Curtain). A colour code key applying to all panels is shown at the *bottom* of the figure, depicting statistically significant hits as red dots. (**C**) Volcano plot of the proteome differential analysis in G2019S carriers as a whole, i.e. L2PD and non-manifesting LRRK2 mutation carriers (L2NMCs), versus healthy controls (Curtain). (**D**) Volcano plot showing the proteome differential analysis between G2019S L2NMCs and healthy controls (Curtain). (**E**) Volcano plot representing the proteome comparison between G2019S L2NMCs and G2019S L2PD. A Venn diagram at the *bottom* of the figure shows the overlap of differential hits in PD-manifesting and non-manifesting G2019S carriers (Curtain). Curtain weblinks provide access to the differential analyses.

We succeeded in quantifying the levels of 3798 unique proteins using DIA-MS in our LRRK2 clinical cohort (Fig. 1). Pairwise analysis, under a cut-off of ≥2 peptide mapping, <0.30 imputation, log_2_FC > |0.60| and adjusted *P* < 0.05 revealed that G2019S L2PD was the most distinct group, displaying a set of 207 proteins whose levels differed versus controls, with 85% downregulated proteins (168 downregulated and 39 upregulated) (Fig. 2). Specifically, G2019S L2PD showed a number of proteins that had reduced expression, including: ATIC, which can repress LRRK2 and rescue neurodegeneration^48^ (log_2_FC = −0.97, adjusted *P* = 1.92 × 10^−13^); RAB9A, involved in phagocytic vesicle trafficking and lysosomal function (log_2_FC = −1.17, adjusted *P* = 3.97 × 10^−10^); and LAMP1, a lysosome biogenesis and autophagy regulator (log_2_FC = −1.32, adjusted *P* = 1.63 × 10^−9^). G2019S L2NMCs versus controls showed 67 differential hits, also involving 85% downregulated proteins (57 downregulated and 10 upregulated), which were mostly common and with the same FC direction as in G2019S L2PD (42 of 67), e.g. ATIC or LAMP1 (Supplementary Fig. 1). G2019S L2PD versus L2NMCs differed in only two proteins, which were downregulated in G2019S L2PD, i.e. RAB9A (log_2_FC = −0.77, adjusted *P* = 0.038) and SCLY, a selenocysteine lyase involved in peptide elongation (log_2_FC = −1.58, adjusted *P* = 0.038). These results indicate proteome changes associated with the G2019S mutation common to all G2019S carriers.

### Proteome pathway deficits of R1441G are similar to G2019S

Regarding the R1441G proteome, R1441G L2PD versus controls revealed 80 hits (45 downregulated and 35 upregulated) (Supplementary Fig. 2). Of these, 44% proteins (30 downregulated and 3 upregulated) overlapped with G2019S L2PD and had the same FC direction, including downregulation of NDUFB8, a mitochondrial Complex I subunit; PDCD6, a calcium sensor involved vesicle trafficking and apoptosis; RPL11, a component of the 60S ribosomal subunit; and other hits, such as ATIC, RAB9A, LAMP1 and SLCY. Likewise, R1441G L2NMCs versus controls showed five downregulated proteins, all common to R1441G L2PD, including NDUFB8 and PDCD6. Between R1441G L2PD and L2NMCs, two proteins were upregulated in R1441G L2PD, i.e. ATG3, an E2 ubiquitin-like conjugating enzyme, and MAGT2, which is essential for Golgi protein N-glycosylation. In addition, iPD versus controls, despite their larger sample, had only three differential hits, all downregulated and common to L2PD, i.e. SRSF1, an RNA splicing factor; UQCRB, a mitochondrial Complex III subunit; and LAMP1 (Supplementary Figs 1 and 4). Such findings can be related to the clinical heterogeneity of iPD, with diverse genetic and environmental aetiology. Functionally, proteome changes in G2019S and R1441G L2PD, even iPD, revealed a shared biological enrichment affecting endolysosomal trafficking, protein homeostasis (i.e. proteostasis) and mitochondrial function (Supplementary Fig. 3).

### LRRK2 phospho-proteome analyses uncovers elevated pSer106 RAB12 in G2019S carriers

**Figure 3:**
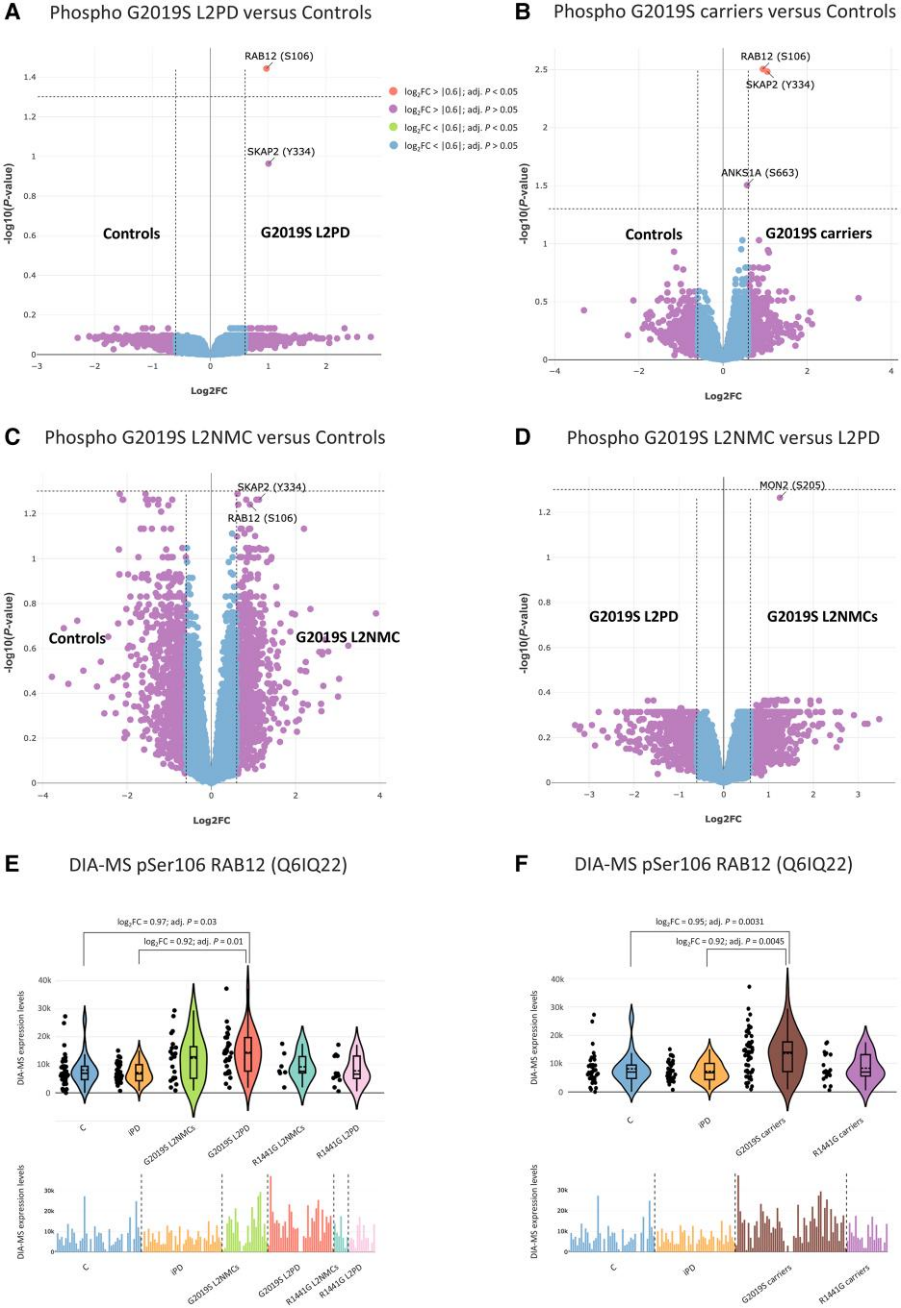
**Phospho-proteome differential analyses of G2019S carriers.** (**A**) Volcano plot of the phospho-proteome differential analysis of G2019S LRRK2-associated Parkinson's disease (L2PD) patients versus controls, and Curtain weblinks to raw and differential analysis data, representing hyperphosphorylated proteins in G2019S L2PD as red dots on the *right* with a single hit, elevated pSer106 RAB12 levels in G2019S L2PD, emerging as a differential phospho-peptide at a log_2_ fold-change (FC) > |0.6| and a false discovery rate multiple-testing adjusted *P* < 0.05 (Curtain PTM). A colour code key applying to all the panels shows categorization of hits by statistical significance. (**B**) Volcano plot showing phospho-protein hits in G2019S carriers as a whole, PD-manifesting and non-manifesting, compared with controls (Curtain PTM). (**C**) Phospho-proteome differences in G2019S non-manifesting LRRK2 mutation carriers (L2NMCs) versus controls (Curtain PTM). (**D**) Volcano showing phospho-proteome differences in G2019S L2NMCs versus G2019S L2PD (Curtain PTM). (**E**) Quality control crude non-imputed (bar plot, *bottom*), non-normalized (violin plot, *top*) MS data from pSer106 RAB12 levels across all study groups showing higher pSer106 phosphorylation levels in G2019S L2PD and G2019S L2NMCs with respect to the rest of the groups. The adjusted *P*-values and FC on *top* of the violin plot correspond to those from the differential analysis. (**F**) A similar analysis to **E**, with G2019S L2PD and G2019S L2NMCs grouped into a single group of G2019S carriers. Curtain weblinks provide access to the differential analyses.

Regarding the G2019S phospho-proteome, we found 10 288 phospho-sites mapping to 2657 proteins. Using the same stringent cut-off as above, G2019S L2PD versus controls displayed a single differential phospho-site, pSer106 RAB12, which was hyperphosphorylated in G2019S L2PD versus controls (log_2_FC = 0.97; adjusted *P* = 0.036) and in L2NMCs (log_2_FC = 0.92; adjusted *P* = 0.057) (Fig. 3). Remarkably, pSer106 RAB12 was shown to be a key physiological LRRK2 substrate of higher expression than other RABs, including pThr73 RAB10 in brain from PD models.^49,50^ G2019S carriers as a whole also showed elevated levels of pSer106 RAB12 (log_2_FC = 0.95; adjusted *P* = 0.003) along with pTyr334 SKAP2 (log_2_FC = 1.05; adjusted *P* = 0.003), a protein involved in the immune response of peripheral tissues that regulates neural functions in the CNS,^51^ including α-synuclein phosphorylation.^52^ G2019S L2PD versus L2NMCs showed downregulated levels of pSer205 MON2 (log_2_FC = 1.25; adjusted *P* = 0.05), a regulator of endosome-to-Golgi trafficking. Lastly, we found no differential hit in G2019S L2NMCs compared with controls. Collectively, these results identify elevated pSer106 RAB12 levels in a large clinical cohort of G2019S carriers, pinpointing, for the first time, pSer106 RAB12 as an endogenous biomarker in PBMCs from G2019S carriers.

### Phospho-proteome analyses in R1441G carriers and idiopathic Parkinson's disease

As for the R1441G phospho-proteome, R1441G L2PD versus controls showed no hit overpassing the multiple-testing adjustment of *P*-values. In addition, R1441G L2NMCs versus controls had 25 differential phospho-sites (20 downregulated and 5 upregulated), but none of these included pSer106 RAB12 (Supplementary Fig. 2). Moreover, R1441G carriers as a whole versus controls also showed no hit overpassing the statistical significance cut-off. Altogether, these findings indicate that enhancement of pSer106 RAB12 phosphorylation is a specific effect in G2019S PBMCs and suggest distinct phospho-signalling preferences occurring for different pathogenic LRRK2-activating mutations, such as G2019S and R1441G. Regarding iPD, at the phospho-proteome level, we found no phospho-peptide change versus controls (Supplementary Fig. 4). However, iPD revealed significant phospho-peptide differences compared with G2019S L2PD (84 downregulated and 9 upregulated), including pSer106 RAB12, whose levels were elevated in G2019S L2PD, and also in R1441G L2PD (409 downregulated and 225 upregulated). Altogether, these findings indicate that phospho-protein derangements are more prominent in L2PD owing to phospho-signalling effects of LRRK2-activating mutations than in iPD, with pSer106 RAB12 being a preferred LRRK2 substrate in PBMCs from G2019S carriers.

### pSer106 RAB12 immunoblot validation and LRRK2 inhibition assessment

**Figure 4:**
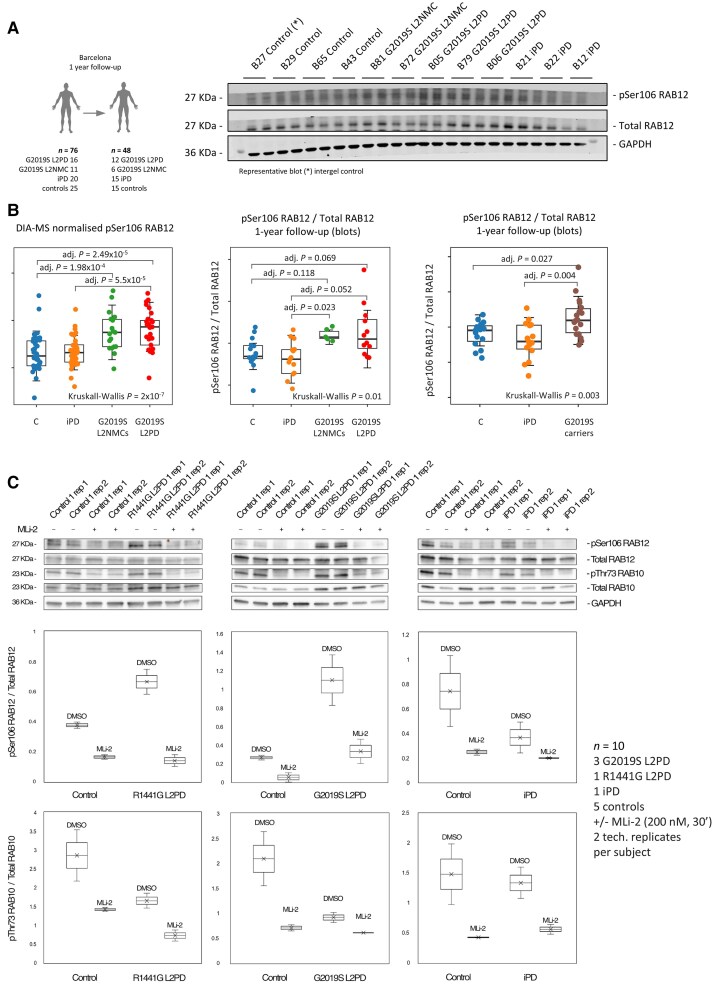
**One-year follow-up of pSer106 RAB12 by immunoblot and MLi-2 response.** Immunoblot assessment of pSer106 RAB12 phosphorylation levels in >1-year follow-up peripheral blood mononuclear cell (PBMC) samples from part of the LRRK2 subcohort from Clínic-Barcelona (*n* = 48), including G2019S LRRK2-associated Parkinson's disease (L2PD, *n* = 12), G2019S non-manifesting LRRK2 mutation carriers (L2NMCs, *n* = 6), idiopathic PD (iPD, *n* = 15) and controls (*n* = 15). (**A**) Schematic workflow of immunoblot assessment and representative blot from five different blots shown in the Supplementary material. *Intergel control. (**B**) Dot plots comparing pSer106 RAB12/total RAB12 levels obtained by data-independent acquisition-MS (DIA-MS) at the entire LRRK2 clinical cohort (*n* = 174) on the *left* and by immunoblot of part of the Clínic-Barcelona cohort after 1-year of follow-up (*n* = 48) in G2019S carriers on the *right*. In each plot, overall intergroup differences were assessed using the Kruskal–Wallis test followed by Dunn’s *post hoc* test to evaluate for pSer106 RAB12/total RAB12 differences in G2019S carriers. (**C**) Representative immunoblot analysis of pSer106 RAB12/total RAB12 and pThr73 RAB10/Total RAB10 using technical replicates from additional freshly collected PBMCs from one R1441G L2PD, one G2019S L2PD, one iPD and three controls (expanded to a total *n* = 10 subjects in the Supplementary material), treated with dimethyl sulphoxide or the MLi-2 LRRK2 inhibitor (200 nM, 30 min), showing a diminishment of pSer106 RAB12 phosphorylation levels after LRRK2 inhibition by MLi-2 treatment.

By immunoblot, we assessed pSer106 RAB12 levels as a pSer106 RAB12/total RAB12 ratio using >1-year follow-up PBMC samples from the G2019S cohort recruited at Clínic-Barcelona (*n* = 48). These encompassed G2019S L2PD (*n* = 12), G2019S L2NMCs (*n* = 6), iPD (*n* = 15) and healthy controls (*n* = 15) (Table 1). Consistent with DIA-MS data, we found differences in phosphorylation across the different groups (Kruskal–Wallis *P* = 0.01), with increased pSer106 RAB12 phosphorylation levels in G2019S L2PD (Dunn’s adjusted *P* = 0.069) and L2NMCs (Dunn’s adjusted *P* = 0.118) versus controls. Likewise, G2019S carriers as a whole also showed elevated pSer106 RAB12 levels compared with controls (Kruskal–Wallis *P* = 0.003; Dunn’s adjusted *P* = 0.027), but not in iPD (Fig. 4 and Supplementary Fig. 5). However, by immunoblot^53^ we did not observe downregulation of proteome hits, such as RAB9A in G2019S L2PD or iPD (Kruskal–Wallis *P* = 0.08) nor LAMP1 except in iPD (Kruskal–Wallis *P* = 0.03; Dunn’s adjusted *P* = 0.046). Lastly, we assessed the pSer106 RAB12 response to LRRK2 pharmacological inhibition by MLi-2 using technical replicates from freshly collected PBMC pellets from an additional set of probands (*n* = 10), including three G2019S L2PD, one R1441G L2PD, one iPD and five controls treated with MLi-2 (200 nM; 30 min) or dimethyl sulphoxide (Supplementary Fig. 6). In all subjects, we observed a substantial diminishment of pSer106 RAB12 phosphorylation levels after MLi-2 treatment, confirming pSer106 RAB12 as a pharmacodynamic readout of LRRK2 inhibition in PBMCs.

### Phospho-/protein signatures define Parkinson's disease manifesting and non-manifesting G2019S carriers

**Figure 5:**
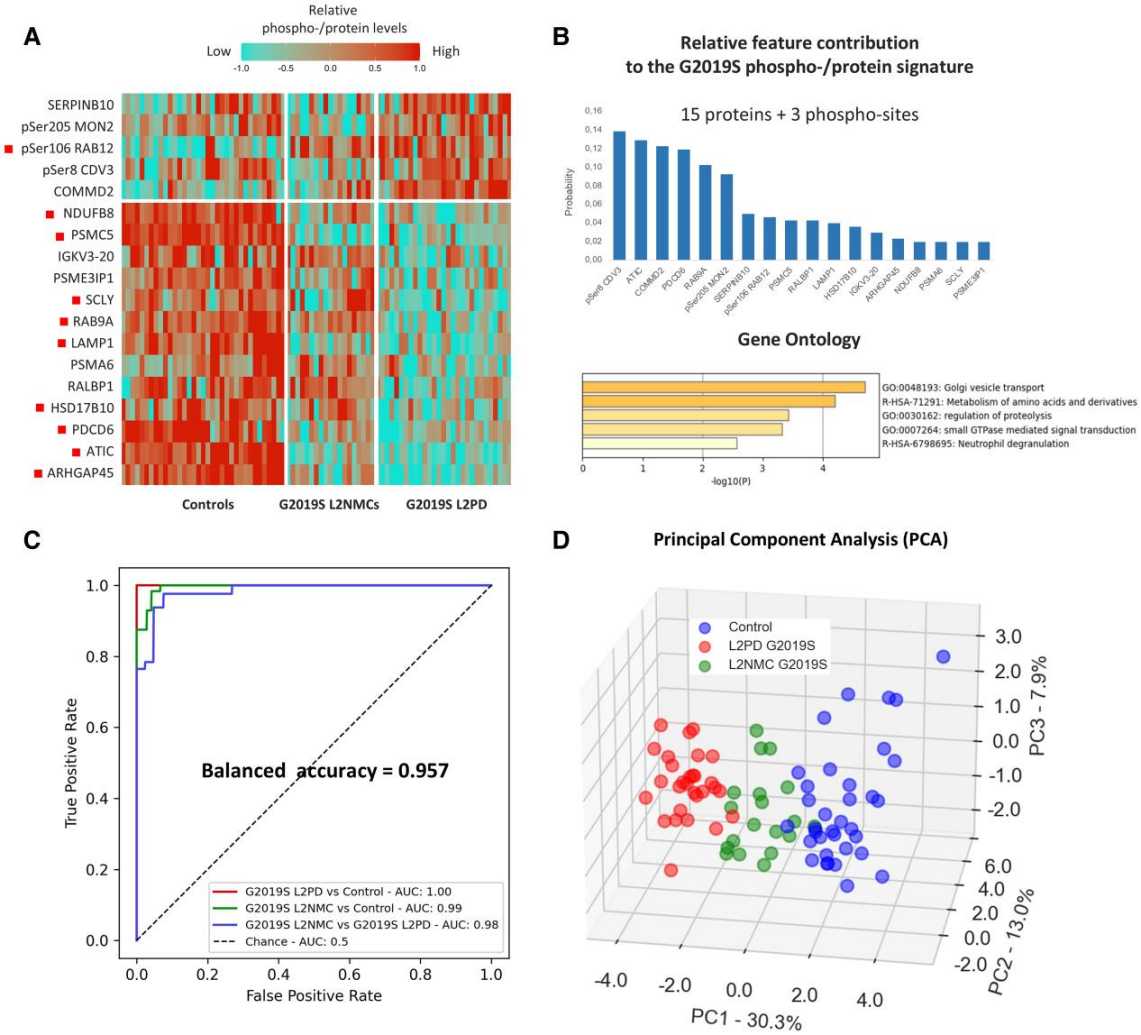
**Identification of an 18-feature phospho-/protein classifier for G2019S carriers.** After comparing the performance of several models, we applied supported vector machine (SVM) learning, adjusted by unbalanced groups using the synthetic minority over-sampling technique (SMOTE), corrected from overfitting with 5-fold cross-validation, identified cross-group differential proteins and phospho-proteins by ANOVA and recursive feature elimination with cross-validation, and refined informative combinations to the minimal numbers of features yielding the maximal balanced accuracy by the Monte Carlo tree search (MCTS) method. (**A**) Eighteen-feature G2019S phospho-/protein best classifier identified in G2019S carriers, Parkinson's disease (PD)-manifesting and non-manifesting subjects and healthy controls. Red dots indicate individual features correlating with disease severity (UPDRS-III) (see Fig. 6). (**B**) Relative contribution of the different proteins (*n* = 15) and phospho-sites (*n* = 3), including pSer106 RAB12, from the 18-feature G2019S classifier on the *top* bar plot; Metascape gene ontology enrichment analysis of the 18-features G2019S signature *bottom* bar plot. (**C**) Receiver operating curve analysis of the 18-feature G2019S phospho-/protein signature showing an overall balanced accuracy of 0.957 to discriminate G2019S LRRK2-associated PD (L2PD), G2019S non-manifesting LRRK2 mutation carriers (L2NMCs) and controls, specifically with an area under the curve of 1.00 between G2019S L2PD and controls, 0.99 between G2019S L2NMCs and controls, and 0.98 between G2019S L2PD and G2019S L2NMCs. (**D**) Principal component analysis based on the 18-feature G2019S phospho-/protein classifier in G2019S carriers and healthy controls, showing distinct group profiles based on LRRK2 mutation and disease status, with G2019S L2NMCs in between G2019S L2PD and controls, consistent with their disease status. UPDRS III = Unified Parkinson's Disease Rating Scale III.

Next, we interrogated G2019S phospho-/protein signatures. We applied an SVM classifier, adjusting for unbalanced group sizes, using 5-fold cross-validations as overfitting control. After recursive feature elimination, we obtained 510 peptides and 204 phospho-proteins as multi-class informative items. By MCTS, we refined combinations to the minimal numbers of features yielding the maximal balanced accuracy. We identified an 18-feature signature of 15 proteins and three phospho-proteins (Fig. 5 and Supplementary Fig. 7), including pSer106 RAB12 and pSer205 MON2, ATIC, RAB9A, LAMP1, NDUFB8 and SCLY, which yielded a balanced accuracy of 96% to discriminate G2019S carrier groups and controls. Specifically, receiver operating curve analysis revealed an area under the curve of 1.00 for G2019S L2PD versus controls, 0.99 for G2019S L2NMCs versus controls, and 0.98 between G2019S L2PD and L2NMCs. The top gene ontology term of the 18 features was vesicle transport, thus supporting a biological plausibility. Altogether, the 18-feature phospho-/protein signature correctly classified 96% of G2019S L2PD, G2019S L2NMCs and healthy controls, thus holding potential to assess disease progression.

### Differential phospho-/proteins are correlated with disease severity

**Figure 6:**
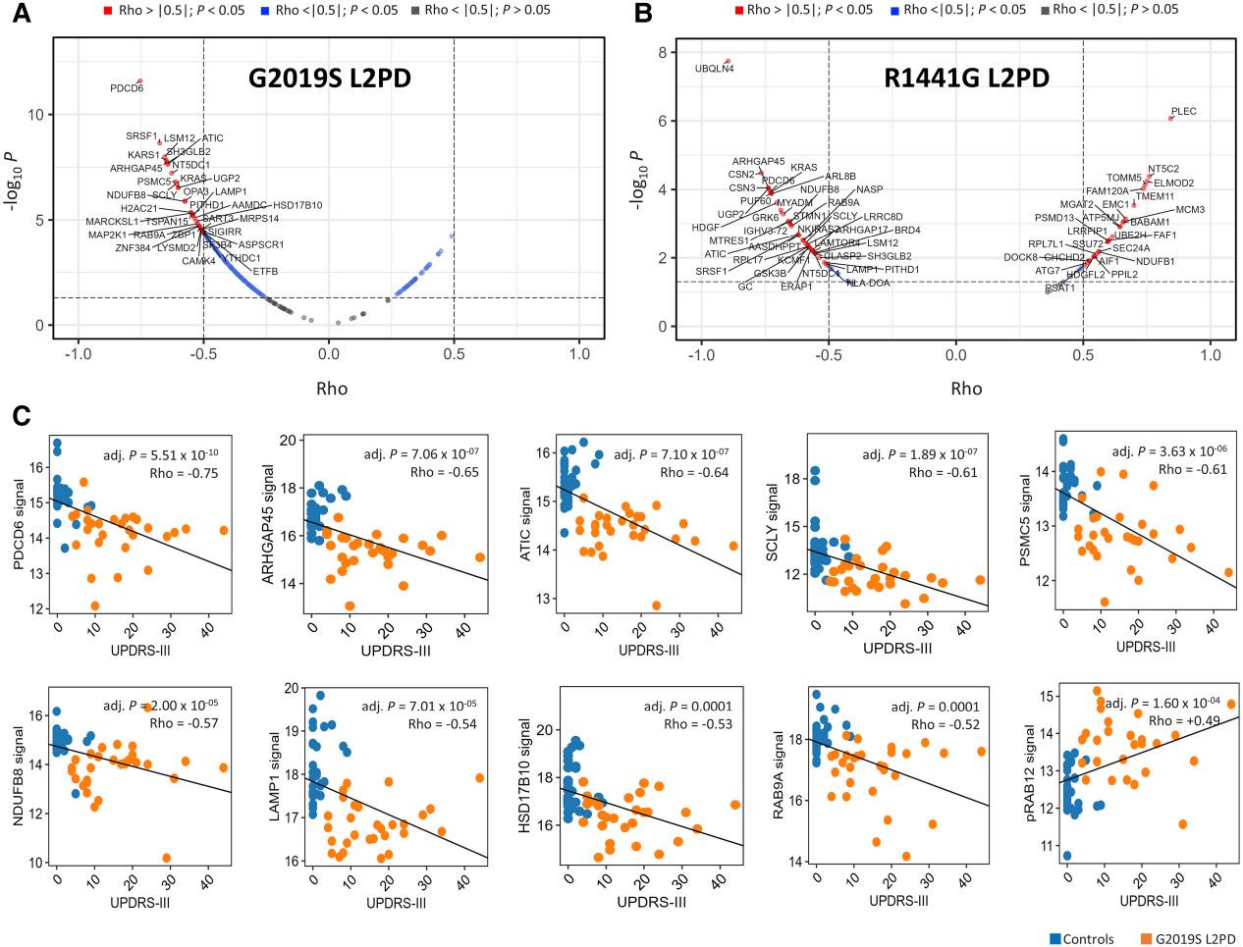
**Association between differential LRRK2 phospho-/proteins and disease severity.** Correlation analysis of differential proteins and phospho-proteins [log_2_ fold-change (FC) > |0.6|, adjusted *P* < 0.05] and UPDRS-III motor scores from LRRK2-associated Parkinson's disease (L2PD) patients and healthy controls with statistical significance set at a Spearman’s correlation coefficient ρ > |0.5| and a false discovery rate multiple-testing adjusted *P* < 0.05. (**A** and **B**) Correlation plots between differential proteins in G2019S L2PD versus controls (**A**) and R1441G L2PD versus controls (**B**) showing differential hits correlating with UPDRS-III in red. (**C**) Scatter plots of 10 hits from the 18-feature G2019S phospho-/protein signature correlating with UPDRS-III in G2019S L2PD patients represented as orange dots and healthy controls as blue dots, including PDCD6, ARHGAP45, ATIC, SCLY, PSMC5, NDUFB8, LAMP1, HSD17B10, RAB9A and pSer106 RAB12. UPDRS III = Unified Parkinson's Disease Rating Scale III.

Lastly, we assessed the relationship between deregulated phospho-/proteins and disease severity. Under a Spearman’s ρ > |0.5| and a *P* < 0.05, 16% of the differential proteins between G2019S L2PD versus controls (34 of 207) had an inverse association with UPDRS-III motor scores, whereas pSer106 RAB12 and pSer205 MON2 had a direct correlation. Moreover, 55% of the 18 features at the G2019S phospho-/protein signature were correlated inversely with UPDRS-III (ATIC, PDCD6, RAB9A, PSMC5, LAMP1, HSD13B10, ARHGAP45, NDUFB8 and SCLY), whereas pSer106 RAB12 was correlated positively (ρ = 0.49, adjusted *P* = 1.60 × 10^−4^), i.e. the higher the pSer106 RAB12 levels, the higher the UPDRS-III scores (Fig. 6). In R1441G L2PD versus controls, 81% of the differential proteins (65 of 80) were correlated with UPDRS-III, either inversely (59%) or positively (41%). Remarkably, several of these R1441G proteins are part of the 18-feature G2019S signature (PDCD6, ARHGAP45, NDUFB8, RAB9A, ATIC, SCLY and LAMP1), whereas other proteins were specific for R1441G, e.g. the mitochondrial UBQLN4 (ρ = −0.89, *P* = 1.64 × 10^−6^) or the cytoskeletal PLEC (ρ = 0.84, *P* = 3.50 × 10^−5^) proteins. As an example, PDCD6, a top common correlating protein between G2019S and R1441G (ρ = −0.75, *P* = 5.51 × 10^−10^), participates in vesicle trafficking, mediates mitochondrial cytochrome *c* release and apoptosis,^54^ and has been linked to PD.^55^ In summary, although correlation does not mean causality, differential phospho-/proteins at the 18-feature G2019S classifier are associated with disease severity, therefore holding clinical interest.

## Discussion

Following FAIR principles,^27^ we used an interactive tool called Curtain,^28^ in which the raw and differential phospho-/proteomic data from all analyses are saved as weblinks that non-MS experts can readily explore for public data mining. Overall, the G2019S L2PD proteome showed the highest number of changes, 207 proteins, most of which were downregulated (85%). The G2019S L2NMCs displayed fewer protein differences, 67, which were also mostly downregulated (85%). There was a substantial overlap between proteins that changed in both groups (60%). The comparison between G2019S L2PD and L2NMCs revealed two proteins, RAB9A and SCLY, which were downregulated in the symptomatic carriers. Our findings indicate prominent protein deficits associated with *LRRK2* pathogenic mutations, such as G2019S, which begin at G2019S L2NMCs premotor stages^56^ and progress to G2019S L2PD stages.

### Endolysosomal and proteostasis defects in G2019S LRRK2 blood

Gene ontology analysis annotated the protein changes in the G2019S carriers as participating in the endolysosomal pathway, i.e. vesicle trafficking. For example, G2019S L2PD showed downregulation of RAB9A, which controls phagocytosis and lysosomal biology.^57,58^ In G2019S carriers, we also observed downregulated levels of LAMP1, a canonical lysosomal marker involved in lysosome biogenesis, which supports an enhanced LRRK2 activity in G2019S carriers.^7-9^ A previous study also noted that LAMP1 levels were reduced in CSF of L2PD.^59^ Our findings are consistent with the current understanding of the LRRK2 pathway, indicating that the LRRK2 protein plays a crucial role in controlling the endolysosomal pathway.^50,60^ Beyond that, we also observed protein changes related to G2019S affecting ribosomal function, protein homeostasis, mitochondrial function and alternative splicing.^61^ For instance, ATIC, the top protein downregulated in G2019S carriers, catalyses the last two steps of mitochondria purine biosynthesis.^62,63^ Another study has also linked ATIC to LRRK2 toxicity.^48^ Other protein deficits included KARS1, a transfer RNA synthetase; PSMC5, the proteasomal 26S subunit; and SCLY, selenocysteine lyase, an enzyme involved in peptide elongation that has been related to neurodegeneration.^64^ These findings align with studies reporting transcriptional repression of proteostasis regulators in G2019S L2PD^65^ and proteostasis defects in PD substantia nigra.^66^

### Similar proteomic deficits in R1441G carriers to those in G2019S

R1441G L2PD showed 80 differential proteins, 44% of which were shared with G2019S L2PD. Enrichment analysis showed that the functions of the proteins deregulated in R1441G carriers also affected the endolysosomal pathway, protein homeostasis and mitochondrial function. Indeed, the R1441G L2PD top downregulated protein, NDUFB8, is a subunit of the mitochondrial Complex I (NADH to ubiquinone oxidoreductase), the activity of which is deficient in PD.^67^ In addition, R1441G L2PD and L2NMCs displayed few protein differences, notably ATG3, which is involved in autophagy, and MGAT2, a Golgi glycosyl transferase. In summary, the proteomic effects of the R1441G mutation in our LRRK2 clinical cohort were largely similar to G2019S.^68,69^ Furthermore, the iPD proteome, despite being the largest group, displayed only three differential proteins, which were commonly decreased in G2019S L2PD, R1441G L2PD and iPD. These encompassed LAMP1, which further supports endolysosomal dysfunction occurring in iPD^60,70^; SRSF1, a serine/arginine-rich splicing factor; and UQCRB, a mitochondrial Complex III subunit (ubiquinol–cytochrome *c* oxidoreductase). Beyond the aetiopathological heterogeneity of iPD,^71,72^ the fewer protein changes detected in iPD than in G2019S and R1441G L2PD indicate stronger signal transduction derangements attributable to pathogenic *LRRK2* mutations in L2PD than in iPD. However, proteome changes in G2019S and R1441G L2PD, even iPD, affected the same biological processes in a similar manner.

### pSer106 RAB12 as an endogenous G2019S biomarker

At the phospho-proteome level, a single hit, pSer106 RAB12, was elevated specifically in G2019S carriers but not in R1441G. Excitingly, this phospho-site comprises a critical physiological substrate of LRRK2.^2^ Overall, the roles that RAB12 plays and its phosphorylation by LRRK2 are poorly understood. Phosphorylation of RAB12 is prominent in the brain in PD models and observed to be higher than other RAB substrates, such as RAB10, in this organ.^49,50^ Functionally, other studies showed that RAB12 is located in phagosomes, lysosomes and late endosomes, where it might regulate endosome-to-trans-Golgi trafficking and exocytosis.^73,74^ Ours is the first report of hyperphosphorylated RAB12 in PBMCs from a large clinical cohort of G2019S carriers. In addition, we analysed *n* = 48 follow-up PBMC samples after 1 year of follow-up by immunoblotting. Despite the smaller sample than for DIA-MS, we found an increase of pSer106 RAB12 in G2019S L2PD and L2NMCs. Previous studies in neutrophils probing for RAB10 but not RAB12 phosphorylation revealed elevated pThr73 RAB10 in R1441G but not G2019S carriers.^19^ In our study, by DIA-MS, pThr73 RAB10 did not pass the quality control cut-offs in all three cohorts, only in PBMCs from Barcelona (*n* = 76), which did not show differences in pRAB10 between G2019S L2PD and controls (log_2_FC = 0.71, adjusted *P* = 0.999) or between G2019S carriers and controls (log_2_FC = 0.68, adjusted *P* = 0.999). These results in G2019S PBMCs suggest either RAB12 that is a preferred substrate for LRRK2 (indeed, distinct mutation effects cannot be ruled out^75^) or that pThr73 RAB10 phosphatases, e.g. PPM1H,^76^ could dephosphorylate RAB10 more efficiently than RAB12. Mechanistic studies on how G2019S and other *LRRK2* variants preferentially phosphorylate different RABs in various cell types and using larger cohorts are warranted. Our study identifies pSer106 RAB12 as an endogenous biomarker in easily accessible PBMCs from carriers of the most prevalent G2019S mutation, either PD-manifesting or non-manifesting, suggesting that pSer106 RAB12 can be used as a marker of LRRK2 activity in G2019S carriers.

### pSer106 RAB12 as a marker of LRRK2 activity

Upstream of LRRK2, PD cell models showed LRRK2 activation by VPS35/RAB29 (RAB7L1) binding to a region on the Armadillo (ARM) domain termed ‘Site-1’.^22,23^ More recently, non-phosphorylated RAB12 was shown to be a key LRRK2 activator that binds to a distinct site at the ARM domain termed ‘Site-3’.^24,25^ One study showed that RAB12 plays a role in recruiting LRRK2 to damaged or stressed lysosomes.^25^ These studies suggested that ARM domain Site-1 or Site-3 inhibitors that block RAB binding could serve as novel therapeutic targets for allosteric inhibitors of LRRK2 kinase activity.^24^ The biological effect of pSer106 RAB12 phosphorylation on LRRK2 regulation has not been well characterized, and our results in G20919S carriers emphasize that additional work is warranted to investigate this. Specifically, it is key to investigate whether pSer106 RAB12 binding to the ARM Site-3 can create feedback loops modulating LRRK2 activity through activation or inhibition depending on the cellular context and, importantly, how this translates to LRRK2 clinical cohorts. Downstream of LRRK2, MLi-2 phospho-proteomics identified RAB3A, RAB8A, RAB10, RAB12, RAB29 and RAB43 as LRRK2 substrates.^2,3,20,77^ In line with these studies, using freshly collected PBMCs (*n* = 10), we found that pSer106 RAB12 levels strongly diminished after MLi-2 LRRK2 inhibition in all subjects, regardless of disease or mutation status. In the clinical setting, only pThr73 RAB10 has been validated as an LRRK2 substrate^19^ and exploited as a readout of LRRK2 activity in previous studies,^18^ including in LRRK2 inhibitor clinical trials.^12,21^ As mentioned above, there has not yet been a specific way of assessing elevated LRRK2 activity in G2019S carriers, owing to the lack of effect in pThr73 RAB10 phosphorylation. Thus, monitoring pSer106 RAB12 phosphorylation levels could be useful for assessing G2019S selective inhibitors that have been newly developed in clinical studies,^78-81^ because these would be expected preferentially to reduce pSer106 RAB12 phosphorylation in patients with heterozygous G2019S mutations.

### Dual role of RAB12 upstream and downstream of LRRK2 signalling

RAB12 was shown to play a dual role in both downstream and upstream signalling of LRRK2. Current evidence points to a mechanism by which lysosomal stress and dysfunction lead to the GTP loading and activation of dephosphorylated RAB12 at the lysosome membrane. This, in turn, recruits LRRK2 to the lysosome, where RAB12 interacts directly with Site-3 on the ARM domain of LRRK2.^82^ LRRK2 is then activated at the lysosomal membrane, although the exact mechanism remains poorly understood. Once activated, LRRK2 phosphorylates nearby RAB proteins, including RAB12 at Ser106. In this context, our data suggest that monitoring pSer106 RAB12 phosphorylation could be a relevant biomarker for tracking LRRK2 activation, particularly in LRRK2 G2019S PBMCs. Further research is needed to understand fully the biological roles of phosphorylated RAB12 and to identify the proteins and downstream pathways it regulates.

### G2019S phospho-/protein signatures can reflect disease progression

In G2019S carriers, we identified a signature of 15 proteins and three phospho-sites, including pSer106 RAB12, which was found to provide a 96% accuracy in discriminating G2019S L2PD, L2NMCs and controls. Although correlation does not imply causality, 55% of the signature features were correlated with PD motor severity as determined by UPDRS-III scores, including pSer106 RAB12, pSer2015 MON2, ATIC, PDCD6, RAB9A, PSMC5, LAMP1, HSD13B10, ARHGAP45, NDUFB8 and SCLY. These results suggest that this phospho-signature can be related to PD progression. However, further work in larger LRRK2 clinical cohorts would be required to assess this clinically.^56^ Altogether, as a proof of principle, we identified the first phospho-/protein signature in G2019S PBMCs based on DIA-MS data, which complements previous G2019S signatures reported in blood^83^ and urine.^84,85^

### Study limitations

Despite the exciting findings, our study has limitations. Inherent variation in humans markedly affects differential protein expression and phosphorylation. Slightly different preparation and storage procedures for PBMCs at different centres can also affect the results. To minimize this variation, we undertook DIA-MS analyses simultaneously for all subcohorts and blinded to study groups. Differential enrichment of phospho-peptides on titanium dioxide beads can result in further variety. Indeed, we discarded one of the phospho-peptide batches because it did not pass quality control. Based on our phospho-peptide enrichment approach, the detection of phospho-tyrosines was under-represented. We used stringent significance cut-off criteria, filtering in only hits mapped by at least two peptides, and we cannot rule out the possibility that other important proteins have been excluded. The number of R1441G carriers, especially L2NMCs, was smaller than G2019S, and it was limited for phospho analyses and insufficient to assess signatures by machine learning. Nonetheless, pSer106 RAB12 did not show significant differences or trends in R1441G groups. The validation of pRAB12 by immunoblot in 1-year follow-up clinical samples strengthens the robustness of this as an endogenous biomarker for G2019S carriers. Lastly, other phospho-/protein candidates identified by DIA-MS should be validated in additional studies.

## Conclusion

Aligning with urine,^85^ in PBMCs, we found elevated pSer106 RAB12 levels to be an endogenous biomarker for G2019S carriers. This finding has clinical implications, suggesting that pSer106 RAB12 can be a marker of LRRK2 activity in G2019S carriers. Other studies should also assess pSer106 RAB12 levels in CSF and brain tissue of LRRK2 patients carrying the G2019S mutation. In addition, given that RAB12 was shown to be a key LRRK2 activator in PD models able to increase pThr73 RAB10 levels,^24,25^ future studies ought to investigate the effect of pSer106 RAB12 phosphorylation on LRRK2 activation. Moreover, in line with findings from PD models,^50,86^ in human LRRK2 PBMCs we also found that pSer106 RAB12 represents a pharmacodynamic readout of LRRK2 inhibition. In addition, we found an 18-feature signature, including pSer106 RAB12, with a 96% accuracy in discriminating symptomatic and asymptomatic G2019S carriers and controls. Future large-scale studies need to assess pSer106 RAB12 in other G2019S clinical cohorts. Moreover, developing novel assays able to quantify pSer106 RAB12 in LRRK2 clinical samples such as blood cells and CSF, e.g. reaction monitoring or ELISA-based assays, are needed to translate our findings to clinical settings. If validated, pSer106 RAB12 can aid patient enrichment and target engagement in clinical trials of novel LRRK2 inhibitors targeting the G2019S mutation.^78-81^

## Supplementary Material

awae404_Supplementary_Data

## Data Availability

The mass spectrometry proteomics data have been deposited to the ProteomeXchange Consortium via the PRIDE^87^ partner repository, with the dataset identifiers PXD050865 for the proteome and PXD050944 phospho-proteome analyses. Following FAIR principles,^27^ through the interactive tool called Curtain,^28^ raw and differential phospho-/proteomic data from all comparisons are also provided as weblinks to be explored readily by non-MS experts. Programming R scripts for data analyses are publicly available at *Brain* online (Supplementary material) and as a cloud weblink (doi:10.5281/zenodo.13774022).

## Web resources

Curtain weblinks of LRRK2 phospho-/proteomic analyses as presented in the article. Expanded analyses can be found at *Brain* online (Supplementary material).

Proteome G2019S cohort: G2019S L2PD versus controls; G2019S carriers versus controls; G2019S L2NMCs versus controls; G2019S L2NMCs versus L2PD; iPD versus controls; G2019S L2PD versus iPD; G2019S carriers versus iPD.

Proteome R1441G cohort: R1441G L2PD versus controls; R1441G carriers versus controls; R1441G L2NMCs versus controls; R1441G L2NMCs versus L2PD; iPD versus controls; R1441G L2PD versus iPD; R1441G carriers versus iPD.

Phospho G2019S cohort: G2019S L2PD versus controls; G2019S carriers versus controls; G2019S L2NMCs versus controls; G2019S L2NMCs versus L2PD; iPD versus controls; G2019S L2PD versus iPD; G2019S carriers versus iPD.

Phospho R1441G cohort: R1441G L2PD versus controls; R1441G carriers versus controls; R1441G L2NMCs versus controls; R1441G L2NMCs versus L2PD; iPD versus controls; R1441G L2PD versus iPD; R1441G carriers versus iPD.

## References

[1] Sheng Z, Zhang S, Bustos D, et al Ser1292 autophosphorylation is an indicator of LRRK2 kinase activity and contributes to the cellular effects of PD mutations. Sci Transl Med. 2012;4:164ra161. 10.1126/scitranslmed.3004485 23241745

[2] Steger M, Tonelli F, Ito G, et al Phosphoproteomics reveals that Parkinson’s disease kinase LRRK2 regulates a subset of Rab GTPases. Elife. 2016;5:e12813. 26824392 10.7554/eLife.12813 PMC4769169

[3] Steger M, Diez F, Dhekne H, et al Systematic proteomic analysis of LRRK2-mediated rab GTPase phosphorylation establishes a connection to ciliogenesis. Elife. 2017;6:e31012. 29125462 10.7554/eLife.31012 PMC5695910

[4] Taylor M, Alessi DR. Advances in elucidating the function of leucine-rich repeat protein kinase-2 in normal cells and Parkinson’s disease. Curr Opin Cell Biol. 2020;63:102–113. 32036294 10.1016/j.ceb.2020.01.001 PMC7262585

[5] Zimprich A, Biskup S, Leitner P, et al Mutations in *LRRK2* cause autosomal-dominant parkinsonism with pleomorphic pathology. Neuron. 2004;44:601–607. 15541309 10.1016/j.neuron.2004.11.005

[6] Paisán-Ruíz C, Jain S, Evans EW, et al Cloning of the gene containing mutations that cause *PARK8*-linked Parkinson’s disease. Neuron. 2004;44:595–600. 15541308 10.1016/j.neuron.2004.10.023

[7] Di Maio R, Hoffman EK, Rocha EM, et al LRRK2 activation in idiopathic Parkinson’s disease. Sci Transl Med. 2018;10:eaar5429. 30045977 10.1126/scitranslmed.aar5429 PMC6344941

[8] Fraser KB, Rawlins AB, Clark RG, et al Ser(P)-1292 LRRK2 in urinary exosomes is elevated in idiopathic Parkinson’s disease. Mov Disord. 2016;31:1543–1550. 27297049 10.1002/mds.26686 PMC5053851

[9] Wang X, Negrou E, Maloney MT, et al Understanding LRRK2 kinase activity in preclinical models and human subjects through quantitative analysis of LRRK2 and pT73 Rab10. Sci Rep. 2021;11:12900. 34145320 10.1038/s41598-021-91943-4 PMC8213766

[10] Healy DG, Falchi M, O’Sullivan SS, et al Phenotype, genotype, and worldwide genetic penetrance of *LRRK2*-associated Parkinson’s disease: A case-control study. Lancet Neurol. 2008;7:583–590. 18539534 10.1016/S1474-4422(08)70117-0 PMC2832754

[11] Marras C, Alcalay RN, Caspell-Garcia C, et al Motor and nonmotor heterogeneity of *LRRK2*-related and idiopathic Parkinson’s disease. Mov Disord. 2016;31:1192–1202. 27091104 10.1002/mds.26614

[12] Jennings D, Huntwork-Rodriguez S, Vissers MFJM, et al LRRK2 inhibition by BIIB122 in healthy participants and patients with Parkinson’s disease. Mov Disord. 2023;38:386–398. 36807624 10.1002/mds.29297

[13] Tolosa E, Vila M, Klein C, Rascol O. LRRK2 in Parkinson disease: Challenges of clinical trials. Nat Rev Neurol. 2020;16:97–107. 31980808 10.1038/s41582-019-0301-2

[14] Hentati F, Trinh J, Thompson C, Nosova E, Farrer MJ, Aasly JO. *LRRK2* parkinsonism in Tunisia and Norway: A comparative analysis of disease penetrance. Neurology. 2014;83:568–569. 25008396 10.1212/WNL.0000000000000675 PMC4142000

[15] Marder K, Wang Y, Alcalay RN, et al Age-specific penetrance of *LRRK2* G2019S in the Michael J. Fox Ashkenazi Jewish LRRK2 Consortium. Neurology. 2015;85:89–95. 26062626 10.1212/WNL.0000000000001708 PMC4501942

[16] Lee AJ, Wang Y, Alcalay RN, et al Penetrance estimate of *LRRK2* p.G2019S mutation in individuals of non-Ashkenazi Jewish ancestry. Mov Disord. 2017;32:1432–1438. 28639421 10.1002/mds.27059 PMC5656509

[17] Homma Y, Hiragi S, Fukuda M. Rab family of small GTPases: An updated view on their regulation and functions. FEBS J. 2021;288:36–55. 32542850 10.1111/febs.15453 PMC7818423

[18] Karayel Ö, Tonelli F, Virreira Winter S, et al Accurate MS-based Rab10 phosphorylation stoichiometry determination as readout for LRRK2 activity in Parkinson’s disease. Mol Cell Proteomics. 2020;19:1546–1560. 32601174 10.1074/mcp.RA120.002055 PMC8143643

[19] Fan Y, Nirujogi RS, Garrido A, et al R1441g but not G2019S mutation enhances LRRK2 mediated Rab10 phosphorylation in human peripheral blood neutrophils. Acta Neuropathol. 2021;142:475–494. 34125248 10.1007/s00401-021-02325-z PMC8357670

[20] Thirstrup K, Dächsel JC, Oppermann FS, et al Selective LRRK2 kinase inhibition reduces phosphorylation of endogenous Rab10 and Rab12 in human peripheral mononuclear blood cells. Sci Rep. 2017;7:10300. 28860483 10.1038/s41598-017-10501-z PMC5578959

[21] Jennings D, Huntwork-Rodriguez S, Henry AG, et al Preclinical and clinical evaluation of the LRRK2 inhibitor DNL201 for Parkinson’s disease. Sci Transl Med. 2022;14:eabj2658. 35675433 10.1126/scitranslmed.abj2658

[22] Purlyte E, Dhekne HS, Sarhan AR, et al Rab29 activation of the Parkinson’s disease-associated LRRK2 kinase. EMBO J. 2018;37:1–18. 29212815 10.15252/embj.201798099 PMC5753036

[23] Mir R, Tonelli F, Lis P, et al The Parkinson’s disease VPS35[D620N] mutation enhances LRRK2-mediated Rab protein phosphorylation in mouse and human. Biochem J. 2018;475:1861–1883. 29743203 10.1042/BCJ20180248 PMC5989534

[24] Dhekne HS, Tonelli F, Yeshaw WM, et al Genome-wide screen reveals Rab12 GTPase as a critical activator of Parkinson’s disease-linked LRRK2 kinase. Elife. 2023;12:e87098. 37874635 10.7554/eLife.87098 PMC10708890

[25] Wang X, Bondar VV, Davis OB, et al Rab12 is a regulator of LRRK2 and its activation by damaged lysosomes. Elife. 2023;12:e87255. 37874617 10.7554/eLife.87255 PMC10708889

[26] Gustavsson EK, Follett J, Trinh J, et al RAB32 Ser71Arg in autosomal dominant Parkinson’s disease: Linkage, association, and functional analyses. Lancet Neurol. 2024;23:603–614. 38614108 10.1016/S1474-4422(24)00121-2 PMC11096864

[27] Wilkinson MD, Dumontier M, Aalbersberg IJJ, et al The FAIR guiding principles for scientific data management and stewardship. Sci Data. 2016;3:160018. 26978244 10.1038/sdata.2016.18 PMC4792175

[28] Phung TK, Berndsen K, Shastry R, et al CURTAIN—A unique web-based tool for exploration and sharing of MS-based proteomics data. Proc Natl Acad Sci U S A. 2024;121:e2312676121. 38324566 10.1073/pnas.2312676121 PMC10873628

[29] Postuma RB, Berg D, Stern M, et al MDS clinical diagnostic criteria for Parkinson’s disease. Mov Disord. 2015;30:1591–1601. 26474316 10.1002/mds.26424

[30] Gaig C, Marti MJ, Ezquerra M, Rey MJ, Cardozo A, Tolosa E. G2019S *LRRK2* mutation causing Parkinson’s disease without Lewy bodies. J Neurol Neurosurg Psychiatry. 2007;78:626–628. 17210620 10.1136/jnnp.2006.107904 PMC2077973

[31] Sierra M, González-Aramburu I, Sánchez-Juan P, et al High frequency and reduced penetrance of LRRK2 G2019S mutation among Parkinson’s disease patients in Cantabria (Spain). Mov Disord. 2011;26:2343–2346. 21954089 10.1002/mds.23965

[32] Ruiz-Martínez J, Gorostidi A, Ibañez B, et al Penetrance in Parkinson’s disease related to the *LRRK2* R1441G mutation in the Basque country (Spain). Mov Disord. 2010;25:2340–2345. 20721916 10.1002/mds.23278

[33] Movement Disorder Society Task Force on Rating Scales for Parkinson's Disease . The Unified Parkinson’s Disease Rating Scale (UPDRS): Status and recommendations. Mov Disord. 2003;18:738–750. 12815652 10.1002/mds.10473

[34] Nasreddine ZS, Phillips NA, Bédirian V, et al The Montreal Cognitive Assessment, MoCA: A brief screening tool for mild cognitive impairment. J Am Geriatr Soc. 2005;53:695–699. 15817019 10.1111/j.1532-5415.2005.53221.x

[35] Simon-Sanchez J, Marti-Masso JF, Sanchez-Mut JV, et al Parkinson’s disease due to the R1441G mutation in Dardarin: a founder effect in the Basques. Mov Disord. 2006;21:1954–1959. 16991141 10.1002/mds.21114

[36] Ritchie ME, Phipson B, Wu D, et al Limma powers differential expression analyses for RNA-sequencing and microarray studies. Nucleic Acids Res. 2015;43:e47. 25605792 10.1093/nar/gkv007 PMC4402510

[37] Virtanen P, Gommers R, Oliphant TE, et al Scipy 1.0: Fundamental algorithms for scientific computing in Python. Nat Methods. 2020;17:261–272. 32015543 10.1038/s41592-019-0686-2 PMC7056644

[38] Seabold S, Perktold J. Statsmodels: Econometric and statistical modeling with python. In: *Proceedings of the 9th Python in Science Conference*. SciPy Conferences; 2010:92-96. doi:10.25080/majora-92bf1922-011

[39] Bekker-Jensen DB, Bernhardt OM, Hogrebe A, et al Rapid and site-specific deep phosphoproteome profiling by data-independent acquisition without the need for spectral libraries. Nat Commun. 2020;11:787. 32034161 10.1038/s41467-020-14609-1 PMC7005859

[40] Ogutu JO, Piepho HP, Schulz-Streeck T. A comparison of random forests, boosting and support vector machines for genomic selection. BMC Proc. 2011;5 Suppl 3(Suppl 3):S11. 10.1186/1753-6561-5-S3-S11 PMC3103196 21624167

[41] Chawla NV, Bowyer KW, Hall LO, Kegelmeyer WP. SMOTE: Synthetic minority over-sampling technique. J Artif Intell Res. 2002;16:321–357.

[42] Brodersen KH, Ong CS, Stephan KE, Buhmann JM. The balanced accuracy and its posterior distribution. In: *Proceedings—International Conference on Pattern Recognition*. ACM Digital Library; 2010:3121-3124. doi:10.1109/ICPR.2010.764

[43] Pedregosa F, Weiss R, Brucher M, et al Scikit-learn: Machine learning in python. J Mach Learn Res. 2011;12:2825–2830.

[44] Van Rossum G, Drake FL. *Python 3 reference manual*. Createspace Independent Pub.; 2009.

[45] Chen XW. Gene selection for cancer classification using bootstrapped genetic algorithms and support vector machines. In: *Proceedings of the 2003 IEEE Bioinformatics Conference, CSB 2003*. Springer; 2003:504-505. doi:10.1109/CSB.2003.1227389

[46] Gaudel R, Sebag M. Feature selection as a one-player game. In: *ICML 2010—Proceedings, 27th International Conference on Machine Learning*. ACM Digital Library; 2010:359-366.

[47] Zhou Y, Zhou B, Pache L, et al Metascape provides a biologist-oriented resource for the analysis of systems-level datasets. Nat Commun. 2019;10:1523. 30944313 10.1038/s41467-019-09234-6 PMC6447622

[48] Liu Q, Zhu D, Li N, et al Regulation of LRRK2 mRNA stability by ATIC and its substrate AICAR through ARE-mediated mRNA decay in Parkinson’s disease. EMBO J. 2023;42:e113410. 37366237 10.15252/embj.2022113410 PMC10390876

[49] Kalogeropulou AF, Freemantle JB, Lis P, Vides EG, Polinski NK, Alessi DR. Endogenous Rab29 does not impact basal or stimulated LRRK2 pathway activity. Biochem J. 2020;477:4397–4423. 33135724 10.1042/BCJ20200458 PMC7702304

[50] Kluss JH, Mazza MC, Li Y, et al Preclinical modeling of chronic inhibition of the Parkinson’s disease associated kinase LRRK2 reveals altered function of the endolysosomal system in vivo. Mol Neurodegener. 2021;16:17. 33741046 10.1186/s13024-021-00441-8 PMC7977595

[51] Ghelman J, Grewing L, Windener F, Albrecht S, Zarbock A, Kuhlmann T. SKAP2 as a new regulator of oligodendroglial migration and myelin sheath formation. Glia. 2021;69:2699–2716. 34324225 10.1002/glia.24066

[52] Takahashi T, Yamashita H, Nagano Y, et al Identification and characterization of a novel pyk2/related adhesion focal tyrosine kinase-associated protein that inhibits α-synuclein phosphorylation. J Biol Chem. 2003;278:42225–42233. 12893833 10.1074/jbc.M213217200

[53] Ayoubi R, Ryan J, Biddle MS, et al Scaling of an antibody validation procedure enables quantification of antibody performance in major research applications. Elife. 2023;12:RP91645. 37995198 10.7554/eLife.91645 PMC10666931

[54] Suzuki K, Dashzeveg N, Lu ZG, Taira N, Miki Y, Yoshida K. Programmed cell death 6, a novel p53-responsive gene, targets to the nucleus in the apoptotic response to DNA damage. Cancer Sci. 2012;103:1788–1794. 22712728 10.1111/j.1349-7006.2012.02362.x PMC7659207

[55] Erekat NS. Apoptosis and its role in Parkinson’s disease. In: *Parkinson’s Disease: Pathogenesis and Clinical Aspects*. Codon Publications; 2018:65-82.

[56] Tolosa E, Garrido A, Scholz SW, Poewe W. Challenges in the diagnosis of Parkinson’s disease. Lancet Neurol. 2021;20:385–397. 33894193 10.1016/S1474-4422(21)00030-2 PMC8185633

[57] Ao X, Zou L, Wu Y. Regulation of autophagy by the Rab GTPase network. Cell Death Differ. 2014;21:348–358. 24440914 10.1038/cdd.2013.187 PMC3921601

[58] Hirota Y, Yamashita S, Kurihara Y, et al Mitophagy is primarily due to alternative autophagy and requires the MAPK1 and MAPK14 signaling pathways. Autophagy. 2015;11:332–343. 25831013 10.1080/15548627.2015.1023047 PMC4502654

[59] Hirschberg Y, Valle-Tamayo N, Dols-Icardo O, et al Proteomic comparison between non-purified cerebrospinal fluid and cerebrospinal fluid-derived extracellular vesicles from patients with Alzheimer’s, Parkinson’s and Lewy body dementia. J Extracell Vesicles. 2023;12:e12383. 38082559 10.1002/jev2.12383 PMC10714029

[60] Vidyadhara DJ, Lee JE, Chandra SS. Role of the endolysosomal system in Parkinson’s disease. J Neurochem. 2019;150:487–506. 31287913 10.1111/jnc.14820 PMC6707858

[61] Erb ML, Moore DJ. LRRK2 and the endolysosomal system in Parkinson’s disease. J Parkinsons Dis. 2020;10:1271–1291. 33044192 10.3233/JPD-202138 PMC7677880

[62] Bulock KG, Beardsley GP, Anderson KS. The kinetic mechanism of the human bifunctional enzyme ATIC (5-amino-4-imidazolecarboxamide ribonucleotide transformylase/inosine 5′-monophosphate cyclohydrolase). A surprising lack of substrate channeling. J Biol Chem. 2002;277:22168–22174. 11948179 10.1074/jbc.M111964200

[63] Vergis JM, Beardsley GP. Catalytic mechanism of the cyclohydrolase activity of human aminoimidazole carboxamide ribonucleotide formyltransferase/inosine monophosphate cyclohydrolase. Biochemistry. 2004;43:1184–1192. 14756554 10.1021/bi035139b

[64] Byrns CN, Pitts MW, Gilman CA, Hashimoto AC, Berry MJ. Mice lacking selenoprotein P and selenocysteine lyase exhibit severe neurological dysfunction, neurodegeneration, and audiogenic seizures. J Biol Chem. 2014;289:9662–9674. 24519931 10.1074/jbc.M113.540682 PMC3975015

[65] Flinkman D, Hong Y, Gnjatovic J, et al Regulators of proteostasis are translationally repressed in fibroblasts from patients with sporadic and LRRK2-G2019S Parkinson’s disease. NPJ Parkinsons Dis. 2023;9:20. 36746972 10.1038/s41531-023-00460-w PMC9902458

[66] Jang Y, Pletnikova O, Troncoso JC, et al Mass spectrometry-based proteomics analysis of human substantia nigra from Parkinson’s disease patients identifies multiple pathways potentially involved in the disease. Mol Cell Proteomics. 2023;22:100452. 36423813 10.1016/j.mcpro.2022.100452 PMC9792365

[67] Grünewald A, Rygiel KA, Hepplewhite PD, Morris CM, Picard M, Turnbull DM. Mitochondrial DNA depletion in respiratory chain-deficient Parkinson disease neurons. Ann Neurol. 2016;79:366–378. 26605748 10.1002/ana.24571 PMC4819690

[68] Marchand A, Drouyer M, Sarchione A, Chartier-Harlin MC, Taymans JM. LRRK2 phosphorylation, more than an epiphenomenon. Front Neurosci. 2020;14:527. 32612495 10.3389/fnins.2020.00527 PMC7308437

[69] Harvey K, Outeiro TF. The role of LRRK2 in cell signalling. Biochem Soc Trans. 2018;47:197–207. 30578345 10.1042/BST20180464

[70] Rocha EM, Keeney MT, Di Maio R, De Miranda BR, Greenamyre JT. LRRK2 and idiopathic Parkinson’s disease. Trends Neurosci. 2022;45:224–236. 34991886 10.1016/j.tins.2021.12.002 PMC8854345

[71] Graham JM, Sagar HJ. A data-driven approach to the study of heterogeneity in idiopathic Parkinson’s disease: Identification of three distinct subtypes. Mov Disord. 1999;14:10–20. 9918339 10.1002/1531-8257(199901)14:1<10::aid-mds1005>3.0.co;2-4

[72] Lewis SJG, Foltynie T, Blackwell AD, Robbins TW, Owen AM, Barker RA. Heterogeneity of Parkinson’s disease in the early clinical stages using a data driven approach. J Neurol Neurosurg Psychiatry. 2005;76:343–348. 15716523 10.1136/jnnp.2003.033530 PMC1739569

[73] Bae E-J, Lee S-J. The LRRK2-RAB axis in regulation of vesicle trafficking and α-synuclein propagation. Biochim Biophys Acta Mol Basis Dis. 2020;1866:165632. 31812666 10.1016/j.bbadis.2019.165632

[74] Matsui T, Fukuda M. Rab12 regulates mTORC1 activity and autophagy through controlling the degradation of amino-acid transporter PAT4. EMBO Rep. 2013;14:450–457. 23478338 10.1038/embor.2013.32 PMC3642374

[75] Xenias HS, Chen C, Kang S, et al R1441c and G2019S LRRK2 knockin mice have distinct striatal molecular, physiological, and behavioral alterations. Commun Biol. 2022;5:1211. 36357506 10.1038/s42003-022-04136-8 PMC9649688

[76] Berndsen K, Lis P, Yeshaw WM, et al PPM1H phosphatase counteracts LRRK2 signaling by selectively dephosphorylating rab proteins. Elife. 2019;8:e50416. 31663853 10.7554/eLife.50416 PMC6850886

[77] Liu Z, Bryant N, Kumaran R, et al LRRK2 phosphorylates membrane-bound Rabs and is activated by GTP-bound Rab7L1 to promote recruitment to the trans-Golgi network. Hum Mol Genet. 2018;27:385–395. 29177506 10.1093/hmg/ddx410 PMC5886198

[78] Lesniak RK, Nichols RJ, Montine TJ. Development of mutation-selective LRRK2 kinase inhibitors as precision medicine for Parkinson’s disease and other diseases for which carriers are at increased risk. Front Neurol. 2022;13:1016040. 36388213 10.3389/fneur.2022.1016040 PMC9643380

[79] Leśniak RK, Nichols RJ, Schonemann M, et al Discovery of azaspirocyclic 1*H*-3,4,5-trisubstitued pyrazoles as novel G2019S-LRRK2 selective kinase inhibitors. Eur J Med Chem. 2022;242:114693. 36049274 10.1016/j.ejmech.2022.114693

[80] Lesńiak RK, Nichols RJ, Schonemann M, et al Discovery of 1*H*-pyrazole biaryl sulfonamides as novel G2019S-LRRK2 kinase inhibitors. ACS Med Chem Lett. 2022;13:981–988. 35707141 10.1021/acsmedchemlett.2c00116 PMC9190033

[81] Leśniak RK, Nichols RJ, Schonemann M, et al Discovery of G2019S-selective Leucine Rich Repeat Protein Kinase 2 inhibitors with in vivo efficacy. Eur J Med Chem. 2022;229:114080. 34992038 10.1016/j.ejmech.2021.114080

[82] Li X, Zhu H, Huang BT, et al RAB12-LRRK2 complex suppresses primary ciliogenesis and regulates centrosome homeostasis in astrocytes. Nat Commun. 2024;15:8434. 39343966 10.1038/s41467-024-52723-6 PMC11439917

[83] Garrido A, Santamaría E, Fernández-Irigoyen J, et al Differential phospho-signatures in blood cells identify *LRRK2* G2019S carriers in Parkinson’s disease. Mov Disord. 2022;37:1004–1015. 35049090 10.1002/mds.28927 PMC9306798

[84] Virreira Winter S, Karayel O, Strauss MT, et al Urinary proteome profiling for stratifying patients with familial Parkinson’s disease. EMBO Mol Med. 2021;13:e13257. 33481347 10.15252/emmm.202013257 PMC7933820

[85] Hadisurya M, Li L, Kuwaranancharoen K, et al Quantitative proteomics and phosphoproteomics of urinary extracellular vesicles define putative diagnostic biosignatures for Parkinson’s disease. Commun Med (Lond). 2023;3:64. 37165152 10.1038/s43856-023-00294-w PMC10172329

[86] Kluss JH, Beilina A, Williamson CD, Lewis PA, Cookson MR, Bonet-Ponce L. Lysosomal positioning regulates Rab10 phosphorylation at LRRK2^+^ lysosomes. Proc Natl Acad Sci U S A. 2022;119:e2205492119. 36256825 10.1073/pnas.2205492119 PMC9618077

[87] Perez-Riverol Y, Bai J, Bandla C, et al The PRIDE database resources in 2022: A hub for mass spectrometry-based proteomics evidences. Nucleic Acids Res. 2022;50(D1):D543–D552. 34723319 10.1093/nar/gkab1038 PMC8728295

