# Supplementary material for: In-depth mass-spectrometry reveals phospho-RAB12 as a blood biomarker of G2019S LRRK2-driven Parkinson’s disease
Source: Brain. 2024 Dec 20;148(6):2075–92. doi: 10.1093/brain/awae404 (PMC12129731; doi:10.1093/brain/awae404)
Supplement: awae404_Supplementary_Data [file awae404_supplementary_data.zip › brain-2024-01397-File009.pdf]

SUPPLEMENTARY MATERIAL

pSer106 RAB12 immunoblotting of 1-year follow-up PBMCs

To assess the levels of pSer106 RAB12 and other markers over time, we isolated PBMCs using 40 ml of blood from a subset of subjects from Clínic-Barcelona (n=48), collected >1-year after DIA-MS, including G2019S L2PD (n=12), G2019S L2NMCs (n=6), iPD (n=15), and controls (n=15). Cell lysates were mixed with the NuPAGE LDS Sample Buffer (Life Technologies) supplemented 5% (v/v) 2-mercaptoethanol and heated to 90°C for 10 minutes. 10 µg of protein was loaded onto NuPAGE 4–12% Bis-Tris Midi Gels (Thermo Fisher Sci. #WG1403BX10). Following the manufacturer's instructions, samples were electrophoresed in NuPAGE MOPs SDS running buffer (Thermo Fisher). Protein transfer was performed onto nitrocellulose membranes (GE Healthcare, Amersham Protran Supported 0.45 µm NC) at 100 V for 90 min on ice in transfer buffer (48 mM Tris–HCl and 39 mM glycine supplemented with 20% methanol) or through turbotransfer. MLi-2 treated protein extracts were run in 4–15% Mini-Protean TGX precast protein gels (Bio-Rad #4561084) and transferred onto trans-Blot Turbo Mini 0.2 µm Nitrocellulose Transfer (Bio-Rad #1704158) through a Trans-Blot Turbo Transfer System (Bio-Rad) following the manufacturer's instructions. Following the transfer, membranes were blocked using 5 (w/v) skim milk dissolved in TBS-T (20 mM Tris–HCl, pH 7.5, 150 mM NaCl, and 0.1% (v/v) Tween 20) at room temperature for 30 minutes. Membranes were washed once with TBS-T before 4°C overnight incubation with primary antibodies diluted in 5% BSA (Sigma) and dissolved in TBS-T. Membranes were washed 3x with TBS-T for 5 min each before incubation with secondary antibodies diluted in TBS-T at RT for 1 hour. Following secondary incubation, membranes were washed 3x with TBS-T for 10 min each. Acquisition of protein bands was performed with near-infrared fluorescent detection using the Odyssey CLx imaging system and quantified using the Image Studio software. The sheep monoclonal anti-total RAB12 antibody was purified by MRC PPU Reagents and Services at the University of Dundee and was used at a final concentration of 1 µg/ml. Mouse monoclonal anti-GAPDH (Santa Cruz Biotechnology, sc-32233) was employed at 1:10,000. The following antibodies were used at a 1:1,000 dilution, namely, in-house generated mouse monoclonal anti-total LRRK2, rabbit monoclonal anti-pSer935 LRRK2 (Abcam #ab133450), rabbit polyclonal anti-total RAB12 (Protein Tech. #18843-1-AP), rabbit monoclonal anti-pSer106 RAB12 (Abcam

#ab256487), rabbit monoclonal anti-RAB9A (Cell Signalling Tech. #5118), rabbit monoclonal anti-LAMP1 (Cell Signalling Tech. #9091), and mouse monoclonal anti-Tubulin (Cell Signalling Tech. #3873S) (**Suppl. Table 3**). Lastly, secondary antibodies, anti-mouse (Licor #926-68022), anti-rabbit (Licor #926-32213), and anti-goat (Licor #926-68074), which has sheep cross-reactivity, were used at a 1:10,000 dilution.

pSer106 RAB12 immunoblotting after MLi-2 LRRK2 inhibition in fresh PBMCs

To assess the response of pSer106 RAB12 to MLi-2 LRRK2 inhibition we additionally isolated PBMCs from 40 ml of peripheral blood of an additional set of subjects (n=10), including G2019S L2PD (n=3), R1441G L2PD (n=1), iPD (n=1), and healthy controls (n=5). These cells were collected > 2 years after DIA-MS. Each 20 ml of freshly extracted PBMCs was aliquoted into two technical replicates of two tubes of 5 ml each to perform MLi-2 pharmacological inhibition following the Dundee PBMCs isolation protocol (doi: dx.doi.org/10.17504/protocols.io.bnhxmb7n) (from whole blood). Briefly, each technical replicate was treated with either 200 nM MLi-2 LRRK2 inhibitor or an equivalent volume of DMSO (5 µl) for 30 min at room temperature. Next, treated PBMCs were centrifuged at 355g for 5 min. Each pellet was resuspended in 1 ml of PBS containing 2% FBS with or without 200 nM MLi-2 and transferred into an Eppendorf tube. After centrifugation at 300 g for 3 min, treated PBMC pellets were lysed with 70 µl of ice-cold lysis buffer (50 mM Tris–HCl, pH 7.5, 1% Triton X-100, 1 mM EGTA, 1 mM sodium orthovanadate, 50 mM NaF, 0.1% 2-mercaptoethanol, 10 mM 2-glycerophosphate, 5 mM sodium pyrophosphate, 0.1 µg/ml microcystin-LR, 270 mM sucrose, 0.5 mM DIFP and Complete EDTA-free protease inhibitor cocktail) on ice for 10min. Then, treated PBMC cell lysates were centrifuged at 14,000 rpm 15 min at 4°C and supernatant was collected. Protein concentrations were determined at a 1:15 dilution through the Bradford assay. Protein extracts were immediately used for immunoblotting or snap-frozen and stored at −80°C. Subsequent immunoblot analysis was done as described above, with some specificities: Brielfly, MLi-2 treated protein extracts were electrophoresed in 4–15% Mini-Protean TGX precast protein gels (Bio-Rad #4561084) and transferred onto trans-Blot Turbo Mini 0.2 µm Nitrocellulose Transfer (Bio-Rad #1704158) through a Trans-Blot Turbo Transfer System (Bio-Rad) following the manufacturer's instructions.

Acquisition of protein bands for MLi-2 protein extracts was performed on a LAS4000 system and processed through the BandPeak plugin (doi: dx.doi.org/10.17504/protocols.io.7vghn3w) at the ImageJ software. For this experiment, mouse monoclonal anti-RAB10 (Merck #SAB5300028), rabbit recombinant anti-pThr73 RAB10 (Abcam #ab230261), rabbit polyclonal anti-RAB12 (Protein Tech. #18843-1-AP), rabbit monoclonal anti-p106 RAB12 (Abcam #ab256487) were used at a 1:1,000 dilution (**Suppl. Table 3**). We used goat anti-rabbit (Thermo Fisher Sci. #31460) and goat anti-mouse (Abcam #ab205719) as secondary antibodies.

Summary of expanded LRRK2 phospho-/proteomic analyses

Expanded LRRK2 phospho-/proteomic analyses by disease status, mutation and centre are available at *Brain* online (**Suppl. Material**).

R scripts

R scripts for differential LRRK2 phospho-proteomic analyses are available at *Brain* online (**Suppl. Material**), and also as a cloud weblink (doi.org/10.5281/zenodo.13774022).

A

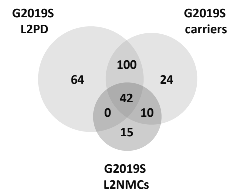

Names	total	elements
G2019S L2NMC G2019S L2PD G2019S carriers	42	HDGF KARS1 MOB1B NR2C2AP CFP SUCLG2 ILK PDCD6 ASH2L SIGIRR PITHD1 RNF113A TOMM20 H2AC21 SRSF1 PSMC5 COG1 NDUFB8 PPIL3 IFITM1 ARHGAP45 YTHDC1 PFDN5 RBBP7 ATIC MT2A UQCRB ZBP1 ELAVL1 LAMP1 METTL9 UGP2 ALOX5AP RPL11 ASPSCR1 ZC3HAV1L STOML2 MAP2K1 SF3B4 LRRC8D SH3GLB2 FANCM
G2019S L2PD G2019S carriers	100	L3HYPDH SMNDC1 DDX3Y GFER PRTFDC1 TPPP3 ASAP2 PHF5A PSPC1 RETN TMA16 BCL2 CASP7 HSD17B10 JPT1 AMPD2 DNM3 PPP1R14A PAIP1 MEN1 PITPNM2 MED22 RCOR1 POLR2J VAV2 CBFB CEBPB HMGN4 ATP8A1 CWF19L1 MARCKSL1 RPL36 LYAR SH3KBP1 ZNF384 GK GOSR1 ARL6IP1 DUSP3 ACAD8 BTF3 ACTG1 GABARAPL2 HLA-DOA RASSF5 MRPS14 SEC11C VKORC1L1 RPL37A 12Å LSM PAAF1 VPS50 CMPK2 KRAS IGKV3-20 ERAP1 PRKCB TCEAL5 NASP CRELD1 ATP5PD HNRNPA2B1 LYSMD2 MMP1 NMT1 STK17B MRPL58 CAMK4 RIN3 PLEKHF1 CAP1 ARL8B TFG SHMT2 5Å LSM PCSK6 MYO5A WASHC3 TAP2 RAB9A ADO GATD1 FUBP1 FNBP4 TSPAN33 AAMDC RGCC CCDC9 AFAP1 FXR1 SAFB MIF4GD UBP1 SCLY EXOC6B NMRAL1 CCT7 EMC1 ACLY OPA3
G2019S L2NMC G2019S carriers	10	ANKS1A HLA-F TXNRD2 PAK1 APLP2 ORMDL3 PHKB UTP18 AP2B1 PGGT1B
G2019S L2PD	64	HNRNPDL RPL23 AASDHPPT SLC4A1 BRD4 NUP54 BRI3BP NT5DC1 TRMT112 AP2M1 GC FTH1 FAF1 LIMA1 RCN1 TOMM5 CLNS1A PDK1 TRABD TSPAN15 ETV6 AGPAT3 MAPK8 MAGED2 RTCA RTF2 DYRK1A PTMS ME1 GSTZ1 BMP2K TBL2 PUF60 CARS2 SCAF4 PRAF2 WBP2 APOL3 NOP14 MTFR1L SRGAP2 SART3 TSPAN14 CTCF RUSF1 ETFB MYH11 NUDT18 IST1 PHKA2 SMYD3 JCHAIN LPA GOLIM4 DDX47 PCTP VRK3 ATP2A3 UBE2J1 PSME3IP1 BCAP29 TRAPPC1 UBQLN4 PRKDC
G2019S L2NMC	15	STK24 RPL17 PCBD2 GATD3B HNRNPD PDE5A MTRES1 RBM8A CDV3 GRK6 UQCRH ITPRID2 APOE CERS2 NDE1
G2019S carriers	24	DMAP1 CSTA GSTT2 SDHC VIL1 SNRNP40 GUCY1B1 LILRA2 S100P MFSD1 HNRNPU ARHGAP17 LRBA MAPK13 APCS DPP9 VPS36 GSTT1 INTS10 IGKC ECM1 UBXN1 IL4I1 ACSBG1

B

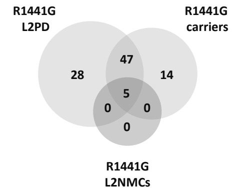

Names	total	elements
R1441G L2NMC R1441G L2PD R1441G carriers	5	HDGF AASDHPPT PDCD6 NDUFB8 MTRES1
R1441G L2PD R1441G carriers	47	ARHGAP17 AIF1 ERAP1 CCAR2 ZNF207 LAMP1 NASP UGP2 GRK6 LAMTOR4 RPL17 ATG7 ILK PITHD1 NT5DC1 JPT1 FAF1 CLASP2 LRRFIP1 IST1 TOMM5 NKIRAS2 NT5C2 RAB9A TSPAN15 PPIL2 SEC24A HDGFL2 ARHGAP45 SSU72 PFDN5 SH3GLB2 LRRC8D ATP5MJ ATIC NDUFC2 SCLY MBOAT7 UBE2H ELMOD2 PSMD13 CALM1 HLA-DOA EMC1 PUF60 PLEC CHCHD2
R1441G L2PD	28	FAM120A LSM12 PSAT1 KRAS MCM3 TBCB NDUFB1 GSK3B MGAT2 BRD4 CTDSP1 TMEM11 KCMF1 GC RPL11 SRSF1 WARS1 ARL8B BABAM1 STMN1 NDUFA11 EIF3L IGHV3-72 RPL7L1 BCL7C MYADM UBQLN4 DOCK8
R1441G carriers	14	TEP1 HNRNPH3 RPL26 RABEP1 UBTF TMEM41B PITPNC1 HNRNPD FUBP1 HMGN4 PNKD KIAA2013 THOC6 HNRNPU

C

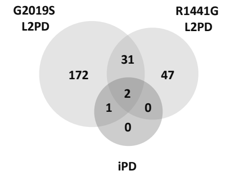

Names	total	elements
G2019S L2PD R1441G L2PD iPD	2	SRSF1 LAMP1
G2019S L2PD iPD	1	UQCRB
G2019S L2PD R1441G L2PD	31	HDGF AASDHPPT ILK BRD4 PDCD6 PITHD1 NT5DC1 JPT1 GC FAF1 TOMM5 NDUFB8 TSPAN15 ARHGAP45 PFDN5 ATIC HLA-DOA PUF60 KRAS ERAP1 NASP UGP2 RPL11 IST1 ARL8B RAB9A LRRC8D SH3GLB2 UBQLN4 SCLY EMC1
G2019S L2PD	172	HNRNPDL KARS1 L3HYPDH SMNDC1 MOB1B DDX3Y GFER PRTFDC1 RPL23 TPPP3 ASAP2 PHF5A NR2C2AP PSPC1 RETN SLC4A1 TMA16 CFP SUCLG2 BCL2 ASH2L NUP54 BRI3BP SIGIRR CASP7 HSD17B10 TRMT112 RNF113A AMPD2 AP2M1 FTH1 TOMM20 DNM3 PPP1R14A LIMA1 H2AC21 RCN1 CLNS1A PSMC5 PAIP1 MEN1 COG1 PPIL3 PITPNM2 PDK1 TRABD ETV6 MED22 RCOR1 POLR2J AGPAT3 VAV2 MAPK8 IFITM1 CBFB CEBPB MAGED2 HMGN4 RTCA RTF2 ATP8A1 CWF19L1 MARCKSL1 RPL36 LYAR SH3KBP1 DYRK1A YTHDC1 PTMS ZNF384 GK RBBP7 MT2A GOSR1 ME1 ARL6IP1 GSTZ1 DUSP3 ACAD8 BTF3 ACTG1 BMP2K GABARAPL2 RASSF5 TBL2 MRPS14 ZBP1 SEC11C VKORC1L1 CARS2 RPL37A SCAF4 12Å LSM PAAF1 VPS50 ELAVL1 CMPK2 PRAF2 WBP2 IGKV3-20 APOL3 NOP14 METTL9 MTFR1L PRKCB TCEAL5 SRGAP2 CRELD1 SART3 ATP5PD TSPAN14 CTCF HNRNPA2B1 RUSF1 LYSMD2 ALOX5AP MMP1 ETFB NMT1 STK17B MYH11 ASPSCR1 MRPL58 NUDT18 CAMK4 RIN3 PHKA2 PLEKHF1 CAP1 SMYD3 TFG SHMT2 5Å LSM PCSK6 JCHAIN MYO5A LPA WASHC3 ZC3HAV1L STOML2 TAP2 GOLIM4 ADO DDX47 PCTP GATD1 MAP2K1 VRK3 ATP2A3 FUBP1 FNBP4 UBE2J1 TSPAN33 AAMDC RGCC CCDC9 SF3B4 AFAP1 FXR1 SAFB MIF4GD PSME3IP1 BCAP29 UBP1 TRAPPC1 PRKDC EXOC6B NMRAL1 CCT7 ACLY OPA3 FANCM
R1441G L2PD	47	PSAT1 CCAR2 ZNF207 MCM3 TBCB NDUFB1 LAMTOR4 RPL17 WARS1 NT5C2 IGHV3-72 PPIL2 SEC24A ATP5MJ UBE2H ELMOD2 PSMD13 CALM1 MTRES1 CHCHD2 PLEC ARHGAP17 FAM120A AIF1 LSM12 GSK3B GRK6 MGAT2 ATG7 CTDSP1 TMEM11 KCMF1 CLASP2 LRRFIP1 NKIRAS2 BABAM1 STMN1 EIF3L NDUFA11 RPL7L1 HDGFL2 BCL7C SSU72 MYADM NDUFC2 DOCK8 MBOAT7

D

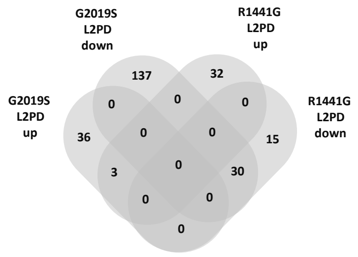

Names	total	elements
G2019S L2PD up R1441G L2PD up	3	FAF1 TOMM5 EMC1
G2019S L2PD down R1441G L2PD down	30	HDGF AASDHPPT ILK BRD4 PDCD6 PITHD1 NT5DC1 JPT1 GC SRSF1 NDUFB8 TSPAN15 ARHGAP45 PFDN5 ATIC HLA-DOA PUF60 KRAS ERAP1 LAMP1 NASP UGP2 RPL11 IST1 ARL8B RAB9A LRRC8D SH3GLB2 UBQLN4 SCLY
G2019S L2PD up	36	L3HYPDH MOB1B PRTFDC1 AP2M1 PPP1R14A RCN1 PAIP1 MEN1 PITPNM2 PDK1 TRABD AGPAT3 RTCA ATP8A1 GK ME1 BMP2K VKORC1L1 CARS2 PAAF1 WBP2 MTFR1L PRKCB TSPAN14 RUSF1 MYH11 CAP1 SMYD3 PCSK6 MYO5A ZC3HAV1L PCTP ATP2A3 RGCC SAFB TRAPPC1
G2019S L2PD down	137	HNRNPDL KARS1 SMNDC1 DDX3Y GFER RPL23 TPPP3 ASAP2 PHF5A NR2C2AP PSPC1 RETN SLC4A1 TMA16 CFP SUCLG2 BCL2 ASH2L NUP54 BRI3BP SIGIRR CASP7 HSD17B10 TRMT112 RNF113A AMPD2 FTH1 TOMM20 DNM3 LIMA1 H2AC21 CLNS1A PSMC5 COG1 PPIL3 ETV6 MED22 RCOR1 POLR2J VAV2 MAPK8 IFITM1 CBFB CEBPB MAGED2 HMGN4 RTF2 CWF19L1 MARCKSL1 RPL36 LYAR SH3KBP1 DYRK1A YTHDC1 PTMS ZNF384 RBBP7 MT2A GOSR1 ARL6IP1 GSTZ1 DUSP3 ACAD8 BTF3 UQCRB ACTG1 GABARAPL2 RASSF5 TBL2 MRPS14 ZBP1 SEC11C RPL37A SCAF4 12Å LSM VPS50 ELAVL1 CMPK2 PRAF2 IGKV3-20 APOL3 NOP14 METTL9 TCEAL5 SRGAP2 CRELD1 SART3 ATP5PD CTCF HNRNPA2B1 LYSMD2 ALOX5AP MMP1 ETFB NMT1 STK17B ASPSCR1 MRPL58 CAMK4 RIN3 NUDT18 PHKA2 PLEKHF1 TFG SHMT2 5Å LSM JCHAIN LPA WASHC3 STOML2 TAP2 GOLIM4 ADO DDX47 GATD1 MAP2K1 VRK3 FUBP1 FNBP4 UBE2J1 TSPAN33 AAMDC CCDC9 SF3B4 AFAP1 FXR1 MIF4GD PSME3IP1 BCAP29 UBP1 PRKDC EXOC6B NMRAL1 CCT7 ACLY OPA3 FANCM
R1441G L2PD up	32	PSAT1 CCAR2 ZNF207 MCM3 TBCB NDUFB1 NT5C2 PPIL2 SEC24A ATP5MJ UBE2H ELMOD2 PSMD13 CALM1 CHCHD2 PLEC FAM120A AIF1 MGAT2 ATG7 TMEM11 LRRFIP1 BABAM1 EIF3L NDUFA11 RPL7L1 HDGFL2 BCL7C SSU72 NDUFC2 DOCK8 MBOAT7
R1441G L2PD down	15	LAMTOR4 RPL17 WARS1 IGHV3-72 MTRES1 ARHGAP17 LSM12 GSK3B GRK6 CTDSP1 KCMF1 CLASP2 NKIRAS2 STMN1 MYADM

E

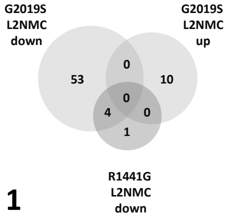

Names	total	elements
G2019S L2NMC down R1441G L2NMC down	4	HDGF PDCD6 NDUFB8 MTRES1
G2019S L2NMC up	10	MOB1B STK24 ORMDL3 PHKB ZC3HAV1L PDE5A ITPRID2 APOE CERS2 APLP2
G2019S L2NMC down	53	KARS1 ANKS1A ELAVL1 NR2C2AP METTL9 LAMP1 UGP2 GRK6 CFP RPL17 SUCLG2 ILK HLA-F ASH2L TXNRD2 SIGIRR PITHD1 UTP18 PAK1 RNF113A ALOX5AP TOMM20 RPL11 ASPSCR1 PCBD2 H2AC21 SRSF1 PSMC5 GATD3B COG1 UQCRH PPIL3 HNRNPD STOML2 MAP2K1 IFITM1 ARHGAP45 YTHDC1 SF3B4 PFDN5 LRRC8D SH3GLB2 RBBP7 ATIC MT2A AP2B1 UQCRB RBM8A PGGT1B CDV3 FANCM ZBP1 NDE1
R1441G L2NMC down	1	AASDHPPT

Suppl. Fig. 1

Suppl. Fig. 1. Comparison of proteome hits identified across different groups

Venn diagrams depicting common and specific hits in various groups as compared to healthy controls. (**A**) Differential hits found at various G2019S carrier groups, PD manifesting and non-manifesting, compared to controls. (**B**) Hits from various R1441G carrier groups, symptomatic and asymptomatic, vs controls. (**C**) Common and specific hits were observed in the different PD patient groups, i.e., G2019S L2PD, R1441G L2PD, and iPD. (**D**) Differential hits among L2PD patients carrying either the G2019S or the R1441G mutations, stratified by up and down-regulated hits. (**E**) Differential hits among L2NMCs carrying either the G2019S or the R1441G mutations, as analysed segregated by up and down-regulated hits.

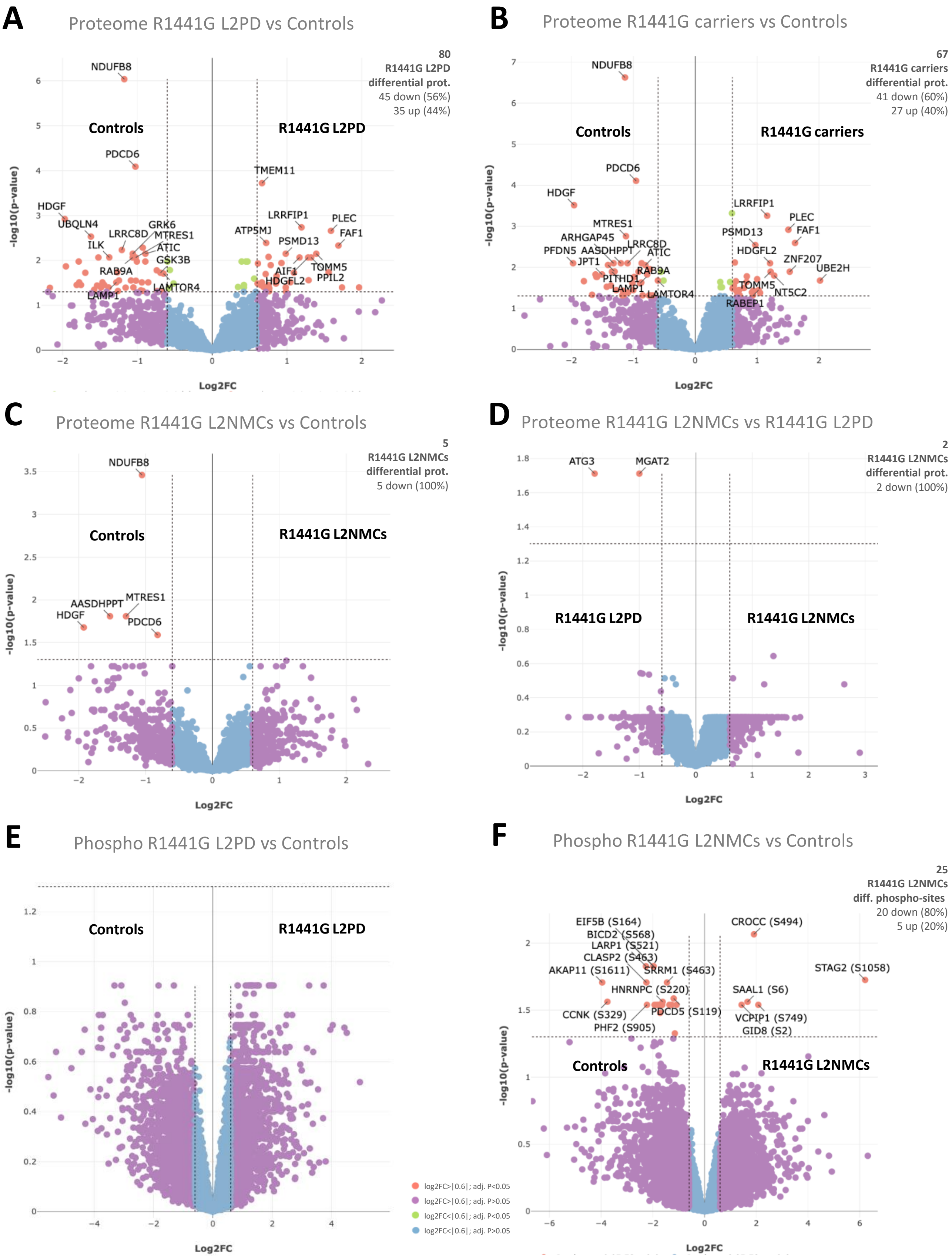
A
Proteome R1441G L2PD vs Controls
80
R1441G L2PD
differential prot.
45 down (56%)
35 up (44%)
Controls
R1441G L2PD
B
Proteome R1441G carriers vs Controls
67
R1441G carriers
differential prot.
41 down (60%)
27 up (40%)
Controls
R1441G carriers
C
Proteome R1441G L2NMCs vs Controls
5
R1441G L2NMCs
differential prot.
5 down (100%)
Controls
R1441G L2NMCs
D
Proteome R1441G L2NMCs vs R1441G L2PD
2
R1441G L2NMCs
differential prot.
2 down (100%)
R1441G L2PD
R1441G L2NMCs
E
Phospho R1441G L2PD vs Controls
Controls
R1441G L2PD
F
Phospho R1441G L2NMCs vs Controls
25
R1441G L2NMCs
diff. phospho-sites
20 down (80%)
5 up (20%)
Controls
R1441G L2NMCs
-log10(p-value)
Log2FC
log2FC>|0.6|; adj. P<0.05
log2FC>|0.6|; adj. P>0.05
log2FC<|0.6|; adj. P<0.05
log2FC<|0.6|; adj. P>0.05

Suppl. Fig. 2

Suppl. Fig. 2. Proteome and phospho-proteome differential analysis of R1441G carriers

Consistently across the study, the significance cut-off for R1441G proteome and phospho-proteome analyses was also set at a $log_2FC>|0.6|$ and an FDR multiple-testing adj. $P<0.05$. A legend colour code applying to all panels is shown at the bottom of the figure, depicting statistically significant hits as red dots. (**A**) Volcano plot of the proteome differential analysis in R1441G L2PD vs healthy controls, with Curtain weblinks to access raw and differential analysis data, showing proteins up-regulated in R1441G L2PD as red dots on the right, and proteins up-regulated in controls (i.e., down-regulated in R1441G L2PD) as red dots on the left (Curtain). (**B**) Volcano plot of the proteome differential analysis in R1441G carriers as a whole, i.e., L2PD and L2NMCs, vs healthy controls (Curtain). (**C**) Volcano plot showing the proteome differential analysis between R1441G L2NMCs and healthy controls (Curtain). (**D**) Volcano plot representing the proteome comparison between R1441G L2NMCs and R1441G L2PD (Curtain). (**E**) Volcano plot of the phospho-proteome differential analysis between R1441G L2PD vs controls (Curtain PTM). (**F**) Volcano plot of the phospho-proteome comparison of R1441G L2NMCs and controls (Curtain PTM). Curtain weblinks provide access to the differential analyses.

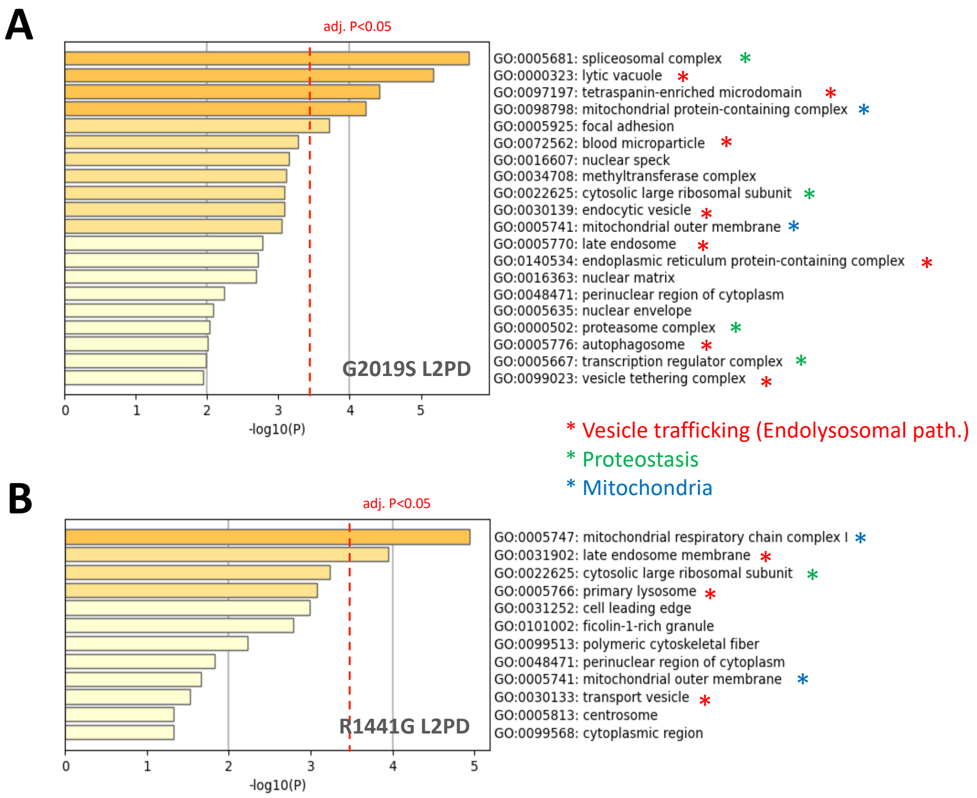
A
adj. P<0.05
GO:0005681: spliceosomal complex *
GO:0000323: lytic vacuole *
GO:0097197: tetraspanin-enriched microdomain *
GO:0098798: mitochondrial protein-containing complex *
GO:0005925: focal adhesion
GO:0072562: blood microparticle *
GO:0016607: nuclear speck
GO:0034708: methyltransferase complex
GO:0022625: cytosolic large ribosomal subunit *
GO:0030139: endocytic vesicle *
GO:0005741: mitochondrial outer membrane *
GO:0005770: late endosome *
GO:0140534: endoplasmic reticulum protein-containing complex *
GO:0016363: nuclear matrix
GO:0048471: perinuclear region of cytoplasm
GO:0005635: nuclear envelope
GO:0000502: proteasome complex *
GO:0005776: autophagosome *
GO:0005667: transcription regulator complex *
GO:0099023: vesicle tethering complex *
G2019S L2PD
0 1 2 3 4 5
-log10(P)
* Vesicle trafficking (Endolysosomal path.)
* Proteostasis
* Mitochondria
B
adj. P<0.05
GO:0005747: mitochondrial respiratory chain complex I *
GO:0031902: late endosome membrane *
GO:0022625: cytosolic large ribosomal subunit *
GO:0005766: primary lysosome *
GO:0031252: cell leading edge
GO:0101002: ficolin-1-rich granule
GO:0099513: polymeric cytoskeletal fiber
GO:0048471: perinuclear region of cytoplasm
GO:0005741: mitochondrial outer membrane *
GO:0030133: transport vesicle *
GO:0005813: centrosome
GO:0099568: cytoplasmic region
R1441G L2PD
0 1 2 3 4 5
-log10(P)

Suppl. Fig. 3

Suppl. Fig. 3. Proteome functional analysis of G2019S and R1441G patients

Comparative gene ontology (GO) enrichment analysis of the differential proteins observed in G2019S and R1441G L2PD was done in Metascape under an FDR multiple-testing adj. $P<0.05$, here denoted as a dashed red line. (**A**) GO enrichment plot in G2019S L2PD vs controls. (**B**) GO enrichment plot in R1441G L2PD vs controls. Proteome changes related to both mutations showed affection of similar functional terms affecting the endolysosomal pathway (red asterisks), protein homeostasis (green), and mitochondria function (blue).

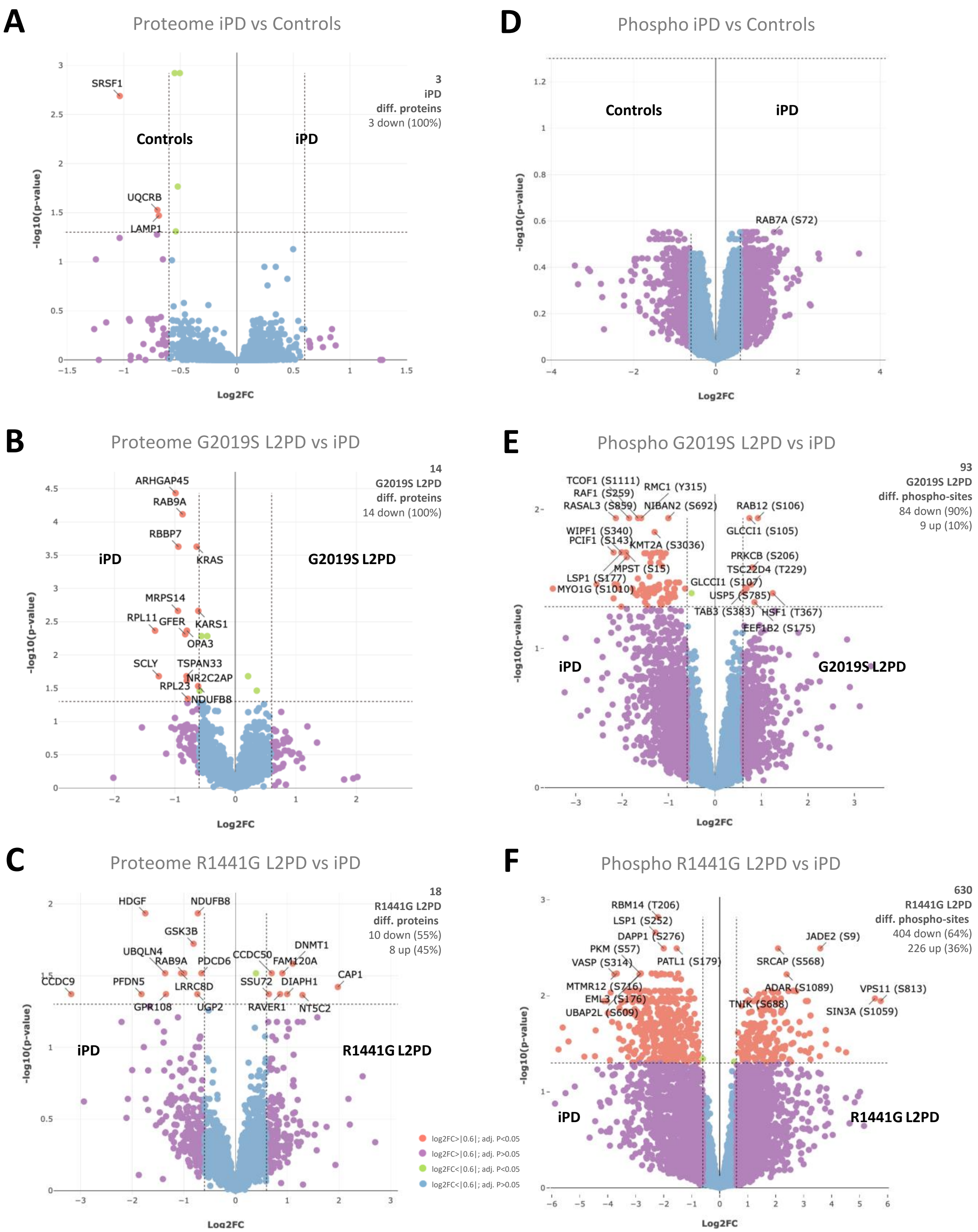
A
Proteome iPD vs Controls
3
iPD
diff. proteins
3 down (100%)
Controls
iPD
SRSF1
UQCRB
LAMP1
-log10(p-value)
Log2FC
D
Phospho iPD vs Controls
Controls
iPD
RAB7A (S72)
-log10(p-value)
Log2FC
B
Proteome G2019S L2PD vs iPD
14
G2019S L2PD
diff. proteins
14 down (100%)
iPD
G2019S L2PD
ARHGAP45
RAB9A
RBBP7
KRAS
MRPS14
RPL11
GFER
KARS1
OPA3
SCLY
TSPAN33
NR2C2AP
RPL23
NDUFB8
-log10(p-value)
Log2FC
E
Phospho G2019S L2PD vs iPD
93
G2019S L2PD
diff. phospho-sites
84 down (90%)
9 up (10%)
TCOF1 (S1111)
RAF1 (S259)
RMC1 (Y315)
RASAL3 (S859)
NIBAN2 (S692)
RAB12 (S106)
GLCCI1 (S105)
WIPF1 (S340)
PCIF1 (S143)
KMT2A (S3036)
PRKCB (S206)
TSC22D4 (T229)
MPST (S15)
LSP1 (S177)
MYO1G (S1010)
GLCCI1 (S107)
USP5 (S785)
TAB3 (S383)
HSF1 (T367)
EEF1B2 (S175)
iPD
G2019S L2PD
-log10(p-value)
Log2FC
C
Proteome R1441G L2PD vs iPD
18
R1441G L2PD
diff. proteins
10 down (55%)
8 up (45%)
HDGF
NDUFB8
GSK3B
DNMT1
UBQLN4
CCDC50
FAM120A
RAB9A
PDCD6
CAP1
CCDC9
PFDN5
LRRC8D
SSU72
DIAPH1
GPR108
UGP2
RAVER1
NT5C2
iPD
R1441G L2PD
-log10(p-value)
Log2FC
log2FC>|0.6|; adj. P<0.05
log2FC>|0.6|; adj. P>0.05
log2FC<|0.6|; adj. P<0.05
log2FC<|0.6|; adj. P>0.05
F
Phospho R1441G L2PD vs iPD
630
R1441G L2PD
diff. phospho-sites
404 down (64%)
226 up (36%)
RBM14 (T206)
LSP1 (S252)
DAPP1 (S276)
JADE2 (S9)
PKM (S57)
VASP (S314)
PATL1 (S179)
SRCAP (S568)
MTMR12 (S716)
ADAR (S1089)
VPS11 (S813)
EML3 (S176)
TNIK (S688)
UBAP2L (S609)
SIN3A (S1059)
iPD
R1441G L2PD
-log10(p-value)
Log2FC

Suppl. Fig. 4

Suppl. Fig. 4. Proteome and phospho-proteome analysis of iPD compared to L2PD

Curtain weblinks provide access to raw and differential analysis data under a significance cut-off of a $log_2FC>|0.6|$ and an FDR multiple-testing adj. $P<0.05$. The legend colour code shows hits categorisation based on statistical significance and applies to all the panels. (**A**) Volcano plot of the proteome analysis in iPD vs controls showing no differential hit under the statistical cut-off used (Curtain). (**B**) Volcano plot representing protein differences between iPD and G2019S L2PD, showing iPD up-regulated proteins as red dots on the left (Curtain). (**C**) Volcano plot representing protein changes between iPD and R1441G L2PD, with iPD up-regulated proteins as red dots on the left, and iPD down-regulated (i.e., up-regulated in R1441G L2PD) as red dots on the right (Curtain) (**D**) Volcano plot of the phospho-proteome analysis in iPD vs controls showing no differential hit, despite being iPD the groups with larger sample size in the study (Curtain PTM). (**E**) Volcano plot representing phospho-protein differences between iPD and G2019S L2PD, with proteins hyper-phosphorylated in G2019S L2PD as red dots on the right, showing pSer106 RAB12 as top hit, and proteins hyper-phosphorylated in iPD (i.e., hypo-phosphorylated in G2019S L2PD) as red dots on the left (Curtain PTM). (**F**) Similar analysis as in the previous panel, here comparing the phospho-proteome comparison between iPD and R1441G L2PD (Curtain PTM). Curtain weblinks provide access to the differential analyses.

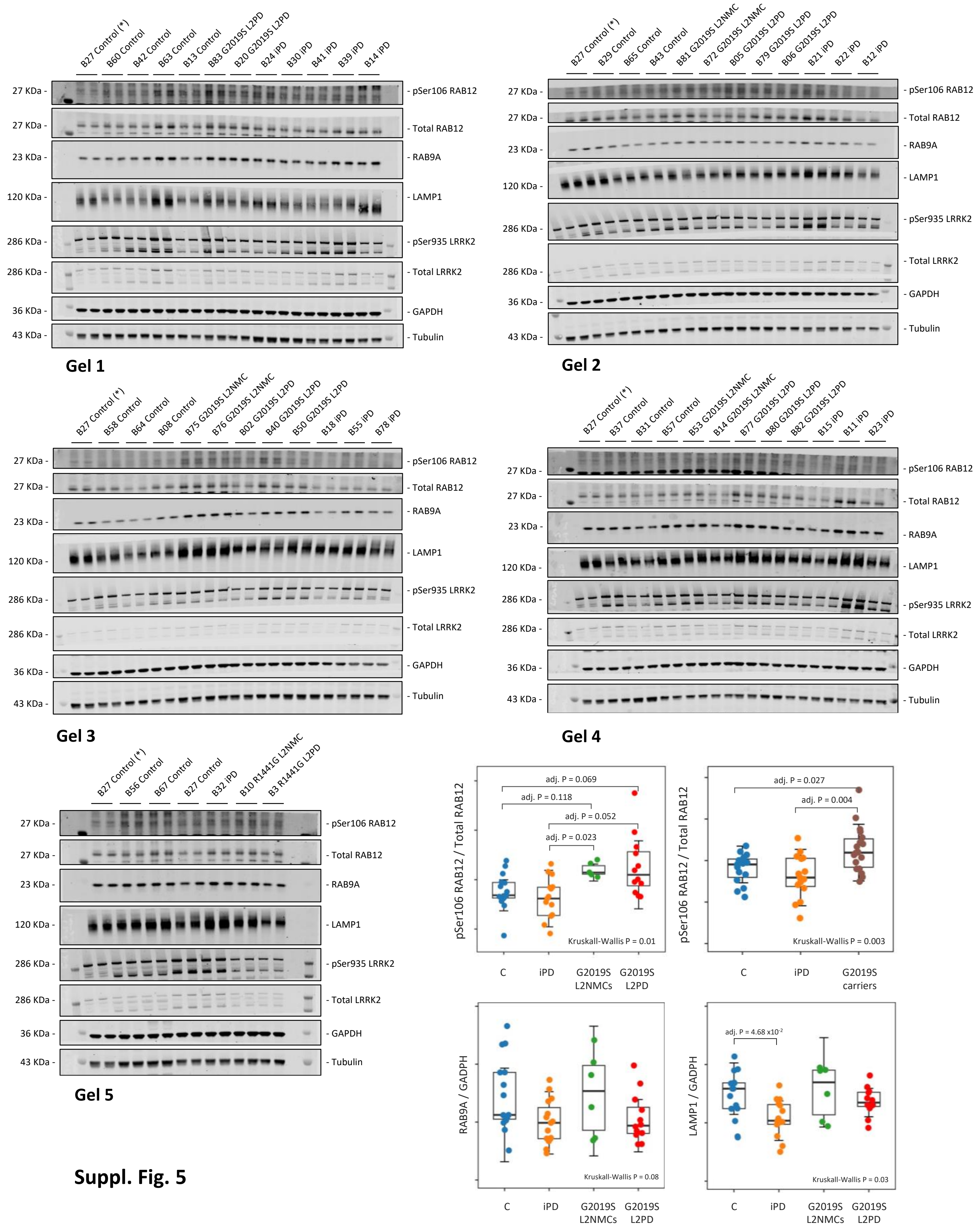

Gel 1
B27 Control (*)
B60 Control
B42 Control
B63 Control
B13 Control
B83 G2019S L2PD
B20 G2019S L2PD
B24 iPD
B30 iPD
B41 iPD
B39 iPD
B14 iPD
Gel 2
B27 Control (*)
B29 Control
B65 Control
B43 Control
B81 G2019S L2NMC
B72 G2019S L2NMC
B05 G2019S L2PD
B79 G2019S L2PD
B06 G2019S L2PD
B21 iPD
B22 iPD
B12 iPD
Gel 3
B27 Control (*)
B58 Control
B64 Control
B08 Control
B75 G2019S L2NMC
B76 G2019S L2NMC
B02 G2019S L2PD
B40 G2019S L2PD
B50 G2019S L2PD
B18 iPD
B55 iPD
B78 iPD
Gel 4
B27 Control (*)
B37 Control
B31 Control
B57 Control
B53 G2019S L2NMC
B14 G2019S L2NMC
B77 G2019S L2PD
B80 G2019S L2PD
B82 G2019S L2PD
B15 iPD
B11 iPD
B23 iPD
Gel 5
B27 Control (*)
B56 Control
B67 Control
B27 Control
B32 iPD
B10 R1441G L2NMC
B3 R1441G L2PD
27 KDa -
23 KDa -
120 KDa -
286 KDa -
36 KDa -
43 KDa -
- pSer106 RAB12
- Total RAB12
- RAB9A
- LAMP1
- pSer935 LRRK2
- Total LRRK2
- GAPDH
- Tubulin
pSer106 RAB12 / Total RAB12
adj. P = 0.069
adj. P = 0.118
adj. P = 0.052
adj. P = 0.023
Kruskall-Wallis P = 0.01
C
iPD
G2019S L2NMCs
G2019S L2PD
adj. P = 0.027
adj. P = 0.004
Kruskall-Wallis P = 0.003
G2019S carriers
RAB9A / GADPH
Kruskall-Wallis P = 0.08
LAMP1 / GADPH
adj. P = 4.68 x10-2
Kruskall-Wallis P = 0.03

Suppl. Fig. 5

Suppl. Fig. 5. Expanded 1-year follow-up of pSer106 RAB12 and other markers by immunoblot

Full immunoblot assessment of pSer106 RAB12 phosphorylation levels, and expression levels of RAB9A and LAMP1, using >1-year follow-up PBMC samples from a subset of the LRRK2 cohort from Clínic-Barcelona (n=48), including G2019S L2PD (n=12), G2019S L2NMCs (n=6), iPD (n=15), and controls (n=15). Dot plots representing normalised levels after the band densitometric analysis for the various studied makers in all subjects studied in duplicates as it follows, pSer106 RAB12 / Total RAB12; RAB9A / GADPH; and LAMP1 / GADPH, all of them double normalised to the same intergel control also measured in duplicates. In each plot, overall intergroup differences were assessed using Kruskal-Wallis test followed by post-hoc Dunn's test under an FDR multiple-testing adjusted $P<0.05$. (*) Denotes intergel control.

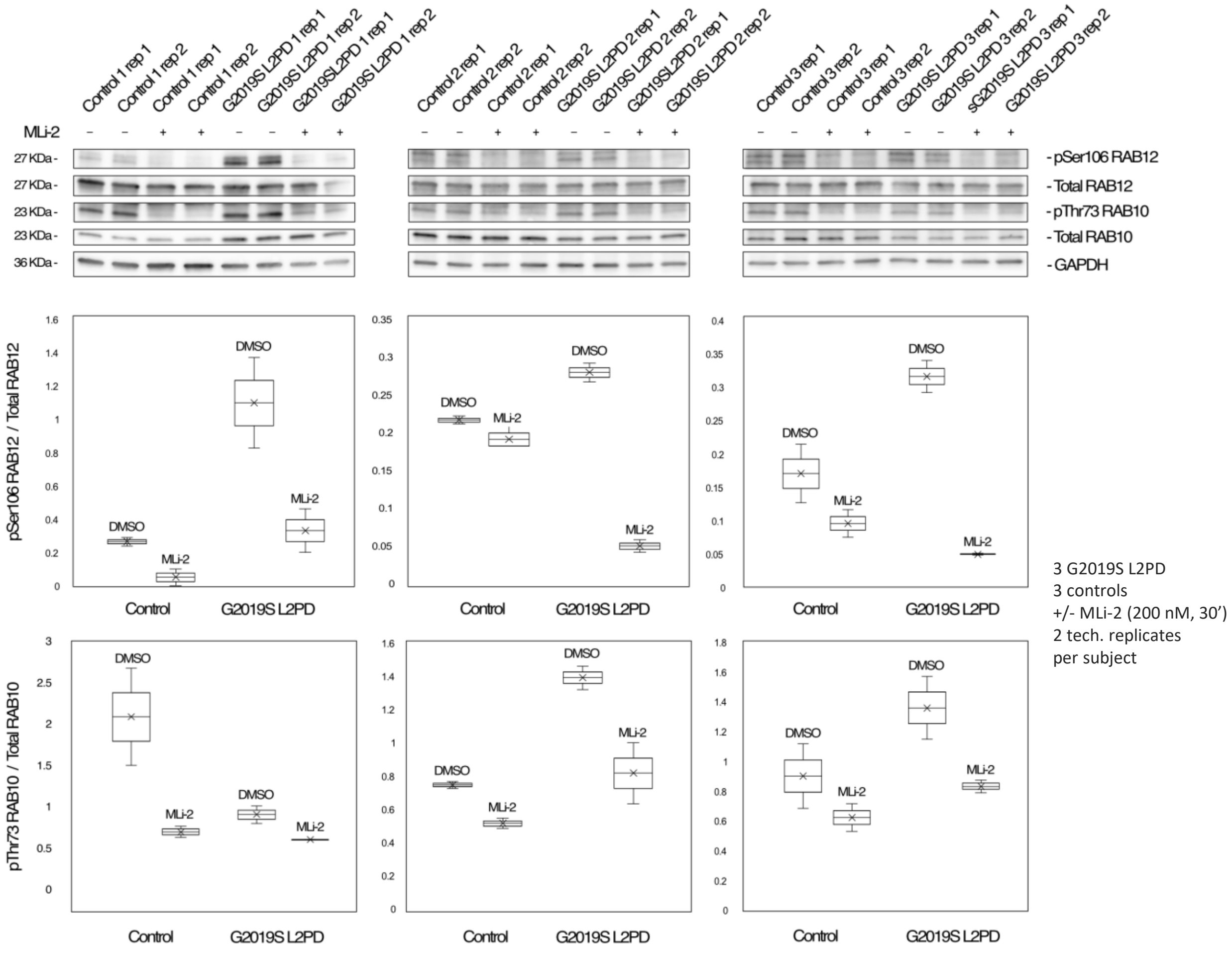

3 G2019S L2PD
3 controls
+/- MLi-2 (200 nM, 30’)
2 tech. replicates
per subject

Suppl. Fig. 6

Suppl. Fig. 6. Expanded pSer106 RAB12 responsiveness to MLi-2 LRRK2 inhibition

Full immunoblot analysis of pSer106 RAB12 / Total RAB12 and pThr73 RAB10 / Total RAB10 using two technical replicates of PBMC lysates from G2019S L2PD (n=3) and healthy controls (n=3), treated with DMSO or the MLi-2 LRRK2 inhibitor (200 nM, 30 min), showing a diminishment of pSer106 RAB12 phosphorylation levels after LRRK2 inhibition by MLi-2 treatment.

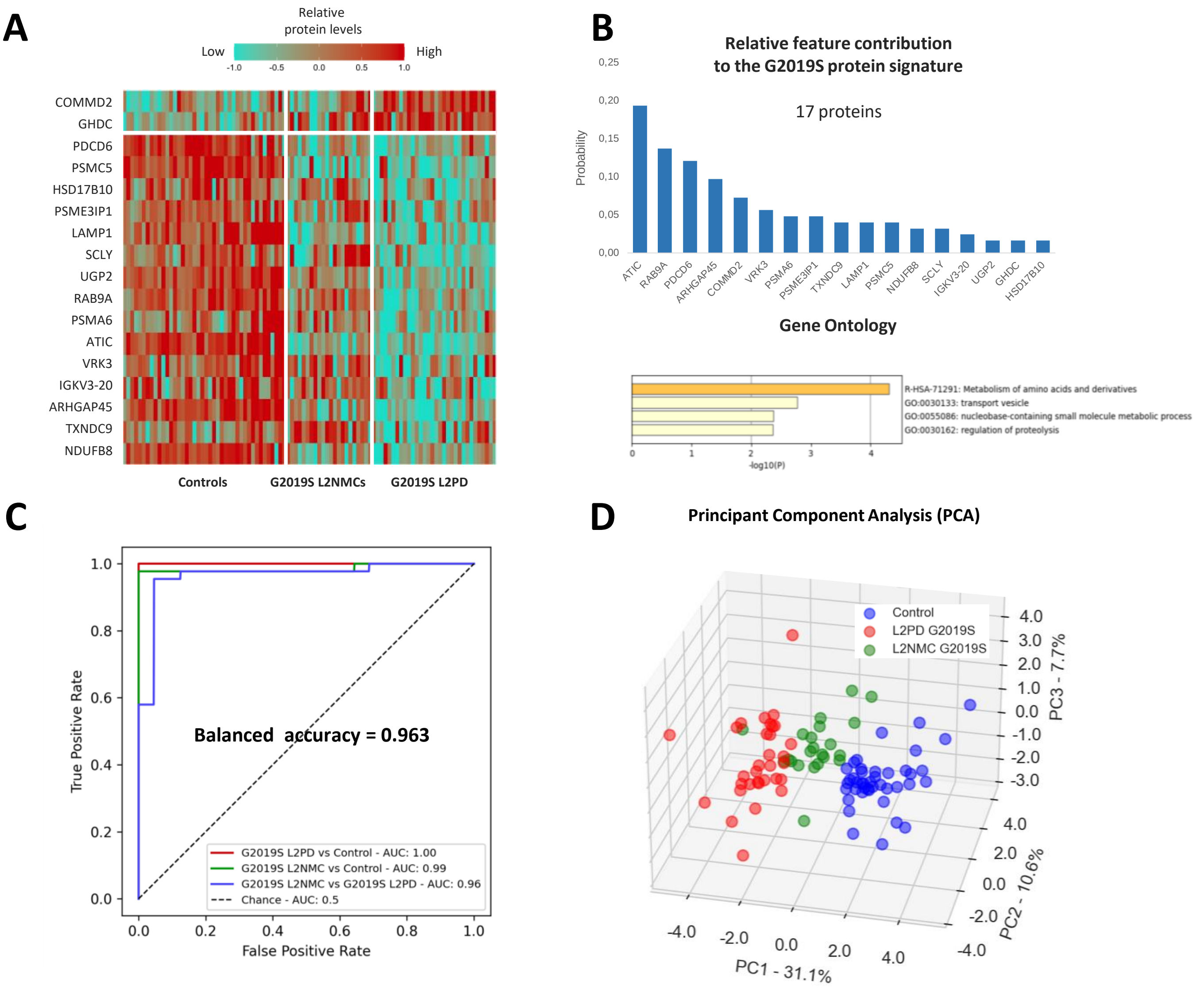
A
Relative protein levels
Low
High
-1.0
-0.5
0.0
0.5
1.0
COMMD2
GHDC
PDCD6
PSMC5
HSD17B10
PSME3IP1
LAMP1
SCLY
UGP2
RAB9A
PSMA6
ATIC
VRK3
IGKV3-20
ARHGAP45
TXNDC9
NDUFB8
Controls
G2019S L2NMCs
G2019S L2PD
B
Relative feature contribution to the G2019S protein signature
17 proteins
Probability
0,25
0,20
0,15
0,10
0,05
0,00
ATIC
RAB9A
PDCD6
ARHGAP45
COMMD2
VRK3
PSMA6
PSME3IP1
TXNDC9
LAMP1
PSMC5
NDUFB8
SCLY
IGKV3-20
UGP2
GHDC
HSD17B10
Gene Ontology
R-HSA-71291: Metabolism of amino acids and derivatives
GO:0030133: transport vesicle
GO:0055086: nucleobase-containing small molecule metabolic process
GO:0030162: regulation of proteolysis
-log10(P)
C
Balanced accuracy = 0.963
True Positive Rate
False Positive Rate
G2019S L2PD vs Control - AUC: 1.00
G2019S L2NMC vs Control - AUC: 0.99
G2019S L2NMC vs G2019S L2PD - AUC: 0.96
Chance - AUC: 0.5
D
Principant Component Analysis (PCA)
Control
L2PD G2019S
L2NMC G2019S
PC1 - 31.1%
PC2 - 10.6%
PC3 - 7.7%

Suppl. Fig. 7

Suppl. Fig. 7. A 17-feature protein classifier for G2019S carriers

To explore alternative classifiers for G2019S carriers and healthy controls, here we applied the same method as described for the 18-feature G2019S phospho-/protein signature, and considered exclusively cross-group differential proteins but not phospho-sites. (**A**) 17-protein G2019S best classifier found in G2019S carriers, symptomatic and asymptomatic, and controls. (**B**) Relative contribution of features from the 17-protein G2019S classifier shown on the upper bar plot; Metascape gene ontology enrichment analysis of the 17-feature G2019S signature displayed on lower bar plot. (**C**) Receiver Operating Curve (ROC) analysis of the 17-protein G2019S signature with a balanced accuracy of 0.963 for discriminating G2019S L2PD, G2019S L2NMCs and controls, with an area under the curve (AUC) of 1.00 between G2019S L2PD and controls, 0.99 between G2019S L2NMCs and controls, and 0.96 between G2019S L2PD and G2019S L2NMCs. (**D**) Principal component analysis (PCA) based on the 17-protein G2019S classifier displaying different subject profiles of G2019S carriers and healthy controls based on LRRK2 mutation and disease status

SUPPLEMENTARY TABLES

Suppl. Table 1. **Performance of each model across phospho-/proteome datasets**

**Proteome**				
Model	Initial (3,816 features)	ANOVA (926 features)	ANOVA + RFECV	Feature optimal Nr.
SVM	0.69 ± 0.09	0.85 ± 0.08	0.91 ± 0.07	510
RF	0.68 ± 0.09	0.70 ± 0.12	0.67 ± 0.08	536
GB	0.69 ± 0.08	0.82 ± 0.13	0.67 ± 0.09	881
**Phospho-proteome**				
Model	Initial (10,180 features)	ANOVA (979 features)	ANOVA + RFECV	Feature optimal Nr.
SVM	0.39 ± 0.11	0.79 ± 0.03	0.95 ± 0.07	204
RF	0.41 ± 0.10	0.53 ± 0.03	0.61 ± 0.10	91
GB	0.40 ± 0.08	0.47 ± 0.07	0.81 ± 0.06	43

SVM = Support Vector Machine; RF = Random Forest; GB = Gradient Boosting

Suppl. Table 2. Confusion matrices for the different classifiers trained on proteome data (first of three parts)

Proteome SVM classifier

	Initial (3,816 features)	Predicted class: Control	L2PD	L2NMC	Total
True class	Control	40	1	1	42
	L2PD	1	23	8	32
	L2NMC	5	11	6	22
	Total	46	35	15	96

	ANOVA (926 features)	Predicted class: Control	L2PD	L2NMC	Total
True class	Control	42	0	0	42
	L2PD	1	28	3	32
	L2NMC	1	7	14	22
	Total	44	35	17	96

	ANOVA + RFECV (510 features)	Predicted class: Control	L2PD	L2NMC	Total
True class	Control	42	0	0	42
	L2PD	0	29	3	32
	L2NMC	0	5	17	22
	Total	42	34	20	96

Proteome RF classifier

	Initial (3,816 features)	Predicted class: Control	L2PD	L2NMC	Total
True class	Control	37	0	5	42
	L2PD	1	23	8	32
	L2NMC	6	6	10	22
	Total	44	29	23	96

	ANOVA (926 features)	Predicted class: Control	L2PD	L2NMC	Total
True class	Control	37	0	5	42
	L2PD	3	22	7	32
	L2NMC	6	4	12	22
	Total	46	26	24	96

	ANOVA + RFECV (536 features)	Predicted class: Control	L2PD	L2NMC	Total
True class	Control	37	0	5	42
	L2PD	2	21	9	32
	L2NMC	5	6	11	22
	Total	44	27	25	96

Proteome GB classifier

	Initial (3,816 features)	Predicted class: Control	L2PD	L2NMC	Total
True class	Control	36	3	3	42
	L2PD	2	24	6	32
	L2NMC	5	7	10	22
	Total	43	34	19	96

	ANOVA (926 features)	Predicted class: Control	L2PD	L2NMC	Total
True class	Control	40	0	2	42
	L2PD	1	26	5	32
	L2NMC	2	5	15	22
	Total	43	31	22	96

	ANOVA + RFECV (881 features)	Predicted class: Control	L2PD	L2NMC	Total
True class	Control	37	1	4	42
	L2PD	2	22	8	32
	L2NMC	3	9	10	22
	Total	42	32	22	96

Suppl. Table 2. Confusion matrices for the different classifiers trained on phospho-proteome data (second of three parts)

Phospho-proteome SVM classifier

	Initial (10,180 features)	Predicted class: Control	L2PD	L2NMC	Total
True class	Control	21	14	0	35
	L2PD	22	6	1	29
	L2NMC	11	7	1	19
	Total	54	27	2	83

	ANOVA (979 features)	Predicted class: Control	L2PD	L2NMC	Total
True class	Control	31	3	1	35
	L2PD	4	23	2	29
	L2NMC	5	1	13	19
	Total	40	27	16	83

	ANOVA + RFECV (204 features)	Predicted class: Control	L2PD	L2NMC	Total
True class	Control	35	0	0	35
	L2PD	2	27	0	29
	L2NMC	2	0	17	19
	Total	39	27	17	83

Phospho-proteome RF classifier

	Initial (10,180 features)	Predicted class: Control	L2PD	L2NMC	Total
True class	Control	18	11	6	35
	L2PD	13	10	6	29
	L2NMC	7	5	7	19
	Total	38	26	19	83

	ANOVA (979 features)	Predicted class: Control	L2PD	L2NMC	Total
True class	Control	22	6	7	35
	L2PD	9	14	6	29
	L2NMC	5	5	9	19
	Total	36	25	22	83

	ANOVA + RFECV (91 features)	Predicted class: Control	L2PD	L2NMC	Total
True class	Control	23	6	6	35
	L2PD	4	19	6	29
	L2NMC	3	6	10	19
	Total	30	31	22	83

Phospho-proteome GB classifier

	Initial (10,180 features)	Predicted class: Control	L2PD	L2NMC	Total
True class	Control	15	12	8	35
	L2PD	13	10	6	29
	L2NMC	5	5	9	19
	Total	33	27	23	83

	ANOVA (979 features)	Predicted class: Control	L2PD	L2NMC	Total
True class	Control	26	4	5	35
	L2PD	14	10	5	29
	L2NMC	6	7	6	19
	Total	46	21	16	83

	ANOVA + RFECV (43 features)	Predicted class: Control	L2PD	L2NMC	Total
True class	Control	31	2	2	35
	L2PD	1	25	3	29
	L2NMC	3	3	13	19
	Total	35	30	18	83

Suppl. Table 2. **Confusion matrices for the different classifiers trained on signatures data (third of three parts)**

18-feature phospho-/protein signature

		Predicted class			
		Control	L2PD	L2NMC	Total
True class	Control	35	0	0	35
	L2PD	0	28	1	29
	L2NMC	0	1	18	19
	Total	35	29	19	83

17-feature protein signature

		Predicted class			
		Control	L2PD	L2NMC	Total
True class	Control	42	0	0	42
	L2PD	0	31	1	32
	L2NMC	1	1	20	22
	Total	43	32	21	96

Suppl. Table 3. Primary antibodies list

Antibody Description	Manufacturer	Cat. Nr.	Dilution / Concentration
Mouse monoclonal GAPDH (6C5)	Santa Cruz	#sc-32233	1:10,000
Rabbit monoclonal LAMP1 (D2D11)	Cell Signalling Tech.	#9091	1:1,000
Mouse monoclonal Total LRRK2	In house generated	-	1:1,000
Rabbit pSer935 LRRK2 [UDD2 10(12)]	Abcam	#ab133450	1:1,000
Rabbit monoclonal RAB9A (D52G8)	Cell Signalling Tech.	#5118	1:1,000
Mouse monoclonal RAB10	Merck	#SAB5300028	1:1,000
Rabbit recombinant pThr73 RAB10 (MJF-R21)	Abcam	#ab230261	1:1,000
Sheep polyclonal Total RAB12	MRC-PPU Reagents and Services at the University of Dundee	#SA227	1 µg/ml
Rabbit monoclonal pSer106 RAB12 (MJF-R25-9)	Abcam	#ab256487	1:1,000
Mouse monoclonal Tubulin (DM1A)	Cell Signalling Tech	#3873S	1:1,000

SUPPLEMENT

R scripts for differential phospho-proteomic analyses, available also as cloud weblink (doi.org/10.5281/zenodo.13774022).

Modified PTM collapsing script: collapsing.pdf

Differential analysis:

- Total Proteomics:
  - Comparison of S mutant (L2PD+L2NMC) samples with Control/iPD: Total_proteome.S_mutant.Differential.Analysis.pdf
  - Comparison of S of each condition (L2PD or L2NMC or iPD) against iPD or Control: Total_proteome.S.Differential.Analysis.pdf
  - Comparison of B mutant (L2PD+L2NMC) samples with Control/iPD: Total_proteome.B_mutant.Differential.Analysis.pdf
  - Comparison of B of each condition (L2PD or L2NMC or iPD) against iPD or Control: Total_proteome.B.Differential.Analysis.pdf
  - Comparison of D mutant (L2PD+L2NMC) samples with Control/iPD: Total_proteome.D_mutant.Differential.Analysis.pdf
  - Comparison of D of each condition (L2PD or L2NMC or iPD) against iPD or Control: Total_proteome.D.Differential.Analysis.pdf
  - Comparison of all mutant across the entire B, S, D geolocation with Control/iPD: Total_proteome.Meta.Mutant.Differential.Analysis.pdf
  - Comparison of each condition (L2PD or L2NMC or iPD) for each mutant across the entire B, S, D geolocation against iPD or Control: Total_proteome.Meta.Differential.Analysis.pdf
- Phospho-proteomics:
  - Comparison of S mutant (L2PD+L2NMC) samples with Control/iPD: Phospho_proteome.S.mutant.pdf
  - Comparison of S of each condition (L2PD or L2NMC or iPD) against iPD or Control: Phospho_proteome.S.pdf
  - Comparison of B mutant (L2PD+L2NMC) samples with Control/iPD: Phospho_proteome.B.mutant.pdf

- Comparison of B of each condition (L2PD or L2NMC or iPD) against iPD or Control: Phospho_proteome.B.pdf
- Comparison of D of each condition (L2PD or L2NMC or iPD) against iPD or Control: Phospho_proteome.D.pdf
- Comparison of all mutant across the entire B, S, D geolocation with Control/iPD: Phospho_proteome..meta.mutant.pdf
- Comparison of each condition (L2PD or L2NMC or iPD) for each mutant across the entire B, S, D geolocation against iPD or Control: Phospho_proteome.meta.pdf
- Comparison of each mutant across the entire B, S, D geolocation with Control/iPD: Phospho_proteome.carriers.pdf

ANOVA

- ANOVA analysis of normalized total proteomics data: anova.pdf
- ANOVA analysis of normalized phospho-proteomics data: anova.phospho.pdf

Spectronaut Export Filter Parameters:

Export summary

Number of rows: 5738141

Filters used:

1. decoyFilter
2. qValueFilter

Key acronyms:

L2PD = LRRK2-associated PD patients

L2NMC = Non-manifesting LRRK2 mutation carriers

iPD = Idiopathic PD patients

G2019S = LRRK2 G2019S mutation

R1441G = LRRK2 R1441G mutation

B = geolocation Barcelona

S = geolocation Santander

D = geolocation Donostia (San Sebastian)

SUMMARY OF LRRK2 PHOSPHO-/PROTEOMIC FINDINGS

Curtain weblinks of phospho-/proteomic findings by disease status, LRRK2 mutation and centre.

Proteome entire G2019S cohort
all G2019S L2PD vs controls
all G2019S carriers vs controls
all G2019S L2NMCs vs controls
all G2019S L2NMCs vs L2PD
all iPD vs controls
all G2019S L2PD vs iPD
all G2019S carriers vs iPD

Phospho entire G2019S cohort
all G2019S L2PD vs controls
all G2019S carriers vs controls
all G2019S L2NMCs vs controls
all G2019S L2NMCs vs L2PD
all iPD vs controls
all G2019S L2PD vs iPD
all G2019S carriers vs iPD

Proteome Barcelona G2019S cohort
Barcelona G2019S L2PD vs controls
Barcelona G2019S carriers vs controls
Barcelona G2019S L2NMCs vs controls
Barcelona G2019S L2NMCs vs L2PD
Barcelona iPD vs controls
Barcelona G2019S L2PD vs iPD
Barcelona G2019S carriers vs iPD

Phospho Barcelona G2019S cohort
Barcelona G2019S L2PD vs controls
Barcelona G2019S carriers vs controls
Barcelona G2019S L2NMCs vs controls
Barcelona G2019S L2NMCs vs L2PD
Barcelona iPD vs controls
Barcelona G2019S L2PD vs iPD
Barcelona G2019S carriers vs iPD

Proteome Santander G2019S cohort
Santander G2019S L2PD vs controls
Santander G2019S carriers vs controls
Santander G2019S L2NMCs vs controls
Santander G2019S L2NMCs vs L2PD
Santander iPD vs controls
Santander G2019S L2PD vs iPD
Santander G2019S carriers vs iPD

Phospho Santander G2019S cohort
Santander G2019S L2PD vs controls
Santander G2019S carriers vs controls
Santander G2019S L2NMCs vs controls
Santander G2019S L2NMCs vs L2PD
Santander iPD vs controls
Santander G2019S L2PD vs iPD
Santander G2019S carriers vs iPD

Proteome San Sebastian R1441G cohort
San Sebastian R1441G L2PD vs controls
San Sebastian R1441G carriers vs controls
San Sebastian R1441G L2NMCs vs controls
San Sebastian R1441G L2NMCs vs L2PD
San Sebastian iPD vs controls
San Sebastian R1441G L2PD vs iPD
San Sebastian R1441G carriers vs iPD

Phospho San Sebastian R1441G cohort
San Sebastian R1441G L2PD vs controls
San Sebastian R1441G carriers vs controls
San Sebastian R1441G L2NMCs vs controls
San Sebastian R1441G L2NMCs vs L2PD
San Sebastian iPD vs controls
San Sebastian R1441G L2PD vs iPD
San Sebastian R1441G carriers vs iPD

```
inFile <- "E:/20230713_143813_22_phospho_no-norm_Report.tsv"
outFile <- "E:/20230713_143813_22_phospho_no-norm_Report.output.tsv"

ptm.string <- "[Phospho (STY)];[Deamidation (NQ)];[Oxidation (M)];[Carbamidomethyl
(C)]" main.col <- "EG.TotalQuantity..Settings."

csv.file <- read.table(inFile, header=TRUE, sep="\t", fill = TRUE )

#installing packages if not installed yet
options(repos='http://cran.rstudio.com/') #without this option, packages cannot be installed
on a fresh R installation
list.of.packages <- c("PerseusR", "data.table", "XML", "doParallel", "stringr",
"MASS", "BiocManager", "Rcpp")
new.packages <- list.of.packages[!(list.of.packages %in%
installed.packages()[,"Package"])] if(length(new.packages))
install.packages(new.packages)

#load packages
suppressMessages(library(data.table))
suppressMessages(library(XML))
suppressMessages(library(doParallel))
suppressMessages(library(stringr))
suppressMessages(library(MASS))
suppressMessages(library(BiocManager))

#install Biostrings for FASTA readin
list.of.packages <- c("Biostrings", "Biobase")
new.packages <- list.of.packages[!(list.of.packages %in%
installed.packages()[,"Package"])] if(length(new.packages))
BiocManager::install(new.packages, ask = F)

#load Biostrings
suppressMessages(library(Biostrings))
suppressMessages(library(PerseusR)) #requires Biobase to work properly


#str(parameters)
#Perseus Data Read
mdata <- csv.file
paramFile <- xmlParse("C:/Users/Toan Phung/Downloads/parameters.xml")
parameters <- xmlToList(paramFile)
#extract parameters from xml file
#condition column renaming; 0 = grouping by condition column, 1 = wide-format no
grouping par.cond <- as.integer(parameters[[1]][[1]][[1]])
if(par.cond == 0){
  #read out column name
  par.cond.col <- unlist(parameters[[1]][[1]][[3]][[1]][[1]][[1]][[2]])
 [as.integer(parameters[[1]][[1]][[3]][[1]][[1]][[1]][[1]])+1]

  #rewrite column name using . for spaces and brackets
  par.cond.col <- gsub("( |\\(|\\)|\\[|\\])", ".", par.cond.col, perl=T)
 }
# if(par.cond == 1){
# #no additional input read out
# }

#collapse level; 0 = localized site level, 1 = localized peptide level (stoich), 2 =
ModSpec peptide level
par.level <- as.integer(parameters[[1]][[2]][[1]])
#read out info for 0 = localized site level
if(par.level == 0){
```

```
  #read out probability column type; 0 = EG.PTMLocalizationProbabilities, 1
= EG.PTMAssayProbability, 2 = No probability column
  par.level.col <- as.integer(parameters[[1]][[2]][[3]][[1]][[1]][[1]][[1]])

  if(par.level.col == 0){
    par.level.cutoff <-
as.numeric(parameters[[1]][[2]][[3]][[1]][[1]][[1]][[3]][[1]][[1]][[1]] [[1]])
  }
  if(par.level.col == 1){
    par.level.cutoff <-
as.numeric(parameters[[1]][[2]][[3]][[1]][[1]][[1]][[3]][[2]][[1]][[1]] [[1]])
  }
  if(par.level.col == 2){
    # par.level.col.nocut <-
unlist(parameters[[1]][[2]][[3]][[1]][[1]][[1]][[3]][[3]][[1]] [[1]][[2]])[
    # as.integer(parameters[[1]][[2]][[3]][[1]][[1]][[1]][[3]][[3]][[1]][[1]][[1]])+1]
par.level.col.nocut <- as.integer(parameters[[1]][[2]][[3]][[1]][[1]][[1]][[3]][[3]][[1]]
[[1]][[1]])
  }

  #read out site level column type; 0 = PG.Genes, 1 = PG.ProteinGroups
  par.level.col.genprot <-
unlist(parameters[[1]][[2]][[3]][[1]][[1]][[2]][[2]])
[as.integer(parameters[[1]][[2]][[3]][[1]][[1]][[2]][[1]])+1]

  #read out FASTA file path
  par.FASTA.file <- gsub("\\", "/", parameters[[1]][[2]][[3]][[1]][[1]][[3]][[1]], fixed=T)

  #read out FASTA parsing rule
  par.FASTA.parse <- parameters[[1]][[2]][[3]][[1]][[1]][[4]][[1]]
}

#read out info for 1 = localized peptide level (stoich)
if(par.level == 1){
  #read out probability column type; 0 = EG.PTMLocalizationProbabilities, 1
= EG.PTMAssayProbability, 2 = No probability column
  par.level.col <- as.integer(parameters[[1]][[2]][[3]][[2]][[1]][[1]][[1]])

  if(par.level.col == 0){
    par.level.cutoff <-
as.numeric(parameters[[1]][[2]][[3]][[2]][[1]][[1]][[3]][[1]][[1]][[1]] [[1]])
  }
  if(par.level.col == 1){
    par.level.cutoff <-
as.numeric(parameters[[1]][[2]][[3]][[2]][[1]][[1]][[3]][[2]][[1]][[1]] [[1]])
  }
  if(par.level.col == 2){
    # par.level.col.nocut <-
unlist(parameters[[1]][[2]][[3]][[2]][[1]][[1]][[3]][[3]][[1]] [[1]][[2]])[
    # as.integer(parameters[[1]][[2]][[3]][[2]][[1]][[1]][[3]][[3]][[1]][[1]][[1]])+1]
par.level.col.nocut <- as.integer(parameters[[1]][[2]][[3]][[2]][[1]][[1]][[3]][[3]][[1]]
[[1]][[1]])
  }
  if(par.level.col == 3){
    #MaxQuant modified sequence
    par.level.cutoff <-
as.numeric(parameters[[1]][[2]][[3]][[2]][[1]][[1]][[3]][[4]][[1]][[1]] [[1]])

    #read out MQ probability column, only used for this specific setup
    par.level.MQ.col <-
unlist(parameters[[1]][[2]][[3]][[2]][[1]][[1]][[3]][[4]][[1]][[2]] [[2]])[
```

```
      as.integer(parameters[[1]][[2]][[3]][[2]][[1]][[1]][[3]][[4]][[1]][[2]][[1]])+1
    ] #rewrite column name using . for spaces and brackets
    par.level.MQ.col <- gsub("( |\\(|\\)|\\[|\\])", ".", par.level.MQ.col,
  perl=T) }

  #read out stoichiometry settings; 0 = Calculate stoichiometries, 1 = Skip
 par.level.stoich <- as.integer(parameters[[1]][[2]][[3]][[2]][[1]][[2]][[1]])
 }

 #read out info for 2 = ModSpec peptide level
 if(par.level == 2){
  #read out probability column type; 0 = EG.PTMLocalizationProbabilities, 1
 = EG.PTMAssayProbability, 2 = No probability column
  par.level.col <- as.integer(parameters[[1]][[2]][[3]][[3]][[1]][[1]][[1]])
  if(par.level.col == 0){
    par.level.cutoff <-
 as.numeric(parameters[[1]][[2]][[3]][[3]][[1]][[1]][[3]][[1]][[1]][[1]] [[1]])
  }
  if(par.level.col == 1){
    par.level.cutoff <-
 as.numeric(parameters[[1]][[2]][[3]][[3]][[1]][[1]][[3]][[2]][[1]][[1]] [[1]])
  }
  if(par.level.col == 2){
    # par.level.col.nocut <-
 unlist(parameters[[1]][[2]][[3]][[3]][[1]][[1]][[3]][[3]][[1]] [[1]][[2]])[
    # as.integer(parameters[[1]][[2]][[3]][[3]][[1]][[1]][[3]][[3]][[1]][[1]][[1]])+1]
 par.level.col.nocut <- as.integer(parameters[[1]][[2]][[3]][[3]][[1]][[1]][[3]][[3]][[1]]
 [[1]][[1]])
  }
 }

 #read out PTM strings
 par.PTM <- unlist(strsplit(parameters[[1]][[3]][[1]], split=";"))

 #read out aggregation type; 0 = Linear modeling based, 1 = summing
 par.agg <- as.integer(parameters[[1]][[4]][[1]])

 #read out number of CPU threads
 par.CPU <- as.integer(parameters[[1]][[5]][[1]])



 ## START DATA PROCESSING

 #transform main columns and annotation column into data.table object
 data <- setDT(mdata)
 #data <- data[, .SD[1:1000,], by=par.cond.col]


 #check if condition column contains at least 2 distinct conditions
 if(par.cond == 0L){
  if(data[, length(unique(get(par.cond.col)))<2]){
    stop("Condition column contains less than 2 distinct conditions, please select a
 different condition column or renaming parameters")
  }
 }

 #check if necessary columns present
 if(par.level==0){
  #need PEP.PeptidePosition
  if(!"PEP.PeptidePosition" %in% names(data)){
```

```
    stop("Missing: site-level consolidation requires PEP.PeptidePosition column to
  work") }

  #need PG.Genes
  if(par.level.col.genprot==0 & !"PG.Genes" %in% names(data)){
    stop("Missing: site-level consolidation with chosen settings requires PG.Genes column
to work")
  }

  #need PG.ProteinGroups
  if(par.level.col.genprot==1 & !"PG.ProteinGroups" %in% names(data)){
    stop("Missing: site-level consolidation with chosen settings requires
PG.ProteinGroups column to work")
  }
}

#if MQ modified sequence cutoff set, check if at least 1 "Probability" column
provided if(par.level == 1L & par.level.col == 3L){
  if( length(grep("Probabilities$", names(data), perl=T)) == 0L){
  stop("When probability filtering with MaxQuant modified sequence input, please provide at
least 1 ... Probabilities column")
  }
}


# setnames(data, "EG.TotalQuantity..Settings.", "Int1")
# data[, Int2 := Int1*2]
# main.name <- c("Int1", "Int2")
# data[, R.FileName := gsub("20171125_QE7_nLC14_DBJ_SA_DIAphos_RPE1_pilot2_",
"DIA_", R.FileName, fixed=T)]


#extract main column names
main.name <- main.col
cols <- colnames(data)
else.name <- cols[cols!=main.name]

# #stop function if more than 1 main column provided
# if(length(main.name)>1){
# stop("Only 1 main column may be provided for long-to-wide format transformation")
# }

#delete "Filtered", "0" and "1" entries, then reload as numeric columns
for(col in main.name){
  data[get(col)=="Filtered", paste(col) := NA]
  data[get(col)==1, paste(col) := NA]
  data[get(col)==0, paste(col) := NA]
  data[get(col)=="NaN", paste(col) := NA]
  data[, paste(col) := as.numeric(get(col))]
}
rm(col)



#correct EG.PTMLocProb and/or extract PTM_0 pos/probs from
EG.PTMLocProb/EG.PrecId #initiate multiple cores
cl <- makeCluster(par.CPU)
registerDoParallel(cl)

#for data testing only
#temp <- copy(data)
```

```
#PTM <- par.PTM[1]

if(par.level == 0){

  if(any(par.level.col == 0, if(exists("par.level.col.nocut")){par.level.col.nocut == 0},
na.rm = T)){

    #if par.level = 0 & (par.level.col = 0 OR par.level.col.nocut = 0) ->
EG.PTMLocProb-based: correct EG.PrecId & extract PTM_0 positions/probs

    #define function
    PTM.valid.extract.site.string <- function(temp, par.PTM){

      PTM.count <- 0
      for(PTM in par.PTM){
        #remove square brackets from PTM and mark round brackets as escaped, then insert
into pattern
        pat <- gsub("[^:]*", gsub(")", "\\)", gsub("(", "\\(", gsub("(\\[|\\])", "",
PTM, perl=T), fixed=T), fixed=T), "\\[[^:]*\\: (.{1,5})\\%\\]", fixed=T)
        pat.del <- gsub("[^:]*", gsub(")", "\\)", gsub("(", "\\(", gsub("(\\[|\\])", "",
PTM, perl=T), fixed=T), fixed=T), "\\[[^:]*\\: .{1,5}\\%\\]", fixed=T)

        #create PTM valid row vector
        PTM.exist <- paste("PTM_", PTM.count, "_exist", sep="")
        temp[, paste(PTM.exist) :=F]
        temp[like(EG.PTMLocalizationProbabilities, pat), paste(PTM.exist) :=T]
        #use pattern to extract total number of respective PTM probabilities summed
        up temp[, paste("PTM_", PTM.count, "_num", sep="") :=
               sapply(EG.PTMLocalizationProbabilities, function(x){
round(sum(as.numeric(gsub(pat, "\\1", unlist(str_extract_all(x, pat)), perl=T)))/100, 0) })]

        #use pattern to extract respective PTM probabilities
        temp[get(PTM.exist), paste("PTM_", PTM.count, "_prob", sep="") :=
               sapply(EG.PTMLocalizationProbabilities, function(x){
as.numeric(gsub(pat, "\\1", unlist(str_extract_all(x, pat)), perl=T))/100 })]

        #create EG.PTMLocalizationProbabilities with other PTMs deleted
        temp[get(PTM.exist), paste("PTM_", PTM.count, "_seq", sep="") :=
EG.PTMLocalizationProbabilities]
        for(PTM.other in setdiff(par.PTM, PTM)){
          pat.other <- gsub("[^:]*", gsub(")", "\\)", gsub("(", "\\(", gsub("(\\[|\\])",
"", PTM.other, perl=T), fixed=T), fixed=T), "\\[[^:]*\\: .{1,5}\\%\\]", fixed=T) temp[,
paste(paste("PTM_", PTM.count, "_seq", sep="")) := gsub(pat.other, "", get(paste("PTM_",
PTM.count, "_seq", sep="")), perl=T)]
        }

        #extract positions of respective PTMs
        temp[get(PTM.exist), paste("PTM_", PTM.count, "_pos", sep="") :=
sapply(get(paste("PTM_", PTM.count, "_seq", sep="")), function(x){
          #problem: if PTM on last/first position can create exemption if "_" removed -> use
as anchor to mark start/stop of sequence and then substract
          temp <- nchar(unlist(strsplit(x, split=pat.del)))
          temp <- temp-c(1, rep(0, length(temp)-2), 1)
          return(cumsum(temp[-length(temp)]))
        })]
        #extract PTM valid localization vector: eg T, T, F, T, F
        temp[get(PTM.exist), paste("PTM_", PTM.count, "_log", sep="") :=
               mapply(function(x, y){ rank(-x, ties.method = "first")<=y },
                      x=get(paste("PTM_", PTM.count, "_prob", sep="")), y=get(paste("PTM_",
PTM.count, "_num", sep="")))]
```

```
        #extract PTM valid positions: eg 3,8,15
        temp[get(PTM.exist), paste("PTM_", PTM.count, "_pos_val", sep="") :=
               mapply(function(x, y){ list(x[y]) }, x=get(paste("PTM_", PTM.count,
"_pos", sep="")), y=get(paste("PTM_", PTM.count, "_log", sep="")))]

        #extract PTM valid probabilities: eg 1,1,1
        temp[get(PTM.exist), paste("PTM_", PTM.count, "_prob_val", sep="") :=
               mapply(function(x, y){ list(x[y]) }, x=get(paste("PTM_", PTM.count,
"_prob", sep="")), y=get(paste("PTM_", PTM.count, "_log", sep="")))]

        #increase PTM counter
        PTM.count <- PTM.count+1
      }

      #create base sequence column
      temp[, PTM_base_seq := gsub("\\[[^:]*\\: .{1,5}\\%\\]", "", gsub("_",
"", EG.PTMLocalizationProbabilities, fixed=T), perl=T)]
      #create summed PTM num column
      temp[, PTM_num := apply(.SD, 1, function(x){ sum(x, na.rm=T) }), .SDcols
= grep("^PTM_.*_num$", names(temp), value=T, perl=T)]
      #create combined PTM position column
      temp[PTM_num>0, PTM_pos_val := apply(.SD, 1, function(x){ unlist(x) }), .SDcols
= grep("^PTM_.*_pos_val$", names(temp), value=T, perl=T)]
      #create combined PTM type column
      temp[PTM_num>0, PTM_type_val := list(apply(.SD, 1, function(x){
unlist(mapply(function(y, z){rep(z, y)}, y=x, z=par.PTM)) })),
           .SDcols = grep("^PTM_.*_num$", names(temp), value=T, perl=T)]
      #create sorted combined PTM position column
      temp[PTM_num>0, PTM_pos_val_sorted := lapply(PTM_pos_val, sort)]
      #create sorted combined PTM type column
      temp[PTM_num>0, PTM_type_val_sorted := mapply(function(x, y){ x[order(y)] },
x=PTM_type_val, y=PTM_pos_val)]

      #recreate EG.PrecursorId column with valid types only
      temp[PTM_num==0, EG.PrecursorId.PTM.val := PTM_base_seq]
      temp[PTM_num>0, EG.PrecursorId.PTM.val := mapply(function(posit, types,
        baseseq){ #step by step create res character string
        res <- character()
        pos.save <- 0
        #iterate for loop over each individual pos/type
        for(i in 1:(length(posit)) ){
          #build res from base sequence until pos
          res <- paste(res, substr(baseseq, start=pos.save, stop=posit[i]),
          sep="") #insert PTM type
          res <- paste(res, types[i], sep="")
          #update pos.save
          pos.save <- posit[i]+1
        }
        #finish by attaching last sequence substring
        return(paste(res, substr(baseseq, start=pos.save, stop=nchar(baseseq)),
      sep="")) }, posit=PTM_pos_val_sorted, types=PTM_type_val_sorted,
      baseseq=PTM_base_seq)]

      #create column combined with charge state for dcast
      temp[, PTM_group := paste("_", EG.PrecursorId.PTM.val, "_", gsub("_.*_(\\..)$",
"\\1", EG.PrecursorId, perl=T), sep="")]

      #delete all rows except PTM_0 and PTM_base_seq (needed for aa type
extraction) for(col in setdiff(grep("^PTM_[1-9][0-9]*_.*$", names(temp), value=T,
perl=T), c("PTM_base_seq", "PTM_group"))){
```

```
        temp[, paste(col) := NULL]
      }

      #rename probability column
      setnames(temp, "PTM_0_prob_val", "PTM_localization")

      #return data table to top level
      return(temp)
    }

    #filter out all rows NOT containing target PTM (-> useless for site-level collapsing)
pat <- gsub("[^:]*", gsub(")", "\\)", gsub("(", "\\(", gsub("(\\[|\\])", "", par.PTM[1],
perl=T), fixed=T), fixed=T), "\\[[^:]*\\: (.{1,5})\\%\\]", fixed=T)

    if(data[!like(EG.PTMLocalizationProbabilities, pat), .N]>0){
      print(paste("Cave: Removed ", data[!like(EG.PTMLocalizationProbabilities, pat),
                  .N], " rows not containin target PTM in
                  EG.PTMLocalizationProbabilities",
sep=""))
    }
    data <- copy(data[like(EG.PTMLocalizationProbabilities, pat), ])
    rm(pat)

    #divide dataset into sub-datasets for parallelization
    data[, subd := sort(c(rep((1:(par.CPU-1)), round((.N/par.CPU), 0)), rep(par.CPU,
.N- (round((.N/par.CPU), 0)*(par.CPU-1)) )))]
    data.sub <- split(data, by="subd")

    data <- copy(rbindlist(foreach(testp=data.sub, .packages=c("data.table",
"stringr")) %dopar% {
      PTM.valid.extract.site.string(temp=testp, par.PTM=par.PTM)
    }))
    print("Done: EG.PTMLocalizationProbabilities-based EG.PrecursorId correction and
PTM position/probability extraction")
    #temp[PTM_0_num>0, .(PTM_0_pos_val, PTM_localization,
EG.PrecursorId.PTM.val, EG.PrecursorId.PTM.val.charge)]

  } else if(any(par.level.col == 1, if(exists("par.level.col.nocut")){par.level.col.nocut
== 1}, na.rm = T)){
    #if par.level = 0 & (par.level.col = 1 OR par.level.col.nocut = 1) -> EG.PrecId-based:
extract PTM_0 positions

    #define function
    PTM.valid.extract.site.string <- function(temp, par.PTM){

      #extract num of PTM 0 from EG.PrecId
      temp[, PTM_0_num := sapply(EG.PrecursorId, function(x){
length(unlist(strsplit(x, split=par.PTM[1], fixed=T)))-1 })]

      #create base sequence column, and PTM 0 only seq column
      temp[, PTM_base_seq := gsub("^(.*)\\..$", "\\1", gsub("\\[[^[]*\\]", "", gsub("_",
"", EG.PrecursorId, fixed=T), perl=T), perl=T)]
      #define deletion pattern for all PTMs except target PTM
      pat.del.all <- paste("\\[(?!", gsub(")", "\\)", gsub("(", "\\(", substr(par.PTM[1],
start = 2, stop = nchar(par.PTM[1])-1), fixed=T), fixed=T),
                          ")[^[]*\\]", sep="")
      temp[, PTM_0_seq := gsub(pat.del.all, "", gsub("^(.*)\\..$", "\\1",
EG.PrecursorId, perl=T), perl=T)]

      #create PTM_0_pos_val
      temp[PTM_0_num>0, PTM_0_pos_val := sapply(PTM_0_seq, function(x){
        #problem: if PTM on last/first position can create exemption if "_" removed -> use
```

```
as anchor to mark start/stop of sequence and then substract
        tempo <- nchar(unlist(strsplit(x, split=par.PTM[1], fixed=T)))
        tempo <- tempo-c(1, rep(0, length(tempo)-2), 1)
        return(cumsum(tempo[-length(tempo)]))
      })]

      #delete PTM_0_seq
      temp[, PTM_0_seq := NULL]

      #create PTM group
      temp[, PTM_group := EG.PrecursorId]

      #return data table to top level
      return(temp)
    }

    #filter out all rows NOT containing target PTM (-> useless for site-level
collapsing) pat <- gsub("(", "\\(", gsub(")", "\\)", gsub("[", "\\[", gsub("]", "\\]",
par.PTM[1], fixed=T), fixed=T), fixed=T), fixed=T)
    if(data[!like(EG.PrecursorId, pat), .N]>0){
      print(paste("Cave: Removed ", data[!like(EG.PrecursorId, pat), .N],
                  " rows not containin target PTM in EG.PTMLocalizationProbabilities",
sep=""))
    }
    data <- data[like(EG.PrecursorId, pat), ]
    rm(pat)

    #divide dataset into sub-datasets for parallelization
    data[, subd := sort(c(rep((1:(par.CPU-1)), round((.N/par.CPU), 0)), rep(par.CPU,
.N- (round((.N/par.CPU), 0)*(par.CPU-1)) )))]
    data.sub <- split(data, by="subd")

    data <- copy(rbindlist(foreach(testp=data.sub, .packages=c("data.table",
"stringr")) %dopar% {
      PTM.valid.extract.site.string(temp=testp, par.PTM=par.PTM)
    }))
    print("Done: EG.PrecursorId-based PTM position extraction")
    #temp[PTM_0_num>0, .(PTM_0_pos_val)]

  }
} else
  if(par.level == 1){

    if(any(par.level.col == 0, if(exists("par.level.col.nocut")){par.level.col.nocut ==
0}, na.rm = T)){
      #if par.level = 1 & (par.level.col = 0 OR par.level.col.nocut = 0) ->
EG.PTMLocProb based: correct EG.PrecId

      #define function
      PTM.valid.extract.site.string <- function(temp, par.PTM){

        PTM.count <- 0
        for(PTM in par.PTM){
          #remove square brackets from PTM and mark round brackets as escaped, then insert
into pattern
          pat <- gsub("[^:]*", gsub(")", "\\)", gsub("(", "\\(", gsub("(\\[|\\])", "",
PTM, perl=T), fixed=T), fixed=T), "\\[[^:]*\\: (.{1,5})\\%\\]", fixed=T)
          pat.del <- gsub("[^:]*", gsub(")", "\\)", gsub("(", "\\(", gsub("(\\[|\\])", "",
PTM, perl=T), fixed=T), fixed=T), "\\[[^:]*\\: .{1,5}\\%\\]", fixed=T)

          #create PTM valid row vector
```

```
        PTM.exist <- paste("PTM_", PTM.count, "_exist", sep="")
        temp[, paste(PTM.exist) :=F]
        temp[like(EG.PTMLocalizationProbabilities, pat), paste(PTM.exist) :=T]

        #use pattern to extract total number of respective PTM probabilities summed
        up temp[, paste("PTM_", PTM.count, "_num", sep="") :=
               sapply(EG.PTMLocalizationProbabilities, function(x){
round(sum(as.numeric(gsub(pat, "\\1", unlist(str_extract_all(x, pat)), perl=T)))/100, 0) })]

        #use pattern to extract respective PTM probabilities
        temp[get(PTM.exist), paste("PTM_", PTM.count, "_prob", sep="") :=
               sapply(EG.PTMLocalizationProbabilities, function(x){
as.numeric(gsub(pat, "\\1", unlist(str_extract_all(x, pat)), perl=T))/100 })]

        #create EG.PTMLocalizationProbabilities with other PTMs deleted
        temp[get(PTM.exist), paste("PTM_", PTM.count, "_seq", sep="") :=
EG.PTMLocalizationProbabilities]
        for(PTM.other in setdiff(par.PTM, PTM)){
          pat.other <- gsub("[^:]*", gsub(")", "\\)", gsub("(", "\\(", gsub("(\\[|\\])",
"", PTM.other, perl=T), fixed=T), fixed=T), "\\[[^:]*\\: .{1,5}\\%\\]", fixed=T) temp[,
paste(paste("PTM_", PTM.count, "_seq", sep="")) := gsub(pat.other, "", get(paste("PTM_",
PTM.count, "_seq", sep="")), perl=T)]
        }

        #extract positions of respective PTMs
        temp[get(PTM.exist), paste("PTM_", PTM.count, "_pos", sep="") :=
sapply(get(paste("PTM_", PTM.count, "_seq", sep="")), function(x){
          #problem: if PTM on last/first position can create exemption if "_" removed ->
use as anchor to mark start/stop of sequence and then substract
          temp <- nchar(unlist(strsplit(x, split=pat.del)))
          temp <- temp-c(1, rep(0, length(temp)-2), 1)
          return(cumsum(temp[-length(temp)]))
        })]
        #extract PTM valid localization vector: eg T, T, F, T, F
        temp[get(PTM.exist), paste("PTM_", PTM.count, "_log", sep="") :=
              mapply(function(x, y){ rank(-x, ties.method = "first")<=y },
                     x=get(paste("PTM_", PTM.count, "_prob", sep="")), y=get(paste("PTM_",
PTM.count, "_num", sep="")))]

        #extract PTM valid positions: eg 3,8,15
        temp[get(PTM.exist), paste("PTM_", PTM.count, "_pos_val", sep="") :=
              mapply(function(x, y){ list(x[y]) }, x=get(paste("PTM_", PTM.count,
"_pos", sep="")), y=get(paste("PTM_", PTM.count, "_log", sep="")))]

        #extract PTM valid probabilities: eg 1,1,1
        temp[get(PTM.exist), paste("PTM_", PTM.count, "_prob_val", sep="") :=
              mapply(function(x, y){ list(x[y]) }, x=get(paste("PTM_", PTM.count,
"_prob", sep="")), y=get(paste("PTM_", PTM.count, "_log", sep="")))]

        #increase PTM counter
        PTM.count <- PTM.count+1
      }


      #create base sequence column
      temp[, PTM_base_seq := gsub("\\[[^:]*\\: .{1,5}\\%\\]", "", gsub("_",
"", EG.PTMLocalizationProbabilities, fixed=T), perl=T)]
      #create summed PTM num column
      temp[, PTM_num := apply(.SD, 1, function(x){ sum(x, na.rm=T) }), .SDcols
= grep("^PTM_.*_num$", names(temp), value=T, perl=T)]
      #create combined PTM position column
```

```
        temp[PTM_num>0, PTM_pos_val := apply(.SD, 1, function(x){ unlist(x) }), .SDcols
= grep("^PTM_.*_pos_val$", names(temp), value=T, perl=T)]
        #create combined PTM type column
        temp[PTM_num>0, PTM_type_val := list(apply(.SD, 1, function(x){
unlist(mapply(function(y, z){rep(z, y)}, y=x, z=par.PTM)) })),
             .SDcols = grep("^PTM_.*_num$", names(temp), value=T, perl=T)]
        #create sorted combined PTM position column
        temp[PTM_num>0, PTM_pos_val_sorted := lapply(PTM_pos_val, sort)]
        #create sorted combined PTM type column
        temp[PTM_num>0, PTM_type_val_sorted := mapply(function(x, y){ x[order(y)]
}, x=PTM_type_val, y=PTM_pos_val)]

        #recreate EG.PrecursorId column with valid types only
        temp[PTM_num==0, EG.PrecursorId.PTM.val := PTM_base_seq]
        temp[PTM_num>0, EG.PrecursorId.PTM.val := mapply(function(posit, types,
          baseseq){ #step by step create res character string
          res <- character()
          pos.save <- 0
          #iterate for loop over each individual pos/type
          for(i in 1:(length(posit)) ){
            #build res from base sequence until pos
            res <- paste(res, substr(baseseq, start=pos.save, stop=posit[i]),
            sep="") #insert PTM type
            res <- paste(res, types[i], sep="")
            #update pos.save
            pos.save <- posit[i]+1
          }
          #finish by attaching last sequence substring
          return(paste(res, substr(baseseq, start=pos.save, stop=nchar(baseseq)),
        sep="")) }, posit=PTM_pos_val_sorted, types=PTM_type_val_sorted,
        baseseq=PTM_base_seq)]

        #create column combined with charge state for dcast
        temp[, PTM_group := paste("_", EG.PrecursorId.PTM.val, "_", gsub("_.*_(\\..)$",
"\\1", EG.PrecursorId, perl=T), sep="")]

        #calculate mean peptide probability column -> target PTM probabilities
        only temp[, PTM_localization := NA_real_]
        #temp[PTM_num>0, PTM_localization := apply(.SD, 1, function(x)
{mean(as.numeric(unlist(x)), na.rm=T)}), .SDcols = grep("^PTM_.*_prob_val$",
names(temp), value=T)]
        temp[PTM_0_num>0, PTM_localization := sapply(PTM_0_prob_val,
function(x) {mean(as.numeric(unlist(x)), na.rm=T)})]

        #delete all rows except PTM_0 and PTM_base_seq (needed for aa type
extraction) for(col in setdiff(grep("^PTM_[1-9][0-9]*_.*$", names(temp), value=T,
perl=T), c("PTM_base_seq", "PTM_localization", "PTM_group"))){
          temp[, paste(col) := NULL]
        }

        #return data table to top level
        return(temp)
      }

      #divide dataset into sub-datasets for parallelization
      data[, subd := sort(c(rep((1:(par.CPU-1)), round((.N/par.CPU), 0)), rep(par.CPU,
.N- (round((.N/par.CPU), 0)*(par.CPU-1)) )))]
      data.sub <- split(data, by="subd")
      data <- copy(rbindlist(foreach(testp=data.sub, .packages=c("data.table",
"stringr")) %dopar% {
        PTM.valid.extract.site.string(temp=testp, par.PTM=par.PTM)
```

```
      }))
      print("Done: EG.PTMLocalizationProbabilities-based EG.PrecursorId
correction") #temp[PTM_0_num>0, .(PTM_0_pos_val, PTM_localization,
EG.PrecursorId.PTM.val, EG.PrecursorId.PTM.val.charge)]

    } else if(any(par.level.col == 1, if(exists("par.level.col.nocut")){par.level.col.nocut
== 1}, na.rm = T)){
      #if par.level = 1 & (par.level.col = 1 OR par.level.col.nocut = 1) -> nothing (use
raw EG.PrecId later)

      #define function
      PTM.valid.extract.site.string <- function(temp, par.PTM){

        #extract number of PTM
        temp[, paste("PTM_0_num", sep="") := sapply(EG.PrecursorId,
function(x){ length(unlist(strsplit(x, split=par.PTM[1], fixed=T)))-1 })]

        #define PTM_group
        temp[, PTM_group := EG.PrecursorId]

        #return data table to top level
        return(temp)
      }

      #divide dataset into sub-datasets for parallelization
      data[, subd := sort(c(rep((1:(par.CPU-1)), round((.N/par.CPU), 0)), rep(par.CPU,
.N- (round((.N/par.CPU), 0)*(par.CPU-1)) )))]
      data.sub <- split(data, by="subd")

      data <- copy(rbindlist(foreach(testp=data.sub, .packages=c("data.table",
"stringr")) %dopar% {
        PTM.valid.extract.site.string(temp=testp, par.PTM=par.PTM)
      }))
      print("Done: EG.PrecursorId-based PTM number extraction")

    } else if(if(exists("par.level.col.nocut")){par.level.col.nocut ==
      2}else{F}){ #if MQ Modified.sequence without probability column

      #define function
      PTM.valid.extract.site.string <- function(temp, par.PTM){

        #extract number of PTM
        temp[, paste("PTM_0_num", sep="") := sapply(Modified.sequence,
function(x){ length(unlist(strsplit(x, split=par.PTM[1], fixed=T)))-1 })]

        #define PTM_group
        temp[, PTM_group := Modified.sequence]

        #return data table to top level
        return(temp)
      }

      #divide dataset into sub-datasets for parallelization
      data[, subd := sort(c(rep((1:(par.CPU-1)), round((.N/par.CPU), 0)), rep(par.CPU,
.N- (round((.N/par.CPU), 0)*(par.CPU-1)) )))]
      data.sub <- split(data, by="subd")

      data <- copy(rbindlist(foreach(testp=data.sub, .packages=c("data.table",
"stringr")) %dopar% {
        PTM.valid.extract.site.string(temp=testp, par.PTM=par.PTM)
      }))
      print("Done: EG.PrecursorId-based PTM number extraction")
```

```
    } else if(par.level.col == 3L){
      #if MQ Modified.sequence AND probability extraction

      #define function
      PTM.valid.extract.site.string <- function(temp, par.PTM){

        #extract number of PTM
        temp[, paste("PTM_0_num", sep="") := sapply(Modified.sequence,
function(x){ length(unlist(strsplit(x, split=par.PTM[1], fixed=T)))-1 })]

        #define PTM_group
        temp[, PTM_group := Modified.sequence]

        #create PTM valid row vector
        temp[, PTM_0_exist := F]
        temp[like(get(par.level.MQ.col), "\\("), PTM_0_exist :=T]

        #use pattern to extract respective PTM probabilities
        temp[PTM_0_exist==T, PTM_0_prob :=
               sapply(get(par.level.MQ.col), function(x){ as.numeric(gsub("\\((.*)\\)",
"\\1", unlist(str_extract_all(x, "\\([^(]*\\)")), perl=T)) })]

        #extract positions of respective PTMs
        temp[PTM_0_exist==T, PTM_0_pos := sapply(get(par.level.MQ.col), function(x){
#problem: if PTM on last/first position can create exemption if "_" removed -> use as anchor
to mark start/stop of sequence and then substract
          temp <- nchar(unlist(strsplit(paste("_", x, "_", sep=""),
          split="\\([^(]*\\)"))) temp <- temp-c(1, rep(0, length(temp)-2), 1)
          return(cumsum(temp[-length(temp)]))
        })]

        #extract PTM valid localization vector: eg T, T, F, T, F
        temp[PTM_0_exist==T, PTM_0_log := mapply(function(x, y){ rank(-x, ties.method
= "first")<=y }, x=PTM_0_prob, y=PTM_0_num)]

        #extract PTM valid positions: eg 3,8,15
        temp[PTM_0_exist==T, PTM_0_pos_val := mapply(function(x, y){ list(x[y]) },
x=PTM_0_pos, y=PTM_0_log)]

        #extract PTM valid probabilities: eg 1,1,1
        temp[PTM_0_exist==T, PTM_0_prob_val := mapply(function(x, y){ list(x[y])
}, x=PTM_0_prob, y=PTM_0_log)]

        #calculate mean peptide probability column -> target PTM probabilities
        only temp[, PTM_localization := NA_real_]
        #temp[PTM_num>0, PTM_localization := apply(.SD, 1, function(x)
{mean(as.numeric(unlist(x)), na.rm=T)}), .SDcols = grep("^PTM_.*_prob_val$",
names(temp), value=T)]
        temp[PTM_0_num>0, PTM_localization := sapply(PTM_0_prob_val,
function(x) {mean(as.numeric(unlist(x)), na.rm=T)})]

        #return data table to top level
        return(temp)
      }

      #divide dataset into sub-datasets for parallelization
      data[, subd := sort(c(rep((1:(par.CPU-1)), round((.N/par.CPU), 0)), rep(par.CPU,
.N- (round((.N/par.CPU), 0)*(par.CPU-1)) )))]
      data.sub <- split(data, by="subd")

      data <- copy(rbindlist(foreach(testp=data.sub, .packages=c("data.table",
"stringr")) %dopar% {
```

```
        PTM.valid.extract.site.string(temp=testp, par.PTM=par.PTM)
      }))
      print("Done: EG.PrecursorId-based PTM number extraction")

    }
  } else
    if(par.level == 2){

      if(any(par.level.col == 0, if(exists("par.level.col.nocut")){par.level.col.nocut ==
0}, na.rm = T)){
        #if par.level = 2 & (par.level.col = 0 OR par.level.col.nocut = 0) -> extract
num, type and prob from EG.PTMLocProb

        #define function
        PTM.valid.extract.site.string <- function(temp, par.PTM){

          PTM.count <- 0
          for(PTM in par.PTM){

            #remove square brackets from PTM and mark round brackets as escaped, then
insert into pattern
            pat <- gsub("[^:]*", gsub(")", "\\)", gsub("(", "\\(", gsub("(\\[|\\])", "",
PTM, perl=T), fixed=T), fixed=T), "\\[[^:]*\\: (.{1,5})\\%\\]", fixed=T)
            pat.del <- gsub("[^:]*", gsub(")", "\\)", gsub("(", "\\(", gsub("(\\[|\\])",
"", PTM, perl=T), fixed=T), fixed=T), "\\[[^:]*\\: .{1,5}\\%\\]", fixed=T)

            #create PTM valid row vector
            PTM.exist <- paste("PTM_", PTM.count, "_exist", sep="")
            temp[, paste(PTM.exist) :=F]
            temp[like(EG.PTMLocalizationProbabilities, pat), paste(PTM.exist) :=T]

            #use pattern to extract total number of respective PTM probabilities summed
            up temp[, paste("PTM_", PTM.count, "_num", sep="") :=
                    sapply(EG.PTMLocalizationProbabilities, function(x){
round(sum(as.numeric(gsub(pat, "\\1", unlist(str_extract_all(x, pat)), perl=T)))/100, 0) })]

            #use pattern to extract respective PTM probabilities
            temp[get(PTM.exist), paste("PTM_", PTM.count, "_prob", sep="") :=
                    sapply(EG.PTMLocalizationProbabilities, function(x){
as.numeric(gsub(pat, "\\1", unlist(str_extract_all(x, pat)), perl=T))/100 })]

            #create EG.PTMLocalizationProbabilities with other PTMs deleted
            temp[get(PTM.exist), paste("PTM_", PTM.count, "_seq", sep="") :=
EG.PTMLocalizationProbabilities]
            for(PTM.other in setdiff(par.PTM, PTM)){
              pat.other <- gsub("[^:]*", gsub(")", "\\)", gsub("(", "\\(",
gsub("(\\[|\\])", "", PTM.other, perl=T), fixed=T), fixed=T), "\\[[^:]*\\: .{1,5}\\%\\]",
fixed=T) temp[, paste(paste("PTM_", PTM.count, "_seq", sep="")) := gsub(pat.other, "",
get(paste("PTM_", PTM.count, "_seq", sep="")), perl=T)]
            }

            #extract positions of respective PTMs
            temp[get(PTM.exist), paste("PTM_", PTM.count, "_pos", sep="") :=
sapply(get(paste("PTM_", PTM.count, "_seq", sep="")), function(x){
              #problem: if PTM on last/first position can create exemption if "_" removed
-> use as anchor to mark start/stop of sequence and then substract
              temp <- nchar(unlist(strsplit(x, split=pat.del)))
              temp <- temp-c(1, rep(0, length(temp)-2), 1)
              return(cumsum(temp[-length(temp)]))
            })]
            #extract PTM valid localization vector: eg T, T, F, T, F
```

```
            temp[get(PTM.exist), paste("PTM_", PTM.count, "_log", sep="") :=
                   mapply(function(x, y){ rank(-x, ties.method = "first")<=y },
                          x=get(paste("PTM_", PTM.count, "_prob", sep="")),
y=get(paste("PTM_", PTM.count, "_num", sep="")))]

            #extract PTM valid positions: eg 3,8,15
            temp[get(PTM.exist), paste("PTM_", PTM.count, "_pos_val", sep="") :=
                   mapply(function(x, y){ list(x[y]) }, x=get(paste("PTM_", PTM.count,
"_pos", sep="")), y=get(paste("PTM_", PTM.count, "_log", sep="")))]
            #extract PTM valid probabilities: eg 1,1,1
            temp[get(PTM.exist), paste("PTM_", PTM.count, "_prob_val", sep="") :=
                   mapply(function(x, y){ list(x[y]) }, x=get(paste("PTM_", PTM.count,
"_prob", sep="")), y=get(paste("PTM_", PTM.count, "_log", sep="")))]


            #increase PTM counter
            PTM.count <- PTM.count+1
          }

          #create base sequence column from EG.PTMLocProb
          temp[, PTM_base_seq := gsub("\\[[^:]*\\: .{1,5}\\%\\]", "", gsub("_",
"", EG.PTMLocalizationProbabilities, fixed=T), perl=T)]

          #create summed PTM num column
          temp[, PTM_num := apply(.SD, 1, function(x){ sum(x, na.rm=T) }), .SDcols
= grep("^PTM_.*_num$", names(temp), value=T, perl=T)]

          #create MQ modification specific peptides-like sequence modification
column temp[, PTM_collapse_key := paste(PTM_base_seq, apply(.SD, 1, function(x){
paste(x[x>0], par.PTM[x>0], collapse=",", sep="") }), sep="_"),
                .SDcols = grep("^PTM_.*_num$", names(temp), value=T)]

          #calculate mean peptide probability column
          temp[, PTM_localization := NA_real_]
          #temp[PTM_num>0, PTM_localization := apply(.SD, 1, function(x)
{mean(as.numeric(unlist(x)), na.rm=T)}), .SDcols = grep("^PTM_.*_prob_val$",
names(temp), value=T)]
          temp[PTM_0_num>0, PTM_localization := sapply(PTM_0_prob_val,
function(x) {mean(as.numeric(unlist(x)), na.rm=T)})]


          #create combined PTM position column
          temp[PTM_num>0, PTM_pos_val := apply(.SD, 1, function(x){ unlist(x) }), .SDcols
= grep("^PTM_.*_pos_val$", names(temp), value=T, perl=T)]
          #create combined PTM type column
          temp[PTM_num>0, PTM_type_val := list(apply(.SD, 1, function(x){
unlist(mapply(function(y, z){rep(z, y)}, y=x, z=par.PTM)) })),
                .SDcols = grep("^PTM_.*_num$", names(temp), value=T, perl=T)]
          #create sorted combined PTM position column
          temp[PTM_num>0, PTM_pos_val_sorted := lapply(PTM_pos_val, sort)]
          #create sorted combined PTM type column
          temp[PTM_num>0, PTM_type_val_sorted := mapply(function(x, y){ x[order(y)]
}, x=PTM_type_val, y=PTM_pos_val)]

          #recreate EG.PrecursorId column with valid types only
          temp[PTM_num==0, EG.PrecursorId.PTM.val := PTM_base_seq]
          temp[PTM_num>0, EG.PrecursorId.PTM.val := mapply(function(posit, types,
            baseseq){ #step by step create res character string
            res <- character()
            pos.save <- 0
            #iterate for loop over each individual pos/type
```

```
            for(i in 1:(length(posit)) ){
              #build res from base sequence until pos
              res <- paste(res, substr(baseseq, start=pos.save, stop=posit[i]),
              sep="") #insert PTM type
              res <- paste(res, types[i], sep="")
              #update pos.save
              pos.save <- posit[i]+1
            }
            #finish by attaching last sequence substring
            return(paste(res, substr(baseseq, start=pos.save, stop=nchar(baseseq)),
          sep="")) }, posit=PTM_pos_val_sorted, types=PTM_type_val_sorted,
          baseseq=PTM_base_seq)]

          #create column combined with charge state for dcast
          temp[, PTM_group := paste("_", EG.PrecursorId.PTM.val, "_",
gsub("_.*_(\\..)$", "\\1", EG.PrecursorId, perl=T), sep="")]
          #delete all columns except PTM_collapse_key, PTM_group, PTM_localization
and PTM_0_prob_val
          for(col in setdiff(grep("^PTM_.*$", names(temp), value=T, perl=T),
c("PTM_group", "PTM_collapse_key", "PTM_localization", "PTM_0_num"))){
            temp[, paste(col) := NULL]
          }

          #return data table to top level
          return(temp)
        }

        #divide dataset into sub-datasets for parallelization
        data[, subd := sort(c(rep((1:(par.CPU-1)), round((.N/par.CPU), 0)), rep(par.CPU,
.N- (round((.N/par.CPU), 0)*(par.CPU-1)) )))]
        data.sub <- split(data, by="subd")

        data <- copy(rbindlist(foreach(testp=data.sub, .packages=c("data.table",
"stringr")) %dopar% {
          PTM.valid.extract.site.string(temp=testp, par.PTM=par.PTM)
        }))
        print("Done: EG.PTMLocalizationProbabilities-based PTM
position/probability extraction")
        #temp[PTM_0_num>0, .(PTM_group, PTM_collapse_key, PTM_localization)]

      } else if(par.level == 2 & any(par.level.col == 1,
if(exists("par.level.col.nocut")) {par.level.col.nocut == 1}, na.rm = T)){
        #if par.level = 2 & (par.level.col = 1 OR par.level.col.nocut = 1) -> extract num
and type from EG.PrecId

        #define function
        PTM.valid.extract.site.string <- function(temp, par.PTM){

          PTM.count <- 0
          for(PTM in par.PTM){

            #extract number of PTM
            temp[, paste("PTM_", PTM.count, "_num", sep="") :=
sapply(EG.PrecursorId, function(x){ length(unlist(strsplit(x, split=PTM,
fixed=T)))-1 })]

            #increase PTM counter
            PTM.count <- PTM.count+1
          }

          #create PTM base seq from EG.PrecId
```

```
          temp[, PTM_base_seq := gsub("^(.*)\\..$", "\\1", gsub("\\[[^[]*\\]", "",
gsub("_", "", EG.PrecursorId, fixed=T), perl=T), perl=T)]

          #create MQ modification specific peptides-like sequence modification
column temp[, PTM_collapse_key := paste(PTM_base_seq, apply(.SD, 1, function(x){
paste(x[x>0], par.PTM[x>0], collapse=",", sep="") }), sep="_"),
                .SDcols = grep("^PTM_.*_num$", names(temp), value=T)]

          #delete all rows except PTM_collapse_key
          for(col in setdiff(grep("^PTM_.*$", names(temp), value=T, perl=T),
c("PTM_collapse_key", "PTM_0_num"))){
            temp[, paste(col) := NULL]
          }

          #define PTM_group
          temp[, PTM_group := EG.PrecursorId]

          #return data table to top level
          return(temp)
        }
        #divide dataset into sub-datasets for parallelization
        data[, subd := sort(c(rep((1:(par.CPU-1)), round((.N/par.CPU), 0)), rep(par.CPU,
.N- (round((.N/par.CPU), 0)*(par.CPU-1)) )))]
        data.sub <- split(data, by="subd")

        data <- copy(rbindlist(foreach(testp=data.sub, .packages=c("data.table",
"stringr")) %dopar% {
          PTM.valid.extract.site.string(temp=testp, par.PTM=par.PTM)
        }))
        print("Done: EG.PrecursorId-based PTM position extraction")
        #temp[PTM_0_num>0, .(PTM_group, PTM_collapse_key)]

      }
    }

#finish parallel processing
stopCluster(cl)
data[, subd := NULL]




#site-level only: row expansion
if(par.level == 0){

  #expand dataset row-wise
  data[, rown := .I]

  if(any(par.level.col == 0, if(exists("par.level.col.nocut")){par.level.col.nocut == 0},
na.rm = T)){
    #localization column extracted from EG.PTMLocProb
    data.exp <- copy(rbind(data[PTM_0_num>0, .(PTM_localization =
unlist(PTM_localization), PTM_0_pos_val = unlist(PTM_0_pos_val)), by=rown],
                           data[PTM_0_num==0, .(rown, PTM_localization = NA, PTM_0_pos_val =
NA_integer_)])[order(rown)])
    #merge missing data onto dataset, excluding PTM_localization column
    data <- merge(x=data.exp, y=data[, .SD, .SDcols = setdiff(names(data),
c("PTM_localization", "PTM_0_pos_val"))],
                  by.x="rown", by.y="rown", all.x=T, all.y=F)

  } else {
```

```
    #localization column is EG.PTMAssayProbability
    data.exp <- copy(rbind(data[PTM_0_num>0, .(PTM_0_pos_val =
unlist(PTM_0_pos_val)), by=rown],
                           data[PTM_0_num==0, .(rown, PTM_0_pos_val = NA_integer_)])
[order(rown)])
    #merge missing data onto dataset, excluding pSTY.loc.val column
    data <- merge(x=data.exp, y=data[, .SD, .SDcols = setdiff(names(data),
c("PTM_0_pos_val"))],
                  by.x="rown", by.y="rown", all.x=T, all.y=F)
   }

  data[, rown := NULL]

  #create PTM_0_aa column
  data[, PTM_0_aa := substr(PTM_base_seq, start=PTM_0_pos_val, stop=PTM_0_pos_val)]

  print("Done: site-level expansion")
}


#PTM-localization formatting
if(par.level.col %in% c(0, 3)){
  data[, PTM_localization := as.numeric(PTM_localization)]
}
if(par.level.col == 1){
  data[, PTM_localization := as.numeric(EG.PTMAssayProbability)]
}



#transform data from long into wide format based on condition column
if(par.cond==0L){

  #create cond.names column -> if more than 1 main column, equals combination of main.name
and cond.col arguments
  if(length(main.name)>1){
    cond.names <- as.vector(outer(main.name, data[, sort(unique(get(par.cond.col)))],
paste, sep="_"))
   } else {
    #if only 1 column, equals cond.col arguments
    cond.names <- data[, sort(unique(get(par.cond.col)))]
   }

  #site-level and probability included
  if(par.level == 0 & par.level.col %in% c(0,1,3)){

    #create vector of all user defined columns to keep during transformation -> PTM_group
and co excluded; PTM_0_num and PTM_0_aa included for multiplicity tag
    #keep <- c(setdiff(names(data), c(grep("^PTM_.*$", names(data), value=T,
perl=T), par.cond.col, main.name) ), "PTM_0_num", "PTM_0_aa")
    keep <- c(setdiff(else.name, par.cond.col), "PTM_0_num", "PTM_0_aa")

    #perform transformation via dcast
    data <- cbind(dcast(data, PTM_group + PTM_0_pos_val ~ get(par.cond.col), value.var
= main.name,
                        fun.aggregate = function(x) if(sum(is.na(x)) == length(x))
{NA_real_}else{sum(x, na.rm = T)}), #sum only if >0 non-NA
                  dcast(data, PTM_group + PTM_0_pos_val ~ ., value.var = keep,
                        fun.aggregate = function(x)
    paste(unique(unlist(strsplit(as.character(x), split=";", fixed=T))), collapse=";"))[,
                -c(1,2)], dcast(data, PTM_group + PTM_0_pos_val ~ ., value.var =
```

```
c("PTM_localization"),
                         fun.aggregate = function(x) if(sum(is.na(x)) == length(x))
{NA_real_}else{max(x, na.rm=T)})[, -c(1,2)]) #max only if >0 non-NA
    setnames(data, ".", "PTM_localization")

    print("Done: Long- to wide-format transformation")
  }
  #site-level and probability ignored
  if(par.level == 0 & par.level.col == 2){

    #create vector of all user defined columns to keep during transformation -> PTM_group
and co excluded; PTM_0_num and PTM_0_aa included for multiplicity tag
    #keep <- c(setdiff(names(data), c(grep("^PTM_.*$", names(data), value=T,
perl=T), par.cond.col, main.name) ), "PTM_0_num", "PTM_0_aa")
    keep <- c(setdiff(else.name, par.cond.col), "PTM_0_num", "PTM_0_aa")

    #perform transformation via dcast
    data <- cbind(dcast(data, PTM_group + PTM_0_pos_val ~ get(par.cond.col), value.var
= main.name,
                         fun.aggregate = function(x) if(sum(is.na(x)) == length(x))
{NA_real_}else{sum(x, na.rm = T)}), #sum only if >0 non-NA
                   dcast(data, PTM_group + PTM_0_pos_val ~ ., value.var = keep,
                         fun.aggregate = function(x)
    paste(unique(unlist(strsplit(as.character(x), split=";", fixed=T))), collapse=";"))[,
                                              -c(1,2)])

    print("Done: Long- to wide-format transformation")
  }

  #localized/ModSpec peptide-level and probability included
  if(par.level %in% c(1,2) & par.level.col %in% c(0,1,3)){

    #for ModSpec only, include PTM_collapse_key in keep -> should always be unique
    if(par.level==1){
      #create vector of all user defined columns to keep during transformation -> PTM_group
and co excluded; PTM_0_num included
      #keep <- c(setdiff(names(data), c(grep("^PTM_.*$", names(data), value=T,
perl=T), par.cond.col, main.name) ))
      keep <- c(setdiff(else.name, par.cond.col), "PTM_0_num")
    }
    if(par.level==2){
      #create vector of all user defined columns to keep during transformation -> PTM_group
and co excluded; PTM_0_num included
      #keep <- c(setdiff(names(data), c(grep("^PTM_.*$", names(data), value=T,
perl=T), par.cond.col, main.name) ), "PTM_collapse_key")
      keep <- c(setdiff(else.name, par.cond.col), "PTM_0_num",
    "PTM_collapse_key") }

    #perform transformation via dcast
    data <- cbind(dcast(data, PTM_group ~ get(par.cond.col), value.var =
                         main.name, fun.aggregate = function(x) if(sum(is.na(x)) ==
                         length(x))
{NA_real_}else{sum(x, na.rm = T)}), #sum only if >0 non-NA
                   dcast(data, PTM_group ~ ., value.var = keep,
                         fun.aggregate = function(x)
   paste(unique(unlist(strsplit(as.character(x), split=";", fixed=T))), collapse=";"))[,
          -c(1)], dcast(data, PTM_group ~ ., value.var = c("PTM_localization"),
                         fun.aggregate = function(x) if(sum(is.na(x)) == length(x))
{NA_real_}else{max(x, na.rm=T)})[, -c(1)]) #max only if >0 non-NA
    setnames(data, ".", "PTM_localization")
```

```
    print("Done: Long- to wide-format transformation")
  }
  #localized/ModSpec peptide-level and probability ignored
  if(par.level %in% c(1,2) & par.level.col == 2){

    #for ModSpec only, include PTM_collapse_key in keep -> should always be
    unique if(par.level==1){
      #create vector of all user defined columns to keep during transformation -> PTM_group
and co excluded; PTM_0_num and PTM_0_aa included for multiplicity tag
      #keep <- c(setdiff(names(data), c(grep("^PTM_.*$", names(data), value=T,
perl=T), par.cond.col, main.name) ))
      keep <- c(setdiff(else.name, par.cond.col), "PTM_0_num")
    }
    if(par.level==2){
      #create vector of all user defined columns to keep during transformation -> PTM_group
and co excluded; PTM_0_num and PTM_0_aa included for multiplicity tag
      #keep <- c(setdiff(names(data), c(grep("^PTM_.*$", names(data), value=T,
perl=T), par.cond.col, main.name) ), "PTM_collapse_key")
      keep <- c(setdiff(else.name, par.cond.col), "PTM_0_num",
    "PTM_collapse_key") }

    #perform transformation via dcast
    data <- cbind(dcast(data, PTM_group ~ get(par.cond.col), value.var =
                        main.name, fun.aggregate = function(x) if(sum(is.na(x)) ==
                        length(x))
{NA_real_}else{sum(x, na.rm = T)}), #sum only if >0 non-NA
                  dcast(data, PTM_group ~ ., value.var = keep,
                        fun.aggregate = function(x)
paste(unique(unlist(strsplit(as.character(x), split=";", fixed=T))), collapse=";"))[, -c(1)])

    print("Done: Long- to wide-format transformation")
  }
} else {
  #create cond.names column which equals main.name only
  cond.names <- main.name
}




#prepare consolidation
#delete all non-target PTM rows? -> leave that to manual Perseus pre-filtering; already
done for site-level only
#site-level preparation -> key on PTM_mult123_tag
if(par.level==0){
  #create gene/protein column based on user defined gene/protein column -> ignore first
entry if ""
  data[, PTM_genprot := sapply(get(par.level.col.genprot), function(x){
    strsplit(x, split="(;|,)", perl=T)[[1]][!strsplit(x, split="(;|,)", perl=T)[[1]] ==
   ""][1] })]

  #remove rows that are empty in genprot
  if(data[(is.na(PTM_genprot) | PTM_genprot==""| PTM_genprot=='""'), .N]>0){
print(paste("Cave: Removed ", data[(is.na(PTM_genprot) | PTM_genprot==""|
PTM_genprot=='""'), .N], " rows due to missing gene/prot information",
sep="")) }
  data <- data[!(is.na(PTM_genprot) | PTM_genprot==""| PTM_genprot=='""'), ]

  #create position column based on position column -> ignore first entry if
  "" data[, PTM_pep_pos := sapply(PEP.PeptidePosition, function(x){
    strsplit(x, split="(;|,)", perl=T)[[1]][!strsplit(x, split="(;|,)", perl=T)[[1]] ==
```

```
  ""][1] })]

  #create multiplicity vector, reading out number of target PTM per
  peptides data[, PTM_mult123 := as.integer(PTM_0_num)]
  data[PTM_mult123>=3, PTM_mult123 := 3]
  #PTM_mult123_tag, but renamed to PTM_collapse_key
  data[, PTM_collapse_key := paste(PTM_genprot, "_", PTM_0_aa,
 (PTM_0_pos_val+as.integer(PTM_pep_pos)-1), "_M", PTM_mult123, sep="")]
  setkey(data, "PTM_collapse_key")
 }
#localized peptide-level preparation -> key on localized EG.PrecId without
charge if(par.level==1){
  #create PTM_group without charge state
  data[, PTM_collapse_key := gsub("^(.*)\\..$", "\\1", PTM_group, perl=T)]

  #delete all PTM info except target PTM from collapse key
  for(PTM.other in par.PTM[-1]){
    data[, PTM_collapse_key := gsub(PTM.other, "", PTM_collapse_key,
  fixed=T)] }

  #key on PTM_collapse_key
  setkey(data, "PTM_collapse_key")
 }
#ModSpec peptide-level preparation -> key on PTM_ModSpec
if(par.level==2){
  #key on PTM_collapse_key
  setkey(data, "PTM_collapse_key")
 }




#initiate consolidation
#prepare list of subset data dt, which are passed to the cores
individually if(par.cond == 0L){
  if(par.level.col %in% c(0,1,3)){
    keep <- c(cond.names, setdiff(else.name, par.cond.col), "PTM_0_num",
 "PTM_group", "PTM_collapse_key", "PTM_localization")
   }
  if(par.level.col == 2){
    keep <- c(cond.names, setdiff(else.name, par.cond.col), "PTM_0_num",
 "PTM_group", "PTM_collapse_key")
   }
 } else if(par.cond == 1L){
  if(par.level.col %in% c(0,1,3)){
    keep <- c(cond.names, else.name, "PTM_0_num", "PTM_group",
 "PTM_collapse_key", "PTM_localization")
   }
  if(par.level.col == 2){
    keep <- c(cond.names, else.name, "PTM_0_num", "PTM_group",
  "PTM_collapse_key") }
 }


#create vector with PTM_collapse_key group intervals
sub_vec <- data[, round(quantile(1:length(unique(PTM_collapse_key)), probs =
seq(0,1,length.out = par.CPU+1)[-(par.CPU+1)]),0)]

#create PTM_subgrp column based on sub_vec, which marks groups of rows to be subset into
lists for parallel processing
PTM_subgrp_vec <- data[, .(PTM_grp_nr = rep(.GRP, .N)), by=PTM_collapse_key
```

```
#create PTM_collapse_key group numbers
][, .(PTM_grp_test = c(.BY %in% sub_vec, rep(F, .N-1))), by=PTM_grp_nr #mark each first row
of PTM_grp_nr that matches vec as T
][, cumsum(PTM_grp_test)]
data[, PTM_subgrp := PTM_subgrp_vec] #create PTM_subgrp column

#create subdt: 8 (=number of cores) sub-data tables within list to be passed into
function separately
subdt <- split(data[, .SD, .SDcols = c(keep, "PTM_subgrp")], by="PTM_subgrp")

# #check if total number of rows in subdt fits those in test
# sum(unlist(lapply(subdt, nrow)))
# #check if first and last entry different
# for(i in 1:(par.CPU-1)){
# print(paste(subdt[[i]][.N, .(PTM_collapse_key)]==subdt[[i+1]][1, .(PTM_collapse_key)],
# subdt[[i]][.N, .(PTM_collapse_key)], subdt[[i+1]][1, .(PTM_collapse_key)], sep=" ; "))
# }

#load function: consolidation based on equal division (2018-03-21)
consolidate <- function(cons, cond){
  #check if only NA -> return data table with 1 row filled only with NA
  immediately if(cons[, all(is.na(.SD))]){
    return(as.data.table(matrix(rep(NA, length(cond)), nrow = 1, dimnames = list(NULL,
  cond)))) }

  #sort rows from lowest median intensity to highest, to later make sure that (if
needed), lowest median rows are kicked out first
  cons <- cons[order(cons[, apply(.SD, 1, function(x) median(x, na.rm=T)), .SDcols = cond])]

  #check if consolidation neccessary at all -> check if all conditions have no missing
values while(cons[, any(!sapply(.SD, function(x) length(which(!is.na(x))))[cons[,
sapply(.SD, function(x) length(which(!is.na(x))))]>0]==.N)]){
    #create loop variables for first iteration
    tempc <- NULL

    #start for loop iteration
    for(num in (1:cons[, .N])){
      #check if any NA in this row; if yes, execute normalization, if not simply copy
      values if(any(is.na(unlist(transpose(cons)[, num, with=F])))){
        #create logical vector reading out NA entries
        logv <- is.na(unlist(transpose(cons)[, num, with=F]))

        #this line calculates the missing value substitution values, and then reorders
them together with the non-missing values
        #finally, it adds them into the tempc matrix via rbind
        #need to separate: if >1 NA value, can use apply; but if less then directly use
median instead of apply
        if(length(which(logv))==1){
          #trigger this function if there is exactly 1 NA in this row
         tempc <- rbind(tempc, c(median(sapply(transpose(cons)[, -num, with=F], function(x)
              median(unlist(transpose(cons)[, num, with=F])/x, na.rm=T)*x[logv]), na.rm=T),
                                          unlist(transpose(cons)[, num, with=F], use.names = F)
[!logv])[order(c(which(logv), which(!logv)))])
        } else {
          #trigger this function if there are more then 1 NA in this row
          tempc <- rbind(tempc, c(apply(sapply(transpose(cons)[, -num, with=F],
function(x) median(unlist(transpose(cons)[, num, with=F])/x, na.rm=T)*x[logv]), 1,
                                            function(x) median(x, na.rm=T)),
                                    unlist(transpose(cons)[, num, with=F], use.names = F)
[!logv])[order(c(which(logv), which(!logv)))])
        }
```

```
      } else {
        #copy values instead
        tempc <- rbind(tempc, unlist(transpose(cons)[, num, with=F]))
      }
    }

    #when for loop done, check if tempc matrix equals starting matrix
    if(identical(cons, setNames(as.data.table(tempc), cond))){
      #if identical, means that consolidation does not work anymore -> remove cons row
with lowest number of
      cons <- cons[-(grep(min(apply(cons, 1, function(x)
length(which(!is.na(x))))), apply(cons, 1, function(x)
length(which(!is.na(x)))))[1]), ]

    } else {
      #if consolidation was triggered, then make tempc new cons
      cons <- setNames(as.data.table(tempc), cond)
    }
    #now, check while condition again and if neccessary, rerurn while loop
   }

  #whether or not while loop was triggered, should now have dt without NA except for
completely empty conditions
  #thus, calculate col medians and return (CAVE: we are in intensity space;
CAVE: transposition)
  #option 1: center all rows around 0 in log space, then med, then exponential
transformation; preferrable because no row is over-/under-weighted?
  #return(setNames(cons[, lapply(transpose(lapply(transpose(.SD), function(x)
log(x)- median(log(x), na.rm=T))), function(x) exp(median(x)))], cond))
  #option 2: sum up all intensities in log space -> high intensity rows gain more weight?!?
#return(setNames(cons[, lapply(.SD, function(x) exp(sum(log(x))))], cond)) #problem in stoich
-> exponents too high!
  #return(setNames(cons[, lapply(.SD, function(x) exp(sum(log(x))/length(x)))], cond))
#option 2b: sum all intensities in normal intensity space -> put higher weight on high
intensities
  return(setNames(cons[, lapply(.SD, function(x) sum(x))], cond))
  #option 3: mean or median of all intensities in log space -> high intensity rows gain
more weight?!?
  #return(setNames(cons[, lapply(.SD, function(x) exp(mean(log(x))))], cond))
#return(setNames(cons[, lapply(.SD, function(x) exp(median(log(x))))], cond))
}

if(par.level==0){ print("Done: Preparation for site-level collapse") }
if(par.level==1){ print("Done: Preparation for localized peptide-level collapse") }
if(par.level==2){ print("Done: Preparation for modification specific peptide-level collapse")
}


#initiate parallel processing
cl <- makeCluster(par.CPU)
registerDoParallel(cl)

#start collapse: include localization
if(par.level.col %in% c(0,1,3)){
  #load function
  consdt.pept.num <- function(temp, cond, type){
    #remove all entries without or with zero-length keycol entries
    temp <- temp[!is.na(PTM_collapse_key) & nchar(PTM_collapse_key)>0,]
    #need at least two valid entries per row to consider further
    temp <- temp[temp[, apply(.SD, 1, function(x) length(which(!is.na(x)))) >1, .SDcols
= cond],]
```

```
    #write number of entries per keycol group
    temp[, PTM_collapse_key_num := .N, by = PTM_collapse_key]

    #perform consolidation in chunks devided by PTM_collapse_key, rbind
with PTM_collapse_key_entries equal to one, and overwrite temp
    if(type==0){
      #perform linear modelling
      temp <- copy(cbind(temp[, consolidate(cons = .SD, cond = cond), .SDcols =
cond, by=PTM_collapse_key],
                         temp[, .(PTM_localization = max(PTM_localization, na.rm=T)),
by=PTM_collapse_key][,-1],
                         temp[, lapply(.SD, function(x) paste(unique(x), collapse=";")),
                              .SDcols = setdiff(names(temp), c("PTM_collapse_key",
"PTM_localization", cond)), by=PTM_collapse_key][,-1]))
    }
    if(type==1){
      #simply add intensities
      temp <- copy(cbind(temp[, lapply(.SD, function(x){ sum(x, na.rm = T) }), .SDcols =
cond, by=PTM_collapse_key],
                         temp[, .(PTM_localization = max(PTM_localization, na.rm=T)),
by=PTM_collapse_key][,-1],
                         temp[, lapply(.SD, function(x) paste(unique(x), collapse=";")),
                              .SDcols = setdiff(names(temp), c("PTM_collapse_key",
"PTM_localization", cond)), by=PTM_collapse_key][,-1]))

      #re-delete all 0s in columns that were generated by summing function
      for(col in cond.names){
        temp[get(col)==0, paste(col) := NA]
      }
    }

    #export temp into global environment
    return(temp)
  }

  #parallel collapse
  data <- copy(rbindlist(foreach(testp=subdt,.packages=c("data.table")) %dopar%
    { consdt.pept.num(temp=testp, cond=cond.names, type=par.agg)
  }))

  #overwrite all -Inf PTM_localization that occured from rows without non-missing
  localizations data[is.infinite(PTM_localization), PTM_localization := NA]

  print("Done: Collapse including maximum localization probability")
}

#start collapse: ignore localization
if(par.level.col == 2){
  #load function
  consdt.pept.num <- function(temp, cond, type){
    #remove all entries without or with zero-length keycol entries
    temp <- temp[!is.na(PTM_collapse_key) & nchar(PTM_collapse_key)>0,]

    #need at least two valid entries per row to consider further
    temp <- temp[temp[, apply(.SD, 1, function(x) length(which(!is.na(x)))) >1, .SDcols
= cond],]

    #write number of entries per keycol group
    temp[, PTM_collapse_key_num := .N, by = PTM_collapse_key]
```

```
    #perform consolidation in chunks devided by PTM_collapse_key, rbind
with PTM_collapse_key_entries equal to one, and overwrite temp
    if(type==0){
      #perform linear modelling
      temp <- copy(cbind(temp[, consolidate(cons = .SD, cond = cond), .SDcols =
cond, by=PTM_collapse_key],
                         temp[, lapply(.SD, function(x) paste(unique(x), collapse=";")),
                              .SDcols = setdiff(names(temp), c("PTM_collapse_key", cond)),
by=PTM_collapse_key][,-1]))
    }
    if(type==1){
      #simply add intensities
      temp <- copy(cbind(temp[, lapply(.SD, function(x){ sum(x, na.rm = T) }), .SDcols =
cond, by=PTM_collapse_key],
                         temp[, lapply(.SD, function(x) paste(unique(x), collapse=";")),
                              .SDcols = setdiff(names(temp), c("PTM_collapse_key", cond)),
by=PTM_collapse_key][,-1]))

      #re-delete all 0s in columns that were generated by summing function
      for(col in cond.names){
        temp[get(col)==0, paste(col) := NA]
      }
    }

    #export temp into global environment
    return(temp)
  }

  #parallel collapse
  data <- copy(rbindlist(foreach(testp=subdt,.packages=c("data.table")) %dopar%
    { consdt.pept.num(temp=testp, cond=cond.names, type=par.agg)
  }))

  print("Done: Collapse ignoring localization probability")
}

#close parallel processing
stopCluster(cl)







#PTM localization probability filtering
if(par.level.col %in% c(0,1,3)){
  if(par.level.cutoff>0){
    print(paste("Cave: Filtered ", data[!(PTM_localization>=par.level.cutoff |
    is.na(PTM_localization)), .N], " rows below cutoff of ", par.level.cutoff,
                                        sep=""))

    #filter all rows below cutoff
    data <- data[PTM_localization>=par.level.cutoff | is.na(PTM_localization),
  ] }
}


#amino acid type extrection if site-level
if( par.level == 0){
  data[, PTM_0_aa := gsub("^.*_(.).*_M[1-3]$", "\\1", PTM_collapse_key,
```

```
perl=T)] }


#PTM motif sequence matching only if site-level & FASTA file path defined
if( par.level == 0 &
if(exists("par.FASTA.file")){!identical(par.FASTA.file,
character(0))}else{F} ){
  #par.FASTA.parse <- ".*\\|(.*)\\|.*"
  #par.FASTA.parse <- ".*GN=([^ ]*) .*"

  #read fasta file into R
  fasta <- readAAStringSet(par.FASTA.file)
  fasta <- data.table(header = names(fasta), ident_seq = paste(fasta))
  fasta[, ident := gsub(par.FASTA.parse, "\\1", header, perl=T)]
  fasta[, pat_match := grepl(par.FASTA.parse, header)]
  #keep only rows with parse pattern
  fasta <- fasta[pat_match==T, ]
  fasta[, header := NULL]
  fasta[, pat_match := NULL]

  #need to first check if par.level.col.genprot are part of FASTA file
  data[, ident.first := gsub("^;(.*)$", "\\1", get(par.level.col.genprot),
  perl=T)] data[, ident.first := gsub("^([^;]+);.*", "\\1", ident.first, perl=T)]
  data[, ident.fasta.match := get(par.level.col.genprot) %in% fasta$ident]
print(paste("Cave: could not map ", data[ident.fasta.match==F, .N], " rows out of ", data[,
.N], " for FASTA info", sep=""))

  #map fasta information onto data
  data <- merge(x=data, y=fasta, by.x="ident.first", by.y="ident", all.x=T, all.y=F)

  #re-extract PTM position from PTM_collapse_key
  data[, PTM_pos := as.integer(gsub("^.*_.(.*)_M.$", "\\1", PTM_collapse_key, perl=T))]

  #create PTM sequence window, starting by determining sub-cases
  data[, seq_end_length := nchar(ident_seq)-PTM_pos]

  #if PTM_pos >=16 AND seq_end_length >=15
  data[PTM_pos>=16 & seq_end_length>=15, PTM_seq := substr(ident_seq,
(as.integer(PTM_pos)-15), (as.integer(PTM_pos)+15))]

  #if PTM_pos <16 AND seq_end_length >=15
  data[PTM_pos<16 & seq_end_length>=15, PTM_seq :=
         mapply(function(x,y){paste(paste(rep("_", (16-x)), collapse=""), substr(y,
1, (as.integer(x)+15)), sep="")}, x=PTM_pos, y=ident_seq)]

  #if PTM_pos >=16 AND seq_end_length <15
  data[PTM_pos>=16 & (seq_end_length<15 & seq_end_length>=0), PTM_seq :=
         mapply(function(x,y){paste(substr(y, (as.integer(x)-15), (nchar(y))),
paste(rep("_", (15-(nchar(y)-x))), collapse=""), sep="")}, x=PTM_pos, y=ident_seq)]

  #if PTM_pos <16 AND seq_end_length <15
  data[PTM_pos<16 & (seq_end_length<15 & seq_end_length>=0), PTM_seq :=
         mapply(function(x,y){paste(paste(rep("_", (16-x)), collapse=""),
                                    substr(y, (as.integer(x)-15), (nchar(y))), paste(rep("_",
(15-(nchar(y)-x))), collapse=""), sep="")}, x=PTM_pos, y=ident_seq)]

  data[, seq_end_length := NULL]
  data[, ident.fasta.match := NULL]
  data[, ident_seq := NULL]
  data[, PTM_pos := NULL]
```

```
  print("Done: PTM sequence motif extraction")
}



#initiate stoichiometry calculation
if(par.level==1 & if(exists("par.level.stoich")){par.level.stoich == 0}else{F}){

  #create PTM_stoich_key sequence, deleting target PTM from
  PTM_collapse_key data[, PTM_stoich_key := gsub(par.PTM[1], "",
  PTM_collapse_key, fixed=T)] setkey(data, PTM_stoich_key, PTM_0_num,
  PTM_collapse_key)

  #prepare list of subset data dt, which are passed to the cores
  individually if(par.cond == 0L){
    if(par.level.col %in% c(0,1,3)){
      keep <- c(cond.names, setdiff(else.name, par.cond.col), "PTM_0_num",
"PTM_group", "PTM_collapse_key", "PTM_collapse_key_num", "PTM_stoich_key",
                "PTM_localization")
    }
    if(par.level.col == 2){
      keep <- c(cond.names, setdiff(else.name, par.cond.col), "PTM_0_num",
"PTM_group", "PTM_collapse_key", "PTM_collapse_key_num", "PTM_stoich_key")
    }
  } else if(par.cond == 1L){
    if(par.level.col %in% c(0,1,3)){
      keep <- c(cond.names, else.name, "PTM_0_num", "PTM_group",
"PTM_collapse_key", "PTM_collapse_key_num", "PTM_stoich_key",
                "PTM_localization")
    }
    if(par.level.col == 2){
      keep <- c(cond.names, else.name, "PTM_0_num", "PTM_group",
"PTM_collapse_key", "PTM_collapse_key_num", "PTM_stoich_key")
    }
  }



  #create vector describing split points for dataset between collapsing
  units RAWseq_vec <- data[, unique(PTM_stoich_key)]
  vec <- 1L
  for(col in RAWseq_vec[round(quantile(1:length(RAWseq_vec), probs = seq(0,1,length.out
= par.CPU+1)[-1]),0)]){
    vec <- c(vec, grep(col, data$PTM_stoich_key, fixed =
T)[length(grep(col, data$PTM_stoich_key, fixed=T))])
  }

  #create subdt: 8 (=number of cores) sub-data tables within list to be passed into
function separately
  subdt <- list(data[1:vec[2], .SD, .SDcols=keep])
  for(col in 2:par.CPU){
    subdt <- c(subdt, list(data[(vec[col]+1):vec[col+1], .SD,
  .SDcols=keep])) }

  print("Done: Stoichiometry preparation")

  # #check if total number of rows in subdt fits those in test
  # sum(unlist(lapply(subdt, nrow)))
  # #check if first and last entry different
  # for(i in 1:(par.CPU-1)){
```

```
  # print(paste(subdt[[i]][.N, .(PTM_stoich_key)]==subdt[[i+1]][1, .(PTM_stoich_key)], #
subdt[[i]][.N, .(PTM_stoich_key)], subdt[[i+1]][1, .(PTM_stoich_key)], sep=" ; "))
  # }

  #load functions
  rlmfun <- function(x, skiperror=T, multiphos=2, PTMnum = PTMnum){
    #to avoid singularity, need to have overfit -> at least as many valid value matches
over all peptides as conditions
    #eg, if we have three occseq peptide variants, we need at least 3 conditions with
complete matches over all occseq peptide variants!
    #initiate logical selv vector which will define number of rows that can be used for
rlm modeling via TRUE, and excludes duplicate peptide rows
    selv <- !duplicated(as.data.frame(x))
    #tried to solve problem with duplicate rows leading to singularity, but also kicks
out working rows -> disregarded for now, using error trap instead
    # if(length(which(apply(x, 2, function(y) !any(duplicated(y))
    )))>=nrow(x)){ # selv <- !duplicated(x)
    # } else {
    # selv <- c(T, rep(F, (nrow(x)-1)))
    # }

    #multiphos: if 1, should only consider occgrp with PTMnum equaling 2 different
unique PTMnum; if 0, should only equal 2 different total PTMnum; if 2 use all
    if(multiphos==1){
      if(length(unique(PTMnum))!=2){
        x[!(PTMnum == sort(unique(PTMnum))[1]| PTMnum == sort(unique(PTMnum))[2])] <-
      NA }
    }
    if(multiphos==0){
      if(length(unique(PTMnum))!=2 | length(PTMnum)!=2){
        #calculate lowest two PTMnum values and for each individually pick the matrix row
with the highest medium intensity -> overwrite all other entries with NA
        x[!(rowMedians(x, na.rm=T)==sort(rowMedians(x[PTMnum ==
sort(unique(PTMnum)) [1],,drop=F], na.rm=T), decreasing = T)[1] |
              rowMedians(x, na.rm=T)==sort(rowMedians(x[PTMnum ==
sort(unique(PTMnum)) [2],,drop=F], na.rm=T), decreasing = T)[1])] <- NA
      }
    }

    # #check if there are duplicate values between rows; if yes, add rnorm onto rows to
enable calculation
    # if(any(apply(as.matrix(bla[, .SD, .SDcols=condi]), 2, function(x)
any(duplicated(x))))) {}
    # duplicated(as.matrix(bla[, .SD, .SDcols=condi]))
    # t(apply(as.matrix(bla[, .SD, .SDcols=condi]), 1, function(x) x+rnorm(n=length(x),
mean= (median(x, na.rm=T)/100))))

    #count number of conditions with full matches and check if at least equal to number
of occseq
    if(!(length(which(apply(x, 2, function(x) length(which(!is.na(x))))==nrow(x))) >=
nrow(x))) {
      #need to remove x-row with lowest number of non-NA-values and check
      again #if all values removed, then no calculation possible
      #at same time remember their original position, so that reconstruction of slope
for different peptide rows is possible
      nanv <- apply(x, 1, function(x) length(which(!is.na(x))))

      #create permanent selection vector for formula creation subsetting
      selv <- nanv>min(nanv[selv], na.rm=T) & selv

      #check if at least 2 positive lines remaining in selv, because otherwise rlm will not
be possible
```

```
      if(length(which(selv))>1){
        y <- x[(1:nrow(x))[selv],]

        #repeat reducing selv vector if matching conditions still not met
        while(!(length(which(apply(y, 2, function(x) length(which(!is.na(x))))==nrow(y)))
>= nrow(y))){
          #create second selection vector, which is then compared to initial x-based
selection vector and overwrite selv
          nanv2 <- apply(y, 1, function(x) length(which(!is.na(x))))
          selv <- nanv>min(nanv2, na.rm=T) & selv
          y <- x[(1:nrow(x))[selv],]

          #check again if at least 2 positive lines remaining in selv, because otherwise
rlm will not be possible
          if(length(which(selv))<2){break}
        }
      }
    }

    #check if more than one row existent in selv, since at least two matching occseq
entries are necessary for rlm
    if(length(which(selv))>1){
      #create function and named vector selector as string

      f <- paste("x[", which(selv)[1], ",]~x[", which(selv)[2], ",]", sep="")
      if(length(which(selv)) > 2){
        for(repn in which(selv)[c(-1,-2)]){
          f <- paste(f, "+x[", repn ,",]", sep="")
          #g <- c(g, paste("x[",repn,", ]", sep=""))
        }
      }
      #should NOT add plus 0, as entries cannot be expected to correlate through
      0 #f <- paste(f, "+0", sep="")

      g <- c("x[1, ]", "x[2, ]")
      if(nrow(x)>2){
        for(repn in 3:nrow(x)){
          g <- c(g, paste("x[",repn,", ]", sep=""))
        }
      }

      #perform rlm calculation and return data table
      #importantly, cannot call value by name if rlm models only two entries, then have
to construct vector manyally with slope in between
      if(length(which(selv))==2){
        if(skiperror){
          return(data.table(slope = as.numeric(c(rep(NA, (which(selv)[2]-1)),
                                                  tryCatch(coefficients(summary(do.call("rlm",
list(as.formula(f), maxit=1000))))[-1,"Value"],
                                                           error=function(e){print("RLM error
occured, returning NA; see FAQ");NA}),
                                                  rep(NA, (length(selv)-which(selv)[2])))),
calc = selv))
        } else {
          return(data.table(slope = as.numeric(c(rep(NA, (which(selv)[2]-1)),
coefficients(summary(do.call("rlm", list(as.formula(f), maxit=1000))))[-1,"Value"],
                                                 rep(NA, (length(selv)-which(selv)[2])))),
calc = selv))
        }

      } else {
```

```
        if(skiperror){
          return(data.table(slope = tryCatch(coefficients(summary(do.call("rlm",
list(as.formula(f), maxit=1000))))[,"Value"][g],
                                             error=function(e){print("RLM error occured,
returning NA; see FAQ");as.numeric(rep(NA, nrow(x)))}), calc = selv))
        } else {
          return(data.table(slope =
as.numeric(coefficients(summary(do.call("rlm", list(as.formula(f),
maxit=1000))))[,"Value"][g]), calc = selv))
        }

      }

    } else {
      #if only one row, return NA data table
      return(data.table(slope = as.numeric(rep(NA, nrow(x))), calc = rep(F,
    nrow(x)))) }

  }

  stoichiometry <- function(pepi, calc, cond){
    #check if enough calc entries present, if yes execute occ calculation
    stoi <- setNames(as.data.table(rep(list(rep(as.numeric(NA), length(calc))),
length(cond))), gsub("^(.*)$", "Occ_\\1", cond))

    if(length(which(calc))>1){
      #first entry, calculate occ and write into first medlist evd group
      #for the first entry: except target entry, times own slope; two slopes plus, one
slope minus
      stoi[which(calc)[1], ] <- pepi[,
.SD[which(calc)[1],-1]-(.SD[which(calc)[2],-
1]*as.numeric(.SD[which(calc)[2],1]))]
      if(length(which(calc))>2){
        for(repn in which(calc)[-c(1,2)]){stoi[which(calc)[1], ] <- stoi[which(calc)[1], ]
- pepi[, .SD[repn,-1]*as.numeric(.SD[repn,1])]}
      }
      stoi[which(calc)[1], gsub("^(.*)$", "Occ_\\1", cond) := pepi[, .SD[which(calc)[1],-1]]
/ .SD]
      #for all subsequent entries, calculate occ and write into subsequent evd groups
#for other entries: except target entry, times own slope, divided by target slope (y
entry exception: only divided by target); two slopes plus, one slope minus for(repn2 in
which(calc)[-1] ){
        stoi[repn2, ] <- pepi[, .SD[repn2,-1]-(.SD[which(calc)[1],-
1]/as.numeric(.SD[repn2,1]))]

        if(length(which(calc))>2){
          for(repn in which(calc)[-c(1,grep(repn2, which(calc)))]){stoi[repn2, ] <-
stoi[repn2, ] + pepi[,
.SD[repn,-1]*as.numeric(.SD[repn,1])/as.numeric(.SD[repn2,1])]} }

        #calculate occupancies and write results into medlist for subsequent entries
#multiply with if testing for slope being NA even though calc is TRUE, which then needs to
set all stoi to NA for this entry
        stoi[repn2, gsub("^(.*)$", "Occ_\\1", cond) := pepi[, .SD[repn2,-
1]*if(is.na(as.numeric(.SD[repn2,1]))){NA}else{1}] / .SD]
      }
      return(stoi)
    } else {
      #if not enough calc entries present, return NA data table
      return(stoi)
    }
```

```
  }

  stoichdt <- function(temp, cond.names){
    #calculate rlm and store as model, then report back occupancies
    temp <- copy(cbind(temp[, .SD, by=PTM_stoich_key], temp[,
rlmfun(as.matrix(.SD), skiperror=T, multiphos=2, PTMnum=as.numeric(PTM_0_num)),
                                                              .SDcols = cond.names,
by=PTM_stoich_key][,-1]))

    #calculate stoichiometry values for each occgrp
    temp <- copy(cbind(temp[, .SD, by=PTM_stoich_key], temp[, stoichiometry(.SD,
calc, cond.names), .SDcols=c("slope", cond.names), by=PTM_stoich_key][,-1]))

    #delete illegal
    for (col in gsub("^(.*)$", "Occ_\\1", cond.names)){ temp[get(col)<0 | get(col)>1, (col)
:= NA] }

    #calculate number of PTM_stoich_key sequences per PTM_stoich_key grp and number of PTMs
per PTM_stoich_key grp
    temp[calc==T, PTM_stoich_key_num := .N, by=PTM_stoich_key]
    temp[calc==T, PTM_stoich_key_PTMnum := paste(sort(unique(PTM_0_num)),
collapse=";"), by=PTM_stoich_key]

    #return data table to top level
    return(temp)
  }

  #initiate parallel processing
  cl <- makeCluster(par.CPU)
  registerDoParallel(cl)

  #parallel stoichiometry calculation
  data <- copy(rbindlist(foreach(testp=subdt,.packages=c("data.table", "MASS")) %dopar%
    { stoichdt(temp=testp, cond.names=cond.names)
  }))

  #close parallel processing
  stopCluster(cl)

  cond.names <- c(cond.names, gsub("^(.*)$", "Occ_\\1", cond.names))

  print("Done: Stoichiometry calculation")
}
#write res into main including row names
maind <- as.data.frame(data[, .SD, .SDcols = cond.names])
rownames(maind) <- paste("Row.", seq(1,nrow(maind),1), sep="")
mdata.main <- maind


#prepare writing additional columns into annotCols
keep <- character()

#main columns
if(par.level.col %in% c(0,1,3)){
  keep <- c(keep, else.name, "PTM_0_num", "PTM_group",
"PTM_collapse_key", "PTM_collapse_key_num", "PTM_localization")
} else if(par.level.col == 2){
  keep <- c(keep, else.name, "PTM_0_num", "PTM_group",
"PTM_collapse_key", "PTM_collapse_key_num")
}
```

```
if(if(exists("par.level.stoich")){par.level.stoich == 0}else{F}){
  #collapse and stoichiometry
  keep <- c(keep, "PTM_stoich_key", "PTM_stoich_key_num", "PTM_stoich_key_PTMnum")

} else if( par.level == 0 &
if(exists("par.FASTA.file")){!identical(par.FASTA.file, character(0))}else{F} ){
  #collapse and PTM motif sequence
  keep <- c(keep, "PTM_seq", "PTM_0_aa")

} else if(par.level == 0){
  #collapse site-level only
  keep <- c(keep, "PTM_0_aa")

} else {
  #collapse only

}

#if grouping performed, delete par.cond.col from keep list, since does not exist
anymore if(par.cond == 0){
  keep <- setdiff(keep, par.cond.col)
}

#write additional columns into annotCols
c.annot <- as.data.frame(data[, .SD, .SDcols = keep])
rownames(c.annot) <- paste("Row.", seq(1,nrow(c.annot),1), sep="")
mdata.annotCols <- c.annot

findf <- cbind(mdata.main, mdata.annotCols)
write.table(findf, outFile, sep="\t", row.names = FALSE)
```
