# Supplementary figures and images for: In-depth mass-spectrometry reveals phospho-RAB12 as a blood biomarker of G2019S LRRK2-driven Parkinson’s disease
Source: Brain. 2024 Dec 20;148(6):2075–92. doi: 10.1093/brain/awae404 (PMC12129731; doi:10.1093/brain/awae404)

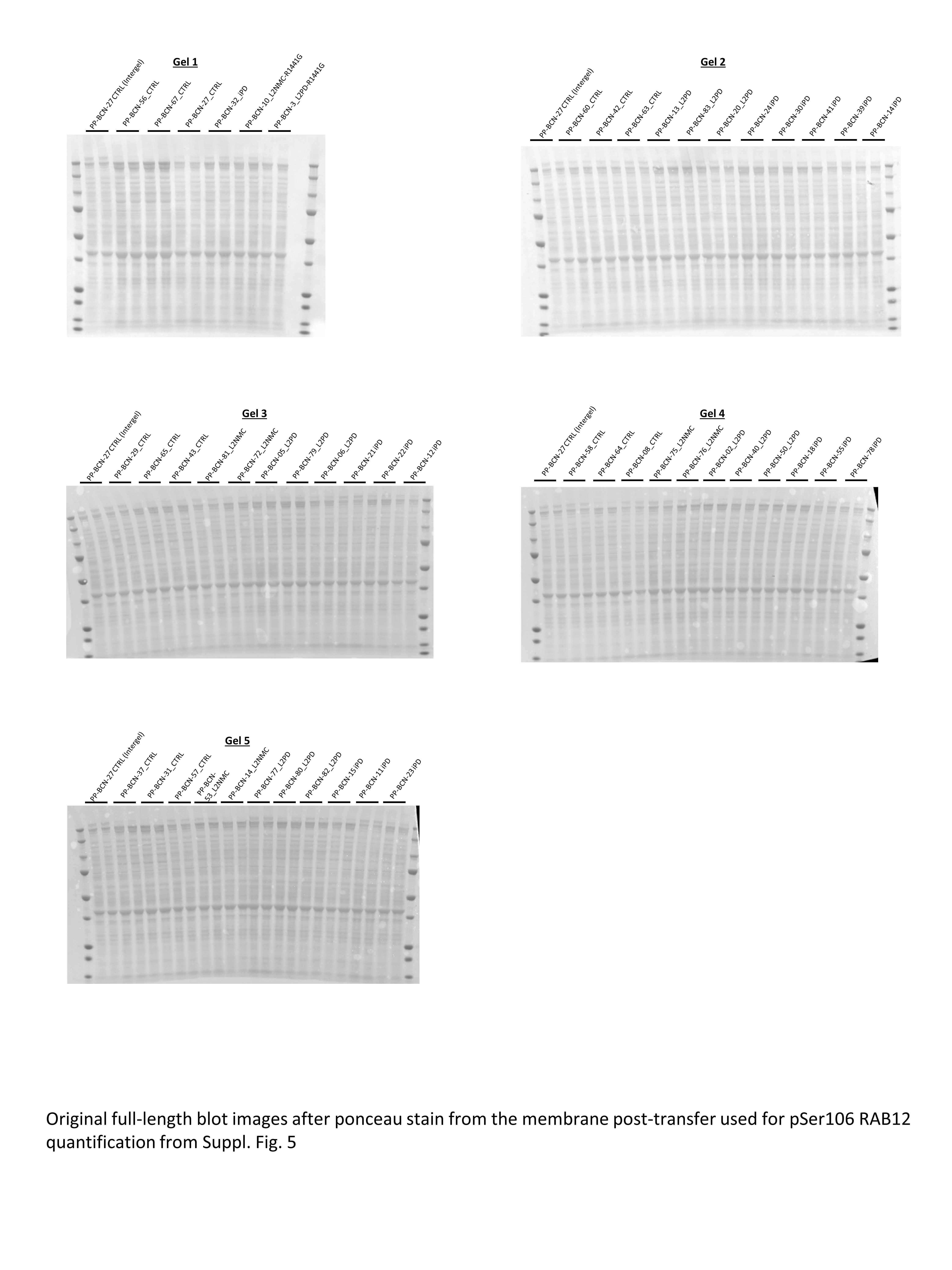

Supplement: awae404_Supplementary_Data [file awae404_supplementary_data.zip › brain-2024-01397-File010.tif]
